# Supplementary material for: Comparison of Histone H3K4me3 between IVF and ICSI Technologies and between Boy and Girl Offspring
Source: Int J Mol Sci. 2021 Aug 9;22(16):8574. doi: 10.3390/ijms22168574 (PMC8395251; doi:10.3390/ijms22168574)
Supplement: Supplementary file 1 [file ijms-22-08574-s001.zip › ijms-1331788-supplementary.pdf]

Supplementary Materials for

Comparison of histone H3K4me3 between IVF and ICSI technologies and between boy and girl offspring

Huixia Yang [1], Zhi Ma [1], Lin Peng [1], Christina Kuhn [1,2], Martina Rahmeh [1], Sven Mahner [1], Udo Jeschke [1,2]*, Viktoria von Schönfeldt [1]

[1]Department of Obstetrics and Gynecology, University Hospital, Ludwig-Maximilians-University, Munich, Germany; [2]Department of Obstetrics and Gynecology, University Hospital Augsburg, Augsburg, Germany.

* Correspondence: udo.jeschke@med.uni-muenchen.de; University Hospital Augsburg, Stenglinstrasse 2, 86156, Augsburg, Germany; Tel.: +49-821-400-165505

This file includes:

Supplementary materials and methods for low-oxygen culture and intermittent hyperoxia exposure.

Supplementary Figure S1. PPI network based on the genes with deH3K4me3 from ICSI-boys.

Supplementary Figure S2. PPI network based on the genes with deH3K4me3 from ICSI-girls.

Supplementary Figure S3. Venn diagrams.

Supplementary Table S1. The list of imprinted genes with entrezID.

Supplementary Table S2. The list of cardiovascular-disease-associated genes with entrezID.

Supplementary Table S3. Clinical characteristics of the study subjects.

Supplementary Table S4. Genes with deH3K4me3 in IVF and ICSI children.

Supplementary Table S5. Cardiovascular-disease-associated genes with deH3K4me3 in ICSI children.

Supplementary Table S6. Transcription factor analysis for genes with H3K4me3 enrichment in 4-cell, 8-cell, and ICM stages.

Supplementary Table S7. Literature retrieval results for enzymes/proteins that were potentially involved in regulating H3K4me3.

Supplementary Table S8. Transcription factor lists in the Venn diagrams.

Supplementary materials and methods

Low-oxygen culture and intermittent hyperoxia exposure

We used HTR-8/SVneo cells (passage numbers 25 to 32). Before treatment, we spread cells evenly on plates containing RPMI medium 1640 + GlutaMAX (Gibco) + 10% fetal bovine serum (Gibco) without antibiotics/antimycotics and maintained overnight in a standard incubator. For 'low-oxygen culture': we placed cells with various low-oxygen culture durations (0.5, 1, 2, 4 hours, 1, 2, 3, 4 days) into a tri-gas incubator (37 °C, could be set with a continuous flow of a hypoxic gas mixture containing 5% CO_2, 1% / 5% O_2, and N_2) in reverse chronological order (i.e., we placed cells with 4-day low-oxygen exposure first put into the tri-gas incubator, and cells with 0.5-hour low-oxygen culture period were the last). We removed cells with different low-oxygen culture durations simultaneously. We processed the low-oxygen culture in 1% and 5% O_2 using the same procedure for various oxygen tensions. For 'intermittent hyperoxia exposure': we placed cells in a tri-gas incubator (37°C, 5% CO_2, 5% O_2, and N_2) for 8 hours, in a standard incubator for 16 hours, then in a tri-gas incubator (37 °C, 5% CO_2, 5% O_2, and N_2) for 8 hours. We compared the results of intermittent hyperoxia exposure with persistent atmospheric oxygen culture (20% O_2 for 32 hours) and continuous low-oxygen culture (5% O_2 for 32 hours). To avoid air exposure, we placed cells with different low-oxygen culture periods in the corresponding targeted chambers. For negative controls (atmospheric oxygen culture), we always grew cells in a standard incubator. We collected cells when they were 80% to 90% confluent. We repeated each experiment three times.

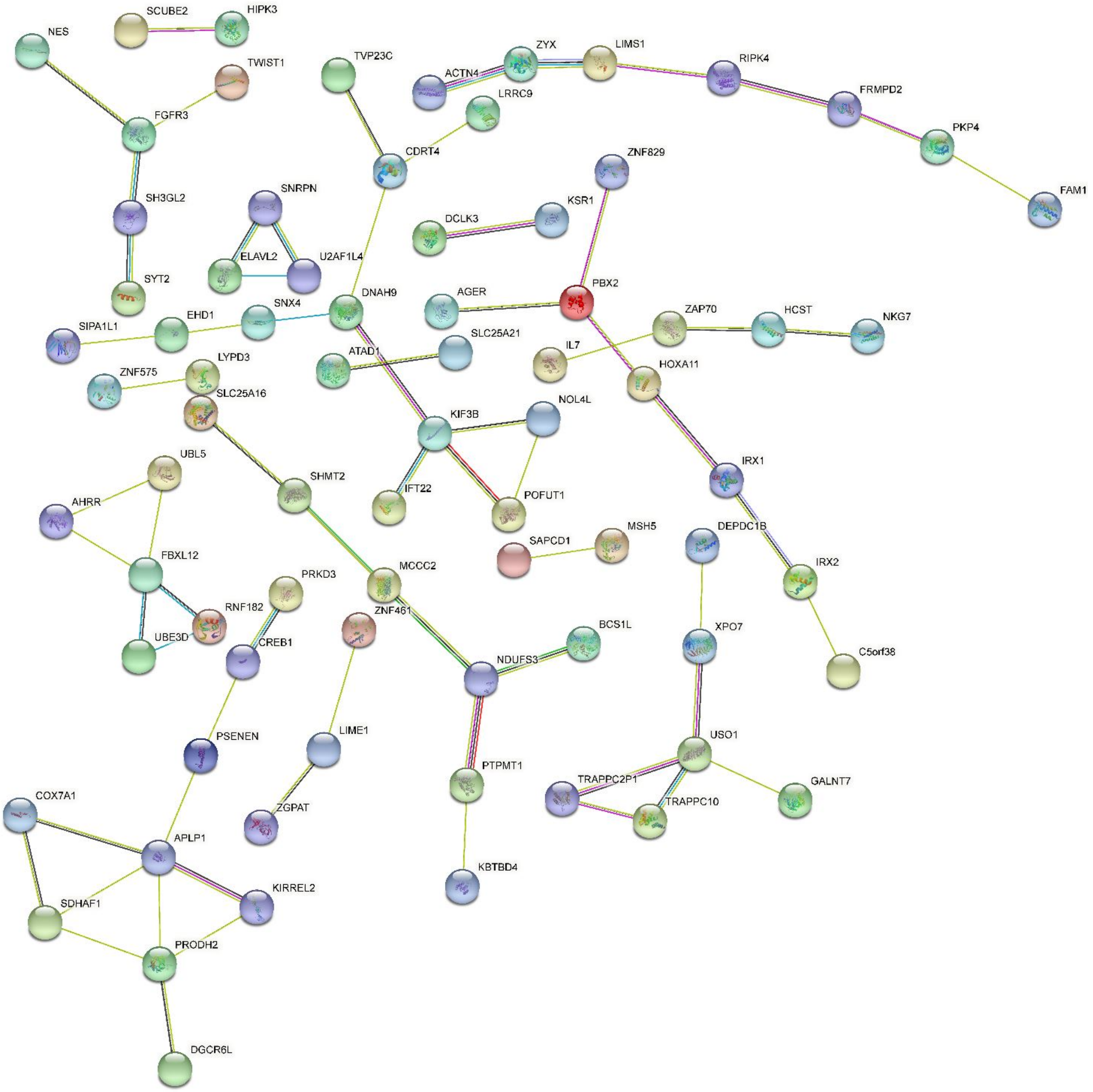

Supplementary Figure S1. PPI network based on the genes with deH3K4me3 from ICSI-boys. The minimum required interaction score was set as 0.4. PPI, protein-protein interaction; deH3K4me3, differentially enriched tri-methylated-histone-H3-lysine-4; ICSI, intracytoplasmic sperm injection.

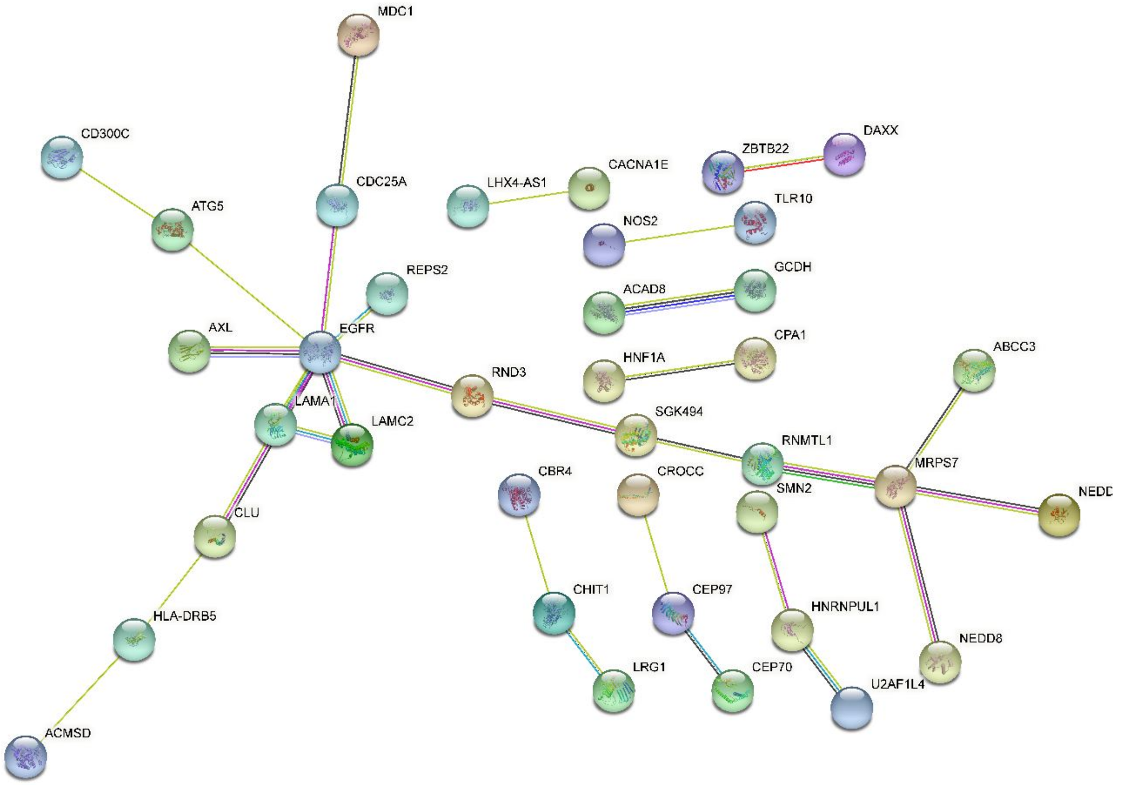

Supplementary Figure S2. PPI network based on the genes with deH3K4me3 from ICSI-girls. The minimum required interaction score was set as 0.4.

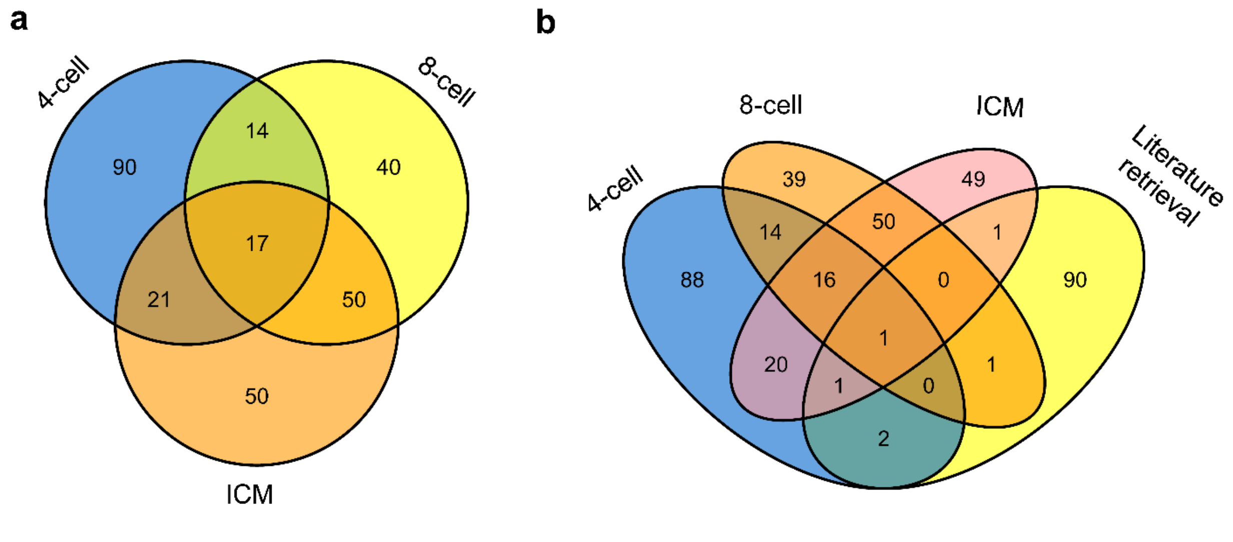

Supplementary Figure S3. Venn diagrams. (a) Overlapping results of transcription factor analysis for genes with H3K4me3 enrichment in 4-cell, 8-cell, and ICM stages. **(b)** Overlapping results of literature retrieval and transcription factor analysis for genes H3K4me3 enrichment in 4-cell, 8-cell, and ICM stages. ICM, inner cell mass.

Supplementary Table S1. The list of imprinted genes with entrezID.

Gene Symbol	Entrez ID	Gene Symbol	Entrez ID	Gene Symbol	Entrez ID
DIRAS3	9077	*SLC22A18*	5002	*TCEB3C*	162699
RNU5D-1	26830	*H19*	283120	*PARD6G*	84552
TP73	7161	*PHLDA2*	7262	*DNMT1*	1786
LRRTM1	347730	*IGF2*	3481	*ZIM2*	23619
ZDBF2	57683	*CDKN1C*	1028	*PEG3*	5178
GPR1	2825	*KCNQ1*	3784	*MIMT1*	100073347
NAP1L5	266812	*IGF2AS*	51214	*MIR371A*	442916
ERAP2	64167	*ANO1*	55107	*NLRP2*	55655
RHOBTB3	22836	*ZC3H12C*	85463	*PEG3-AS1*	100169890
VTRNA2-1	100126299	*NTM*	50863	*PSIMCT-1*	100101490
ADTRP	84830	*ST8SIA1*	6489	*BLCAP*	10904
FAM50B	26240	*RBP5*	83758	*NNAT*	4826
PXDC1	221749	*HNF1A*	6927	*GDAP1L1*	78997
AIM1	829022	*ATP5F1EP2*	432369	*SGK2*	10110
LIN28B	389421	*RB1*	5925	*GNAS*	2778
PLAGL1	5325	*ESR2*	2100	*L3MBTL1*	26013
HYMAI	57061	*SMOC1*	64093	*GNASAS*	149775
SLC22A2	6582	*MEG3*	55384	*MIR298*	100126296
SLC22A3	6581	*DIO3*	1735	*SANG*	149775
GRB10	2887	*DLK1*	8788	*MIR296*	407022
DDC	1644	*MEG8*	79104	*DSCAM*	1826
GLI3	2737	*SNORD113-1*	767561	*DGCR6L*	85359
HECW1	23072	*SNORD114-1*	767577	*DGCR6*	8214
HOXA4	3201	*RTL1*	388015	*FAM99A*	387742
RAC1	5879	*DIO3OS*	64150		
PEG10	23089	*MAGEL2*	54551		
MAGI2	9863	*UBE3A*	7337		
SGCE	8910	*MKRN3*	7681		
PPP1R9A	55607	*NPAP1*	23742		
TFPI2	7980	*ATP10A*	57194		
DLX5	1749	*SNORD109B*	338429		
CCDC71L	168455	*SNORD115-48*	100033822		
CPA4	51200	*SNORD116*	692236		
MEST	4232	*SNRPN*	6638		
COPG2IT1	53844	*SNORD109A*	338428		
MESTIT1	317751	*SNORD108*	338427		
KLF14	136259	*SNORD107*	91380		
SVOPL	136306	*PWAR6*	100506965		
DLGAP2	9228	*PWCR1*	692236		
KCNK9	51305	*NDN*	4692		
ZFAT	57623	*SNORD64*	347686		
PEG13	359809	*SNURF*	8926		
ZFAT-AS1	594840	*RASGRF1*	5923		
GLIS3	169792	*IRAIN*	104472848		
WT1-AS	51352	*NAA60*	79903		
WT1	7490	*ZNF597*	146434		
KCNQ1OT1	10984	*CMTM1*	113540		
OSBPL5	114879	*ZFP90*	146198		
KCNQ1DN	55539	*TP53*	7157		
INS	3630	*ZNF396*	252884		

Supplementary Table S2. The list of cardiovascular-disease-associated genes with entrez ID

Gene Symbol	Entrez ID	Gene Symbol	Entrez ID	Gene Symbol	Entrez ID
ACLY	47	*GLRX3*	10539	*CSF1*	1435
CASR	846	*UBD*	10537	*CHRM1*	1128
CD2	914	*CCNH*	902	*AGT*	183
CHRNA4	1137	*PDK2*	5164	*AHR*	196
COX4I1	1327	*STX1A*	6804	*VEGFA*	7422
GGCX	2677	*GHRL*	51738	*GRIN2B*	2904
CCND1	595	*NOS2*	4843	*ADRA2B*	151
EPHA3	2042	*ARC*	23237	*PGR*	5241
INPPL1	3636	*TGFB2*	7042	*HDAC3*	8841
EPAS1	2034	*PDE2A*	5138	*PANK4*	55229
PFKM	5213	*STC1*	6781	*PITX2*	5308
CLDN1	9076	*SOAT2*	8435	*ADAM17*	6868
DLG4	1742	*TNFRSF11B*	4982	*PRKCB*	5579
CLDN3	1365	*SREBF2*	6721	*RAPGEF1*	2889
AQP3	360	*CKB*	1152	*IL6ST*	3572
CLCNKA	1187	*AVPR2*	554	*ICAM1*	3383
GUCY1A1	2982	*CDC42*	998	*SERPINE2*	5270
OPRL1	4987	*SLC9A3*	6550	*AKAP10*	11216
BHLHE40	8553	*IL1B*	3553	*RGS4*	5999
AVP	551	*COL1A2*	1278	*AQP1*	358
GCG	2641	*VCAM1*	7412	*AQP2*	359
AVPR1A	552	*CALCA*	796	*AQP5*	362
IGF1R	3480	*GNAI2*	2771	*BAX*	581
PENK	5179	*HSF1*	3297	*TP53*	7157
SLC6A6	6533	*ADORA2A*	135	*IGF1*	3479
ITGA1	3672	*CASP3*	836	*FGF2*	2247
IL13	3596	*GNAI3*	2773	*MET*	4233
INSR	3643	*HTR1B*	3351	*CDK5*	1020
FSTL1	11167	*PTGS2*	5743	*DRD1*	1812
QSOX1	5768	*TACR1*	6869	*PLD1*	5337
INA	9118	*DYNLL1*	8655	*CPS1*	1373
IGFBP1	3484	*CCN2*	1490	*CYP11B1*	1584
GNRH1	2796	*PLA2G2A*	5320	*LPL*	4023
GH1	2688	*KDR*	3791	*HGF*	3082
JAK1	3716	*ADORA2B*	136	*NOS1*	4842
PTH	5741	*SLC8A1*	6546	*CREBBP*	1387
LEP	3952	*PCSK9*	255738	*CD36*	948
BRCA1	672	*BDNF*	627	*CPT1B*	1375
NPPB	4879	*SGK1*	6446	*AGTR2*	186
MDK	4192	*DFFB*	1677	*TAC1*	6863
GHR	2690	*SPARC*	6678	*VDR*	7421
EGFR	1956	*GRK2*	156	*ERBB2*	2064
TIMP4	7079	*SOAT1*	6646	*MAPK14*	1432
NFIX	4784	*OLR1*	4973	*CFTR*	1080
PPARG	5468	*CCL2*	6347	*BGLAP*	632
ASIP	434	*CIT*	11113	*CAV1*	857
FGF7	2252	*DGAT1*	8694	*ABCC2*	1244
ESR2	2100	*EPHX2*	2053	*MAPK3*	5595
LCN2	3934	*INS*	3630	*GFAP*	2670
AKAP6	9472	*KCNA5*	3741	*ADORA1*	134

Supplementary Table S2 (continued)

Gene Symbol	Entrez ID	Gene Symbol	Entrez ID	Gene Symbol	Entrez ID
MMP2	4313	*SLC2A5*	6518	*CRHR2*	1395
ABCB1	5243	*POR*	5447	*TJP2*	9414
ESRRA	2101	*HTT*	3064	*NTF4*	4909
NPY	4852	*TXN*	7295	*HS3ST1*	9957
CYP4A11	1579	*NKX2-1*	7080	*MAS1*	4142
BMP4	652	*FTH1*	2495	*FGG*	2266
SOCS3	9021	*CYSLTR1*	10800	*MAP3K8*	1326
CHGA	1113	*NAT2*	10	*NRG1*	3084
CSPG4	1464	*ERBB4*	2066	*KIF3C*	3797
GRK3	157	*MSN*	4478	*ARRB2*	409
ANGPT2	285	*MFN2*	9927	*FBN1*	2200
CASP8	841	*MBTPS1*	8720	*HMGCR*	3156
EDN1	1906	*MAOB*	4129	*NPY5R*	4889
GAD1	2571	*ADARB1*	104	*HAND2*	9464
BTG2	7832	*BIRC3*	330	*CAMK1*	8536
SOD1	6647	*TKT*	7086	*NPR3*	4883
IL2	3558	*EGLN3*	112399	*RPS6KB1*	6198
SDC1	6382	*HTRA1*	5654	*PPP1CB*	5500
RGS5	8490	*IGF2R*	3482	*PLEC*	5339
CYP1A1	1543	*LYN*	4067	*RB1*	5925
SOD2	6648	*INHBA*	3624	*CCN1*	3491
ANXA1	301	*PRLHR*	2834	*PSEN1*	5663
BDKRB1	623	*CAT*	847	*CPB2*	1361
SDC4	6385	*HAS2*	3037	*AHCY*	191
NPPA	4878	*NOTCH3*	4854	*JAK2*	3717
MT3	4504	*NR4A1*	3164	*SLC8A2*	6543
HRAS	3265	*PTK2*	5747	*GSK3A*	2931
ABO	28	*KL*	9365	*SP4*	6671
CREB1	1385	*LMNA*	4000	*RIMS1*	22999
PRKAA2	5563	*PAK2*	5062	*ECE1*	1889
NFKB1	4790	*CAPN2*	824	*DRD5*	1816
IL18	3606	*SLC12A2*	6558	*BEGAIN*	57596
HNRNPAB	3182	*MADD*	8567	*RBCK1*	10616
PPP3CA	5530	*CLDN11*	5010	*RETSAT*	54884
HMOX1	3162	*PRKCG*	5582	*CAPN3*	825
GABRA1	2554	*YBX1*	4904	*SEMA6B*	10501
CCND2	894	*FBXO2*	26232	*MTR*	4548
STAT5B	6777	*SLC1A1*	6505	*SYT5*	6861
GRIK5	2901	*ITPR1*	3708	*GNB5*	10681
NGFR	4804	*SLIT2*	9353	*CASP6*	839
SLC17A6	57084	*RNPEP*	6051	*CDK4*	1019
PDGFRA	5156	*DVL1*	1855	*FZD2*	2535
ADM	133	*TNNT1*	7138	*TXNRD1*	7296
E2F1	1869	*SIPA1L1*	26037	*MTHFD1*	4522
IL5	3567	*PTAFR*	5724	*CXCR4*	7852
HIF1A	3091	*PLCB1*	23236	*STAT1*	6772
TRHDE	29953	*HMBS*	3145	*FOSL1*	8061
PRKN	5071	*BMP2*	650	*ABCA2*	20
GHSR	2693	*ADRA1B*	147	*ABCG5*	64240
CRH	1392	*ANGPT1*	284	*GSR*	2936

Supplementary Table S2 (continued)

Gene Symbol	Entrez ID	Gene Symbol	Entrez ID	Gene Symbol	Entrez ID
CNR2	1269	*NEO1*	4756	*SST*	6750
SHANK3	85358	*FBN2*	2201	*FGB*	2244
ATF2	1386	*MRAS*	22808	*ANXA5*	308
MAG	4099	*COL5A1*	1289	*NTRK1*	4914
RPS2	6187	*KCNMB1*	3779	*GATA2*	2624
SERPIND1	3053	*HNRNPK*	3190	*MXD3*	83463
CABIN1	23523	*LTC4S*	4056	*HIVEP2*	3097
AOC1	26	*JAG2*	3714	*ITGA7*	3679
FOXJ1	2302	*BRS3*	680	*CDH13*	1012
NTF3	4908	*RGS14*	10636	*QDPR*	5860
ARRB1	408	*AOX1*	316	*ATP5F1A*	498
TNNT2	7139	*HSPE1*	3336	*THRB*	7068
RTN1	6252	*PSEN2*	5664	*NFE2L2*	4780
LIMK1	3984	*ADRB2*	154	*PDE3A*	5139
NPM1	4869	*DNM1L*	10059	*CTSB*	1508
MAP2K5	5607	*PROKR1*	10887	*NUP155*	9631
HRH2	3274	*MAP2*	4133	*LONP1*	9361
PLAU	5328	*RPL6*	6128	*CES3*	23491
A2M	2	*HYAL2*	8692	*CRYAA*	1409
SNAP29	9342	*CTNNB1*	1499	*RET*	5979
HEY1	23462	*ATF4*	468	*CXCR2*	3579
NOG	9241	*PLCB4*	5332	*GSS*	2937
CCL3	6348	*ADCY8*	114	*CMA1*	1215
APOB	338	*SLK*	9748	*CALM1*	801
TRDN	10345	*NFIB*	4781	*ALB*	213
ACSM3	6296	*UCP3*	7352	*PAH*	5053
ACTB	60	*DLL1*	28514	*ADD3*	120
GDNF	2668	*MASP1*	5648	*LDHB*	3945
GAST	2520	*DGKZ*	8525	*RARG*	5916
CCNG1	900	*SNCB*	6620	*SCN4B*	6330
LEPR	3953	*PLD2*	5338	*NTN1*	9423
CD38	952	*DPP7*	29952	*SCT*	6343
FABP2	2169	*HSPD1*	3329	*MCL1*	4170
ADAM10	102	*TH*	7054	*BCL2L1*	598
EPO	2056	*TIMP2*	7077	*BSN*	8927
NQO1	1728	*VLDLR*	7436	*AIF1*	199
RELN	5649	*FGF8*	2253	*CD63*	967
IL17A	3605	*NME7*	29922	*IL10*	3586
MUC4	4585	*PPP3CB*	5532	*CD4*	920
CCNB1	891	*DDB1*	1642	*ROBO1*	6091
SNCA	6622	*AP2B1*	163	*F9*	2158
HSPA4	3308	*UBTF*	7343	*AKT3*	10000
CAND2	23066	*TIMM8B*	26521	*NRAS*	4893
GLUD1	2746	*COX5B*	1329	*PF4*	5196
SLC17A8	246213	*CTSK*	1513	*TBXAS1*	6916
SLC4A2	6522	*PGM1*	5236	*GJA1*	2697
WNT2B	7482	*ARF6*	382	*HK3*	3101
PLCD1	5333	*NRXN1*	9378	*TSC1*	7248
NOTCH2	4853	*PTPN6*	5777	*NUPR1*	26471
PRSS12	8492	*PTPRF*	5792	*DMD*	1756

Supplementary Table S2 (continued)

Gene Symbol	Entrez ID	Gene Symbol	Entrez ID	Gene Symbol	Entrez ID
LBR	3930	*FKBP1A*	2280	*HAMP*	57817
CD3D	915	*EDN2*	1907	*RRAD*	6236
HAND1	9421	*APOA4*	337	*MEN1*	4221
TLR4	7099	*GHRH*	2691	*GPR182*	11318
ACAT1	38	*APOC3*	345	*PLAT*	5327
TSHR	7253	*SPN*	6693	*ELN*	2006
MUC5AC	4586	*ACTA1*	58	*PTN*	5764
CD44	960	*ADK*	132	*SQSTM1*	8878
PRKCA	5578	*UTS2*	10911	*CCNE1*	898
CANX	821	*ARNTL2*	56938	*SYN3*	8224
CD80	941	*CACNA2D2*	9254	*MAPT*	4137
KITLG	4254	*HOXA1*	3198	*IL6R*	3570
INSL6	11172	*GPC3*	2719	*CFD*	1675
LIPE	3991	*PLCG1*	5335	*SLC11A1*	6556
IDH1	3417	*LAMP2*	3920	*TGFBR3*	7049
SELP	6403	*F2R*	2149	*ALAD*	210
MYO1C	4641	*APOA1*	335	*IL11*	3589
GAL	51083	*MUC2*	4583	*ESR1*	2099
FLT1	2321	*IRF1*	3659	*EPHX1*	2052
FGA	2243	*MMP7*	4316	*LIPA*	3988
CXCL12	6387	*ATP1A2*	477	*ADD2*	119
COL3A1	1281	*OXTR*	5021	*TREH*	11181
MT1A	4489	*LDHA*	3939	*ENSA*	2029
NES	10763	*COL18A1*	80781	*CD59*	966
MUC1	4582	*CTSL*	1514	*POLB*	5423
KLF15	28999	*IGFBP3*	3486	*PTGIS*	5740
HP	3240	*PDGFA*	5154	*FN1*	2335
MYL2	4633	*CLCNKB*	1188	*ITGB1*	3688
SLC2A1	6513	*ANXA2*	302	*FGFR4*	2264
MGP	4256	*PDE4D*	5144	*CRYBA1*	1411
CASQ2	845	*FBLN5*	10516	*AK1*	203
CBS	875	*ADA*	100	*CP*	1356
TLE5	166	*IL1RL1*	9173	*COL5A2*	1290
BDKRB2	624	*SYK*	6850	*DRD2*	1813
CALM3	808	*CCND3*	896	*SHH*	6469
DPYSL2	1808	*APOA5*	116519	*EGF*	1950
IL3	3562	*OPTN*	10133	*PODXL*	5420
CDO1	1036	*CYP2D6*	1565	*FGFR2*	2263
AQP8	343	*TGFA*	7039	*FABP1*	2168
MYH11	4629	*GAS6*	2621	*ADCY5*	111
MYOC	4653	*CNP*	1267	*BCAN*	63827
HK1	3098	*IL4*	3565	*HRG*	3273
PAX6	5080	*MSTN*	2660	*CST3*	1471
TXN2	25828	*CALM2*	805	*KLKB1*	3818
CTLA4	1493	*REN*	5972	*ALAS1*	211
HSD11B2	3291	*APOE*	348	*GPT*	2875
CD55	1604	*GLRX*	2745	*NDUFV2*	4729
PKM	5315	*PPBP*	5473	*PDK4*	5166
CRYAB	1410	*SCN2B*	6327	*ADCYAP1R1*	117
BGN	633	*ARNT2*	9915	*NCOA6*	23054

Supplementary Table S2 (continued)

Gene Symbol	Entrez ID	Gene Symbol	Entrez ID	Gene Symbol	Entrez ID
GCLC	2729	*ALDOC*	230	*DBH*	1621
IGFBP5	3488	*NUDT1*	4521	*CRP*	1401
DPP6	1804	*PDCD4*	27250	*NPTN*	27020
CREM	1390	*BIRC5*	332	*HSPA8*	3312
ATP2A3	489	*GNAS*	2778	*CEL*	1056
PTGER4	5734	*RBM10*	8241	*PICALM*	8301
STK39	27347	*NEFH*	4744	*PNOC*	5368
NR2F2	7026	*TPI1*	7167	*GSTP1*	2950
RHO	6010	*MAOA*	4128	*TCF4*	6925
CLIP2	7461	*GATA6*	2627	*STAT5A*	6776
BSG	682	*ALDH2*	217	*ASAH1*	427
TRPV1	7442	*VEGFD*	2277	*ADRA1A*	148
UCP2	7351	*C3*	718	*GIPR*	2696
PPP1CA	5499	*GJB1*	2705	*CSRP3*	8048
PROC	5624	*F2RL1*	2150	*MYLK2*	85366
SLC22A5	6584	*SHBG*	6462	*DHFR*	1719
ABCC9	10060	*FABP5*	2171	*ATP1A1*	476
PPARD	5467	*SORT1*	6272	*YWHAZ*	7534
FSHB	2488	*RGCC*	28984	*IFT172*	26160
LGALS2	3957	*YME1L1*	10730	*GOT2*	2806
DPM2	8818	*MYH6*	4624	*CHRM4*	1132
SLC18A1	6570	*BCHE*	590	*GABBR1*	2550
KCNMA1	3778	*GCLM*	2730	*KCNK10*	54207
NDUFS6	4726	*BCL2*	596	*SH2B3*	10019
MEFV	4210	*FZD9*	8326	*GC*	2638
PDHA1	5160	*PTP4A2*	8073	*YWHAH*	7533
SLC4A1	6521	*ID1*	3397	*ACAN*	176
HSPB1	3315	*PIK3CB*	5291	*ATP2C2*	9914
CYP11A1	1583	*ATP1B4*	23439	*PDGFRB*	5159
ABCC6	368	*MPP4*	58538	*ADRB1*	153
RALGAPA1	253959	*ADAM19*	8728	*CEBPD*	1052
ERAP1	51752	*GCK*	2645	*CARD9*	64170
SLC6A11	6538	*NPR1*	4881	*ZFP36*	7538
HMGCS2	3158	*ADRA2A*	150	*NDRG2*	57447
NEFM	4741	*EDNRA*	1909	*ADCY3*	109
CACNA2D1	781	*POLG*	5428	*SIRT2*	22933
ATP5MC2	517	*IRS1*	3667	*LPAR1*	1902
HCN1	348980	*HSF2*	3298	*IL23A*	51561
FGFR1	2260	*PTPN5*	84867	*BEX3*	27018
GOT1	2805	*CAPN1*	823	*C5*	727
BBC3	27113	*GRIK2*	2898	*IL1A*	3552
OXT	5020	*LTBP1*	4052	*CACNA1H*	8912
LHB	3972	*LIF*	3976	*NOP58*	51602
HNMT	3176	*PHLDA1*	22822	*KCND3*	3752
CYP2C19	1557	*NCAM1*	4684	*PAWR*	5074
SMO	6608	*FGFR3*	2261	*LDLR*	3949
ROCK2	9475	*PTGES*	9536	*THY1*	7070
ANXA3	306	*OPRK1*	4986	*KIT*	3815
COL1A1	1277	*FGF1*	2246	*HSPA2*	3306
ISL1	3670	*CHI3L1*	1116	*TACR2*	6865

Supplementary Table S2 (continued)

Gene Symbol	Entrez ID	Gene Symbol	Entrez ID	Gene Symbol	Entrez ID
EIF2S1	1965	*ACP1*	52	*GAPDH*	2597
TNFRSF1A	7132	*UMOD*	7369	*PLAUR*	5329
CASP1	834	*KCNN2*	3781	*FLT4*	2324
CD8A	925	*CDKN1A*	1026	*NME1*	4830
ACADVL	37	*HCN4*	10021	*CKM*	1158
SPP1	6696	*LIPC*	3990	*SLC4A4*	8671
ABCG8	64241	*RASA1*	5921	*CSNK2A1*	1457
BARD1	580	*IMPA1*	3612	*BRAF*	673
GRIA1	2890	*ASS1*	445	*RALBP1*	10928
APOA2	336	*MBP*	4155	*LTBP3*	4054
ENO2	2026	*TMBIM6*	7009	*KCNJ8*	3764
USH2A	7399	*PRKCH*	5583	*ITIH4*	3700
B2M	567	*PIK3R1*	5295	*ADAM2*	2515
NF1	4763	*MAF*	4094	*GJA5*	2702
AGER	177	*CSTB*	1476	*NR1D1*	9572
MAP4K3	8491	*PTPRJ*	5795	*APOC1*	341
ENTPD2	954	*ADRB3*	155	*MLYCD*	23417
FCGR3A	2214	*ABCB6*	10058	*SDHA*	6389
NT5E	4907	*SRM*	6723	*CYP1B1*	1545
GAD2	2572	*OTC*	5009	*COMT*	1312
IGFBP2	3485	*PPP1R1A*	5502	*CACNA1G*	8913
ADD1	118	*FZD3*	7976	*XYLT1*	64131
S100B	6285	*AKAP8*	10270	*SLC6A3*	6531
TRPC1	7220	*HADHA*	3030	*HDLBP*	3069
KCNN3	3782	*XRCC5*	7520	*RCAN1*	1827
GPX4	2879	*AHSG*	197	*PTGER3*	5733
TRIM50	135892	*FXYD1*	5348	*ATOX1*	475
TYMS	7298	*GFRA1*	2674	*HIP1R*	9026
SCNN1B	6338	*GNB1*	2782	*SLC6A2*	6530
CNTN5	53942	*ADNP*	23394	*XPNPEP2*	7512
NR1D2	9975	*LCAT*	3931	*NR3C2*	4306
XYLT2	64132	*ATP2B3*	492	*CACNA1C*	775
EPB41L3	23136	*TLE4*	7091	*EGR4*	1961
MDH2	4191	*NFKBIA*	4792	*GGT1*	2678
SLC19A1	6573	*KCNIP2*	30819	*CITED2*	10370
XRCC1	7515	*PSMA6*	5687	*MC4R*	4160
ENPP2	5168	*GRIA4*	2893	*HTR1A*	3350
TGFB1	7040	*COL11A1*	1301	*EIF4E*	1977
MME	4311	*HTR2A*	3356	*P2RX6*	9127
GRIN2A	2903	*SCNN1G*	6340	*HTR4*	3360
MYC	4609	*TPH2*	121278	*PRKCZ*	5590
ADRA1D	146	*AKR1A1*	10327	*CRHR1*	1394
TNFRSF8	943	*WFDC1*	58189	*F13A1*	2162
SLC9A3R2	9351	*GPX1*	2876	*ASL*	435
NUCKS1	64710	*GALNT1*	2589	*FMR1*	2332
PCSK2	5126	*FZD4*	8322	*SLC12A3*	6559
KCNIP1	30820	*GNA12*	2768	*SLC31A1*	1317
GSK3B	2932	*KCNJ11*	3767	*PPP5C*	5536
MEGF8	1954	*ABCC8*	6833	*RPS6*	6194
PI4KA	5297	*GSTM2*	2946	*TRPC5*	7224

Supplementary Table S2 (continued)

Gene Symbol	Entrez ID	Gene Symbol	Entrez ID	Gene Symbol	Entrez ID
SCGB1A1	7356	*FUCA1*	2517	*PTGDS*	5730
NTRK2	4915	*ID2*	3398	*TPM4*	7171
ADCYAP1	116	*TRIM28*	10155	*DUSP6*	1848
MFGE8	4240	*SLC24A1*	9187	*TGFB3*	7043
GSTM1	2944	*HADHB*	3032	*ADRA2C*	152
MAPK1	5594	*ADAR*	103	*DIO3*	1735
PYGB	5834	*PTPRN*	5798	*CYBA*	1535
PSMB7	5695	*DNAH10*	196385	*PTEN*	5728
ALOX5AP	241	*CACNB2*	783	*ATP1A3*	478
SLC6A1	6529	*SLC29A1*	2030	*RENBP*	5973
OCLN	100506658	*REG1A*	5967	*ID3*	3399
GNAZ	2781	*HBB*	3043	*SLC6A4*	6532
MMP14	4323	*PDE4B*	5142	*SLC22A4*	6583
SERPINI1	5274	*PPP2CA*	5515	*KCNE1*	3753
FMO3	2328	*HTR1D*	3352	*NDST1*	3340
CXCL2	2920	*THRA*	7067	*UNC5B*	219699
AKT2	208	*ENO3*	2027	*MBL2*	4153
IKBKB	3551	*GFER*	2671	*ALOX5*	240
SLC25A4	291	*ENO1*	2023	*NRP1*	8829
MYL3	4634	*GOSR2*	9570	*PKD1*	5310
PACS1	55690	*TACR3*	6870	*GPNMB*	10457
LGALS3	3958	*ARG2*	384	*KCNT1*	57582
SRD5A1	6715	*NAE1*	8883	*ACVRL1*	94
RASA2	5922	*STS*	412	*TGM2*	7052
ABCF1	23	*NOS1AP*	9722	*PTPRZ1*	5803
CFL1	1072	*SLC1A3*	6507	*RXRA*	6256
CD28	940	*IL4R*	3566	*BMPR1A*	657
TRHR	7201	*SFTPC*	6440	*PRL*	5617
AKAP12	9590	*ENTPD1*	953	*TIMP3*	7078
MEOX2	4223	*JUP*	3728	*NR1H3*	10062
RPL28	6158	*LOX*	4015	*ACACA*	31
GABRD	2563	*ROCK1*	6093	*WIPF1*	7456
G6PD	2539	*RGS2*	5997	*MTHFR*	4524
APOD	347	*RXRG*	6258	*PTPN11*	5781
PDE5A	8654	*CACNA1D*	776	*ATF3*	467
MAPK8	5599	*PRKAR2B*	5577	*HMOX2*	3163
NSMF	26012	*FOSL2*	2355	*COQ7*	10229
MFN1	55669	*OGT*	8473	*NEDD4*	4734
ATP5PF	522	*SLC11A2*	4891	*MDH1*	4190
STAR	6770	*STXBP2*	6813	*PRKAR2A*	5576
ABCC5	10057	*DLG2*	1740	*BTG1*	694
PIK3R2	5296	*DAB2*	1601	*CACNA1E*	777
AKR1B1	231	*SERPINH1*	871	*UBE2I*	7329
HNF1A	6927	*PPARA*	5465	*PCMT1*	5110
DIO2	1734	*KCNJ1*	3758	*ATP1B1*	481
LRP2	4036	*HRH3*	11255	*MARK1*	4139
BARHL1	56751	*TTR*	7276	*CDKN1B*	1027
RAD50	10111	*HTR2B*	3357	*PNMT*	5409
HRH1	3269	*CLDN5*	7122	*SOD3*	6649
CHEK2	11200	*CACNB4*	785	*HNRNPM*	4670

Supplementary Table S2 (continued)

Gene Symbol	Entrez ID	Gene Symbol	Entrez ID	Gene Symbol	Entrez ID
ABCA1	19	*YY1*	7528	*MTOR*	2475
SLC8A3	6547	*LTBP2*	4053	*VDAC1*	7416
SLC2A3	6515	*WT1*	7490	*KCNJ10*	3766
SLC40A1	30061	*ILK*	3611	*HPGD*	3248
PTHLH	5744	*LEF1*	51176	*FKBP1B*	2281
PKLR	5313	*SLC22A1*	6580	*SCARB1*	949
MYH9	4627	*TNFRSF4*	7293	*GGT7*	2686
HSD17B4	3295	*GJA4*	2701	*SLC17A7*	57030
SLC4A3	6508	*FABP3*	2170	*TFRC*	7037
KCNH2	3757	*CDK6*	1021	*ITPR2*	3709
CAMK2D	817	*P2RY1*	5028	*TYRO3*	7301
SND1	27044	*SNAP25*	6616	*PEBP1*	5037
TNNI3	7137	*HSPB2*	3316	*GABRA6*	2559
FGD4	121512	*CNTF*	1270	*ACHE*	43
IGFALS	3483	*RAF1*	5894	*ARNTL*	406
PLCB3	5331	*KLRK1*	22914	*PDE1A*	5136
TAP1	6890	*FOXO1*	2308	*ACADS*	35
TSC2	7249	*SULT1A1*	6817	*PDX1*	3651
CYP11B2	1585	*PLA2G4A*	5321	*MVP*	9961
CDH2	1000	*VEGFC*	7424	*EPOR*	2057
UFD1	7353	*PDGFB*	5155	*YWHAB*	7529
ACP2	53	*VIM*	7431	*ACE*	1636
ATP7A	538	*CALR*	811	*PSMA2*	5683
GALR1	2587	*PCNA*	5111	*PRKCE*	5581
AQP4	361	*EDNRB*	1910	*OPN1SW*	611
MERTK	10461	*SLIT3*	6586	*SIX1*	6495
TBXA2R	6915	*RGS7*	6000	*GTF2IRD1*	9569
TRH	7200	*EGLN1*	54583	*PSMC3*	5702
PRKCD	5580	*PDPK1*	5170	*P4HB*	5034
CACNG4	27092	*KYNU*	8942	*RAMP2*	10266
CYTH1	9267	*STAT3*	6774	*HSD11B1*	3290
OLFM1	10439	*TPH1*	7166	*PSMB1*	5689
ALDH1A2	8854	*APAF1*	317	*BECN1*	8678
CROT	54677	*DPYSL5*	56896	*DDR1*	780
MAPK9	5601	*DUSP1*	1843	*PTGS1*	5742
EGR1	1958	*SLC26A4*	5172	*DCC*	1630
GLP1R	2740	*DGKB*	1607	*F3*	2152
IREB2	3658	*TGFBR1*	7046	*PPFIA4*	8497
SELE	6401	*GBP2*	2634	*DLAT*	1737
MSX2	4488	*NOX4*	50507	*FEZ2*	9637
CNR1	1268	*VTN*	7448	*PYGM*	5837
MSI1	4440	*ASIC1*	41	*TMED2*	10959
SLC34A1	6569	*BAD*	572	*ASCL1*	429
NR3C1	2908	*TPM1*	7168	*TTPA*	7274
MAP2K6	5608	*ACO1*	48	*OGA*	10724
ATP2A1	487	*IL15*	3600	*CDKN2A*	1029
MYBPH	4608	*PFKFB3*	5209	*CEBPB*	1051
TMPO	7112	*AGTR1*	185	*TFPI2*	7980
TCEA2	6919	*MCAM*	4162	*JUN*	3725
PPP1CC	5501	*OBSCN*	84033	*CSF1R*	1436

Supplementary Table S2 (continued)

Gene Symbol	Entrez ID	Gene Symbol	Entrez ID	Gene Symbol	Entrez ID
ATP2A2	488	*DAB2IP*	153090	*SERPINE1*	5054
NF2	4771	*CACNA1A*	773	*CAP2*	10486
CAST	831	*ALDH3A1*	218	*TIMM44*	10469
FBXO32	114907	*DDIT3*	1649	*NOX1*	27035
AR	367	*GRIN1*	2902	*TGFBR2*	7048
SIK1	150094	*CALU*	813	*SARDH*	1757
GATM	2628	*IFNG*	3458	*CRK*	1398
NOTCH1	4851	*FOS*	2353	*HSPA1B*	3304
SNAP91	9892	*HSPA5*	3309	*SCNN1A*	6337
G6PC3	92579	*EMD*	2010	*KCNB1*	3745
SFRP1	6422	*JUNB*	3726	*KCNE2*	9992
MAT2A	4144	*SLC12A1*	6557	*P2RX4*	5025
TRPC3	7222	*XPO1*	7514	*AKT1*	207
CLIC6	54102	*GNAQ*	2776	*FAT1*	2195
SCN5A	6331	*CAMK2A*	815	*PPM1F*	9647
SCN9A	6335	*AIFM1*	9131	*PCSK5*	5125
APP	351	*CLU*	1191	*CCN3*	4856
GATA4	2626	*AP3M1*	26985	*HPX*	3263
VDAC2	7417	*HK2*	3099	*LRP4*	4038
ATP2B1	490	*OPRD1*	4985	*SLC5A2*	6524
VHL	7428	*CX3CL1*	6376	*MIF*	4282
PRSS1	5644	*NR5A2*	2494	*MAP2K1*	5604
CBR1	873	*RACK1*	10399	*RUFY1*	80230
CTSC	1075	*DYNLRB1*	83658	*CIDEA*	1149
COL9A1	1297	*ABAT*	18	*PDIA2*	64714
TNFRSF1B	7133	*APLP2*	334	*CHAT*	1103
CASP7	840	*SLC9A1*	6548	*EXOG*	9941
ZEB1	6935	*ACAA1*	30	*LIG4*	3981
SGCB	6443	*ETS1*	2113	*DIS3*	22894
ACTG1	71	*APCS*	325	*ARHGEF2*	9181
DSTN	11034	*PSMB5*	5693	*GDF1*	2657
WNT6	7475	*SORD*	6652	*SLC39A13*	91252
LPIN2	9663	*SDF4*	51150	*TNFRSF17*	608
POLRMT	5442	*FAH*	2184	*UBE4B*	10277
NRF1	4899	*MYH7*	4625	*UST*	10090
RASGRP3	25780	*CDK5R1*	8851	*MBD2*	8932
BMPR2	659	*DBN1*	1627	*LRP6*	4040
RBP4	5950	*SLC2A4*	6517	*MRPS6*	64968
S100A4	6275	*ACTC1*	70	*PPP2R1B*	5519
IGF2	3481	*DDC*	1644	*XIRP1*	165904
CUL5	8065	*NOS3*	4846	*LACTB*	114294
ADAMTS1	9510	*NPR2*	4882	*DGUOK*	1716
PPM1B	5495	*PTPRA*	5786	*ATG7*	10533
GUCY1A2	2977	*CEBPA*	1050	*MED13*	9969
ADGRL1	22859	*USF1*	7391	*HDAC2*	3066
SP1	6667	*CHRM2*	1129	*MUC17*	140453
CACNG8	59283	*NR1H4*	9971	*TNFRSF9*	3604
SURF1	6834	*PPAT*	5471	*LRRTM3*	347731
HCN2	610	*OPRM1*	4988	*DPH7*	92715
UCP1	7350	*ACVR2B*	93	*DSCAML1*	57453

Supplementary Table S2 (continued)

Gene Symbol	Entrez ID	Gene Symbol	Entrez ID	Gene Symbol	Entrez ID
KAT6A	7994	*SLN*	6588	*FKRP*	79147
DDX31	64794	*REEP1*	65055	*SIRT7*	51547
PRTN3	5657	*HAVCR2*	84868	*MED13L*	23389
PFAS	5198	*PLEKHA3*	65977	*KAT2B*	8850
FAM135A	57579	*AMOTL1*	154810	*SLC7A4*	6545
ABCF2	10061	*SNAPC5*	10302	*TAF10*	6881
EZH2	2146	*POSTN*	10631	*NDUFB5*	4711
CARD10	29775	*EIF4G2*	1982	*PTPRB*	5787
IL21R	50615	*IRAK4*	51135	*BAZ1B*	9031
RHOC	389	*MAP4K4*	9448	*KRIT1*	889
SYNE2	23224	*MDM2*	4193	*ENTR1*	10807
MOSPD3	64598	*GSC2*	2928	*NCF4*	4689
PARD6G	84552	*DNAH9*	1770	*MYBPC3*	4607
HEYL	26508	*HIC2*	23119	*TDRD6*	221400
NET1	10276	*ARHGAP9*	64333	*ATAD3A*	55210
SMAD6	4091	*EMILIN2*	84034	*CD68*	968
RHBDF1	64285	*COL4A4*	1286	*CERS1*	10715
DSE	29940	*CLCN6*	1185	*MBD1*	4152
LTF	4057	*ARHGDIB*	397	*PROCR*	10544
MAP4K1	11184	*IDH2*	3418	*NODAL*	4838
PRPF8	10594	*UCHL5*	51377	*CSRNP1*	64651
MKI67	4288	*BEST3*	144453	*SCARF2*	91179
PMS2	5395	*EIF4G1*	1981	*ZFPM2*	23414
ACSS2	55902	*ZNF366*	167465	*NAGA*	4668
RNF2	6045	*STK32B*	55351	*YAP1*	10413
COTL1	23406	*CDK15*	65061	*FHOD3*	80206
DNPEP	23549	*ARFGAP2*	84364	*SMOX*	54498
MAML2	84441	*PARL*	55486	*ADAMTS10*	81794
WDR36	134430	*BAZ1A*	11177	*TLL1*	7092
ANAPC2	29882	*IGFBP7*	3490	*LSP1*	4046
SATB1	6304	*TAF1C*	9013	*SMARCAL1*	50485
UPP1	7378	*FBXW5*	54461	*SOCS4*	122809
PAX2	5076	*UNC45B*	146862	*KLHDC2*	23588
MCM2	4171	*VSX1*	30813	*ZNF408*	79797
ITGB2	3689	*LRCH1*	23143	*PKP2*	5318
GBA2	57704	*ADTRP*	84830	*MYEF2*	50804
RSPH14	27156	*TBX5*	6910	*DOCK1*	1793
SMARCE1	6605	*ARMH3*	79591	*SLC2A10*	81031
TBK1	29110	*LRP8*	7804	*HDHD5*	27440
CTDSP1	58190	*TIA1*	7072	*IL17RA*	23765
STN1	79991	*STAT4*	6775	*REM1*	28954
CTNND1	1500	*TYMP*	1890	*TTLL4*	9654
TCTN3	26123	*XPC*	7508	*CA12*	771
GTF3C5	9328	*ELMO2*	63916	*MAP2K3*	5606
SPTA1	6708	*SCMH1*	22955	*MYRF*	745
JPH2	57158	*WDR1*	9948	*SGCD*	6444
MEIS2	4212	*DSP*	1832	*MMP27*	64066
BAG1	573	*ATR*	545	*DOT1L*	84444
SIRT6	51548	*POMP*	51371	*ARVCF*	421
SLC66A2	80148	*ABCG4*	64137	*FIS1*	51024

Supplementary Table S2 (continued)

Gene Symbol	Entrez ID	Gene Symbol	Entrez ID	Gene Symbol	Entrez ID
NOXA1	10811	*STIM1*	6786	*ARIH1*	25820
FZD7	8324	*SLC22A13*	9390	*SHOC2*	8036
REXO4	57109	*RETREG2*	79137	*NDUFB8*	4714
VCPKMT	79609	*IL18RAP*	8807	*BBS4*	585
SETBP1	26040	*TRAF6*	7189	*NMNAT3*	349565
VAV2	7410	*CD34*	947	*CCNL2*	81669
RAPSN	5913	*PROX1*	5629	*ANKS1A*	23294
TJP1	7082	*NEK8*	284086	*GP1BA*	2811
WNT8A	7478	*SUMO1*	7341	*MRPL41*	64975
RPL3L	6123	*AEBP1*	165	*SSNA1*	8636
TFAP2B	7021	*SLC30A5*	64924	*TFAP2A*	7020
TADA3	10474	*ODAD2*	55130	*SENP1*	29843
SP140	11262	*NISCH*	11188	*LIPT1*	51601
DGCR8	54487	*CFAP298*	56683	*C2CD2L*	9854
HERC1	8925	*PPP2R3A*	5523	*EHF*	26298
NOD2	64127	*AKIP1*	56672	*NCOR2*	9612
AGL	178	*USP37*	57695	*GYPC*	2995
KDM4A	9682	*PTOV1*	53635	*PIN1*	5300
BCL3	602	*CFL2*	1073	*FOXP4*	116113
CAB39	51719	*BCL2L13*	23786	*PPP6R1*	22870
IL17RC	84818	*ERCC1*	2067	*FANCL*	55120
MDN1	23195	*RAD18*	56852	*LRBA*	987
EPN3	55040	*THSD1*	55901	*CNNM2*	54805
CCT7	10574	*PLEKHM2*	23207	*CYP2C9*	1559
CA9	768	*WNT10A*	80326	*TCF7L2*	6934
MAP3K5	4217	*NEURL1*	9148	*KIFBP*	26128
MST1R	4486	*LTBP4*	8425	*MAML3*	55534
PIK3CG	5294	*MAP2K4*	6416	*PITPNM3*	83394
SLC22A14	9389	*VIL1*	7429	*KLHDC1*	122773
RNF146	81847	*SNTA1*	6640	*CHST12*	55501
ABCA3	21	*NTNG2*	84628	*ESS2*	8220
DPT	1805	*OLIG2*	10215	*ERO1A*	30001
NFKB2	4791	*EYA4*	2070	*ZFHX3*	463
MIB1	57534	*NDUFS2*	4720	*FOXO3*	2309
MPZL2	10205	*ANTXR1*	84168	*PHC1*	1911
EPHA2	1969	*COL4A1*	1282	*CD3E*	916
RNF44	22838	*COLEC10*	10584	*AGPAT2*	10555
ANKIB1	54467	*ZNF469*	84627	*CALR3*	125972
RALY	22913	*SP110*	3431	*DNAAF2*	55172
LMOD1	25802	*DUSP29*	338599	*ISG15*	9636
ARHGAP22	58504	*MFF*	56947	*NIF3L1*	60491
SERPINF2	5345	*SALL3*	27164	*ITGA3*	3675
PLTP	5360	*RSPH3*	83861	*LRMDA*	83938
TMEM237	65062	*MCM10*	55388	*EIF2AK4*	440275
ADAMTS7	11173	*LTA4H*	4048	*ARHGAP10*	79658
GLMN	11146	*PHPT1*	29085	*RRAS*	6237
LRIG1	26018	*AASDHPPT*	60496	*CLK1*	1195
DNA2	1763	*PTPN3*	5774	*CDKN1C*	1028
FERMT1	55612	*ACTN2*	88	*CNOT9*	9125
CARF	79800	*GNA14*	9630	*CACFD1*	11094

Supplementary Table S2 (continued)

Gene Symbol	Entrez ID	Gene Symbol	Entrez ID	Gene Symbol	Entrez ID
RAG1	5896	*SRF*	6722	*RCBTB1*	55213
SLC39A8	64116	*ADAMTS2*	9509	*NDOR1*	27158
RORC	6097	*ARID1A*	8289	*WRN*	7486
MYL7	58498	*CLIC3*	9022	*PRDM5*	11107
NSUN5	55695	*MANF*	7873	*MOCOS*	55034
NACC2	138151	*ZEB2*	9839	*TOP3B*	8940
PEX10	5192	*SACS*	26278	*SESN3*	143686
MRPL20	55052	*BRD4*	23476	*GLB1L*	79411
SLC39A12	221074	*EBAG9*	9166	*PPIL2*	23759
MDM4	4194	*RYR1*	6261	*SOX4*	6659
IRX4	50805	*NDRG1*	10397	*EFNB2*	1948
SLC2A6	11182	*SAMHD1*	25939	*NECTIN2*	5819
ALS2	57679	*SIRT3*	23410	*GOPC*	57120
RANGAP1	5905	*RAPH1*	65059	*KCNG2*	26251
CD163	9332	*MTTP*	4547	*TAB2*	23118
ANKRD23	200539	*TM6SF2*	53345	*ITGAV*	3685
HNRNPA2B1	3181	*ADGRV1*	84059	*MKRN2*	23609
SIRT4	23409	*HOXD13*	3239	*STOX1*	219736
CPEB1	64506	*TBX20*	57057	*APOH*	350
YES1	7525	*NDUFS3*	4722	*TNFRSF11A*	8792
ERCC6	2074	*CRELD1*	78987	*UAP1L1*	91373
KAT8	84148	*NUP205*	23165	*ATG16L1*	55054
EIF3E	3646	*KDM2A*	22992	*MOV10*	4343
XPO5	57510	*NCF2*	4688	*XYLB*	9942
TBCA	6902	*DSC2*	1824	*SURF6*	6838
AMFR	267	*MAP3K7*	6885	*MBD3*	53615
H2AC17	8336	*ABCA4*	24	*SERPINC1*	462
RNF25	64320	*STK38*	11329	*RBM20*	282996
TRIP11	9321	*SARS2*	54938	*ARID4A*	5926
UCK1	83549	*DDX58*	23586	*MYPN*	84665
NEBL	10529	*TREM1*	54210	*EFEMP1*	2202
BAIAP3	8938	*RANBP1*	5902	*CRKL*	1399
C8A	731	*RHOJ*	57381	*SIRT1*	23411
ADAMTS19	171019	*CRBN*	51185	*CRYGN*	155051
UBE2L3	7332	*RBPJ*	3516	*KCNG4*	93107
SSR2	6746	*SLC24A5*	283652	*CCR6*	1235
LRP5	4041	*DGCR2*	9993	*B3GALT6*	126792
MMP21	118856	*MYOT*	9499	*VANGL1*	81839
FIP1L1	81608	*PRKACB*	5567	*AOPEP*	84909
MAPK11	5600	*DKK1*	22943	*EMILIN3*	90187
SEMA3C	10512	*STK36*	27148	*KCTD3*	51133
HIPK2	28996	*ADAMTS12*	81792	*FLT3*	2322
ANKS6	203286	*CFAP97*	57587	*BNC2*	54796
COL8A1	1295	*PACSIN3*	29763	*KDM1B*	221656
F11	2160	*NMNAT2*	23057	*SPAG1*	6674
COG5	10466	*NHLRC2*	374354	*CIC*	23152
DICER1	23405	*STKLD1*	169436	*ACAP3*	116983
DGCR6	8214	*CDH5*	1003	*BICC1*	80114
IRF5	3663	*RAB25*	57111	*ARID1B*	57492
TRAF2	7186	*SGO2*	151246	*BRPF1*	7862

Supplementary Table S2 (continued)

Gene Symbol	Entrez ID	Gene Symbol	Entrez ID	Gene Symbol	Entrez ID
RAD51	5888	*RRAS2*	22800	*LIG3*	3980
MAD2L2	10459	*PLXND1*	23129	*SORL1*	6653
NDUFC2	4718	*DOLK*	22845	*MEF2A*	4205
SHMT2	6472	*EIF4EBP2*	1979	*SOS1*	6654
IL18R1	8809	*DUSP10*	11221	*EPRS1*	2058
NPC1	4864	*CAMSAP1*	157922	*CLASP1*	23332
MAML1	9794	*EHMT1*	79813	*THBS2*	7058
ANK1	286	*RABL6*	55684	*CYB561*	1534
GYS1	2997	*FCGR2A*	2212	*IRF2BPL*	64207
ZNF142	7701	*RAPGEF6*	51735	*GMDS*	2762
DNAL1	83544	*SYTL2*	54843	*RECQL4*	9401
ZNF236	7776	*ABCB8*	11194	*TBX1*	6899
P4HA2	8974	*MMP20*	9313	*PAPPA*	5069
COLEC11	78989	*SPRED1*	161742	*NSD1*	64324
DUSP16	80824	*LOXL1*	4016	*SPHK2*	56848
PLA2G7	7941	*IRF9*	10379	*NEMF*	9147
NDUFB3	4709	*IL7R*	3575	*ELK3*	2004
LIPG	9388	*EED*	8726	*RFX3*	5991
NFATC4	4776	*CRTC1*	23373	*BAG3*	9531
AGAP3	116988	*HDAC1*	3065	*IRAK3*	11213
NLK	51701	*RNLS*	55328	*USP3*	9960
ENDOG	2021	*CTNNA3*	29119	*TRNT1*	51095
DSC1	1823	*DMPK*	1760	*BLK*	640
TGFBRAP1	9392	*MLLT10*	8028	*GPATCH2*	55105
LRP1	4035	*DIABLO*	56616	*ZDHHC8*	29801
QSOX2	169714	*PTGIR*	5739	*LAMA2*	3908
FOXE3	2301	*KBTBD3*	143879	*HTRA2*	27429
PSTPIP1	9051	*TARDBP*	23435	*DIXDC1*	85458
RCC1L	81554	*MYLK*	4638	*FCGR1A*	2209
MED15	51586	*XPO7*	23039	*TRPC4AP*	26133
HDAC4	9759	*MYOZ2*	51778	*SALL1*	6299
RAC2	5880	*SLMAP*	7871	*TNNC1*	7134
RPL7A	6130	*FAM117B*	150864	*BCL11A*	53335
NACA	4666	*CTSG*	1511	*DSG1*	1828
STK11	6794	*CAVIN1*	284119	*LY86*	9450
LAMC1	3915	*RYR2*	6262	*ALG2*	85365
PROZ	8858	*NFATC2*	4773	*CRB1*	23418
LINGO1	84894	*DZIP1*	22873	*PAOX*	196743
FOXP1	27086	*EYA1*	2138	*PON2*	5445
FKBP8	23770	*RSPH1*	89765	*ARRDC1*	92714
MTRR	4552	*CD3G*	917	*CBFB*	865
HSD3B2	3284	*ADAMTS17*	170691	*PIWIL4*	143689
NFATC3	4775	*FLNC*	2318	*GNB1L*	54584
PRKAG3	53632	*BTG4*	54766	*ME3*	10873
COL6A3	1293	*MKKS*	8195	*ANK2*	287
POLR1C	9533	*USP34*	9736	*GATB*	5188
CBLN2	147381	*DNAJB2*	3300	*PREX2*	80243
CD209	30835	*IRX5*	10265	*TAX1BP3*	30851
DVL2	1856	*CHMP4B*	128866	*BRD3*	8019
FOXR1	283150	*RARRES2*	5919	*FKBP6*	8468

Supplementary Table S2 (continued)

Gene Symbol	Entrez ID	Gene Symbol	Entrez ID	Gene Symbol	Entrez ID
MMP15	4324	*BCR*	613	*RIT1*	6016
MCOLN1	57192	*MYH7B*	57644	*RANBP10*	57610
LMCD1	29995	*DPF3*	8110	*COLGALT1*	79709
LMAN2	10960	*PRMT5*	10419	*UBR7*	55148
PRG4	10216	*CYLD*	1540	*IL22*	50616
GSTA4	2941	*TBX4*	9496	*MT-ND4*	4538
HNRNPC	3183	*STAT6*	6778	*CYP2E1*	1571
NAV2	89797	*C8G*	733	*KCNE5*	23630
SUPT16H	11198	*SLC35E2A*	9906	*DUSP5*	1847
TSPAN12	23554	*H2AC16*	8332	*TNXB*	7148
TTC39A	22996	*COL22A1*	169044	*F7*	2155
PRKG1	5592	*LRRC32*	2615	*GGTLC2*	91227
DSG2	1829	*CBFA2T2*	9139	*EPHA4*	2043
ADIPOR2	79602	*C1R*	715	*CCR3*	1232
TAPBPL	55080	*BCL9L*	283149	*NECAB2*	54550
MMP1	4312	*LZTR1*	8216	*ZBTB46*	140685
MFAP5	8076	*CEP68*	23177	*PTPRH*	5794
C7	730	*ERCC2*	2068	*SFRP2*	6423
CLDN4	1364	*MRPS2*	51116	*TNFAIP6*	7130
JRKL	8690	*TNFRSF14*	8764	*MIR208A*	406990
DDX10	1662	*NECAB3*	63941	*UPF3B*	65109
S100A5	6276	*SNX25*	83891	*JCAD*	57608
NTS	4922	*LAMA4*	3910	*MIR92A1*	407048
SOX6	55553	*DDB2*	1643	*DCN*	1634
MEX3C	51320	*CDK13*	8621	*TSFM*	10102
DIP2A	23181	*TRMU*	55687	*SERPINA5*	5104
PTCH1	5727	*ZNF174*	7727	*ALPL*	249
SNAPC4	6621	*MYL9*	10398	*PIGM*	93183
ADAMTS16	170690	*TTLL3*	26140	*GNB3*	2784
NDFIP1	80762	*TRAPPC10*	7109	*S1PR1*	1901
CERS2	29956	*FOXH1*	8928	*BID*	637
SDF2L1	23753	*RAB39A*	54734	*ITLN1*	55600
CWF19L2	143884	*SPAG9*	9043	*PTK2B*	2185
FKTN	2218	*VPS11*	55823	*MYOM1*	8736
ARPC4	10093	*TP53BP1*	7158	*CFHR1*	3078
CCNT1	904	*TNFRSF10A*	8797	*FCGR2B*	2213
IL33	90865	*IRF4*	3662	*MXRA8*	54587
BRF1	2972	*WNT3A*	89780	*MT-TA*	4553
ALOX12	239	*THAP7*	80764	*NFATC1*	4772
SH3PXD2A	9644	*SGCA*	6442	*FAS*	355
ITGA9	3680	*CCM2*	83605	*KIF11*	3832
LYPLAL1	127018	*ZNF346*	23567	*KCTD18*	130535
SURF4	6836	*EDF1*	8721	*SOS2*	6655
EMILIN1	11117	*COL4A2*	1284	*FOLR1*	2348
IL36RN	26525	*MIR200B*	406984	*HIRA*	7290
VCL	7414	*PRODH*	5625	*SLC6A18*	348932
EMC8	10328	*MT-ND3*	4537	*FTL*	2512
ELANE	1991	*CORIN*	10699	*SCNN1D*	6339
SELPLG	6404	*CXCL1*	2919	*GSN*	2934
PTPN22	26191	*MRPL40*	64976	*MPO*	4353

Supplementary Table S2 (continued)

Gene Symbol	Entrez ID	Gene Symbol	Entrez ID	Gene Symbol	Entrez ID
ADAMTS4	9507	*DNAI1*	27019	*MLXIPL*	51085
DDAH2	23564	*MIR186*	406962	*QRSL1*	55278
KDM1A	23028	*MIR204*	406987	*TNFSF9*	8744
PLCD4	84812	*SHC1*	6464	*FLACC1*	130540
PALD1	27143	*PIK3R5*	23533	*KIF21B*	23046
GPR149	344758	*MT-ND4L*	4539	*ALMS1*	7840
PRKAG2	51422	*ULK4*	54986	*ADAMTS13*	11093
H2AX	3014	*SIK2*	23235	*SIN3A*	25942
RAB7B	338382	*BAMBI*	25805	*FURIN*	5045
ICA1L	130026	*KCNE3*	10008	*STAT2*	6773
CECR3	27442	*ATAD3B*	83858	*ABHD11-AS1*	171022
SF1	7536	*MT-TP*	4571	*TF*	7018
GIMAP2	26157	*EPG5*	57724	*ELAC2*	60528
ACTL10	170487	*MIR26A1*	407015	*UQCRC1*	7384
CEP85L	387119	*PAEP*	5047	*GNA11*	2767
CDK9	1025	*MIR134*	406924	*FUT4*	2526
NME2	4831	*HNRNPH1*	3187	*SLC9A3R1*	9368
MT-TF	4558	*PPP1R1B*	84152	*SCN11A*	11280
KIF6	221458	*SNAI1*	6615	*FHL1*	2273
UBAC1	10422	*IER3*	8870	*WNK1*	65125
BCORL1	63035	*KMT2C*	58508	*HAS1*	3036
PLCH2	9651	*IKBKG*	8517	*S1PR2*	9294
ZBTB18	10472	*AKR1C4*	1109	*PDYN*	5173
CYP27A1	1593	*FDX1*	2230	*VAV3*	10451
PRSS23	11098	*IL13RA1*	3597	*RASSF1*	11186
CD46	4179	*CSNK2B*	1460	*UBE2J2*	118424
C1QB	713	*MSR1*	4481	*MIR133A2*	406923
TSPAN17	26262	*ATXN2*	6311	*PDCD1*	5133
MIR361	494323	*NPY1R*	4886	*TLNRD1*	59274
RAB12	201475	*BCS1L*	617	*F13B*	2165
SUFU	51684	*ACE2*	59272	*FKBP14*	55033
TLR9	54106	*RPL17*	6139	*SOX18*	54345
HLA-DMA	3108	*TXNL4A*	10907	*ZNF532*	55205
CACYBP	27101	*PRSS57*	400668	*KLF4*	9314
GALNT11	63917	*ZNF280A*	129025	*MIR223*	407008
PIP5KL1	138429	*CHD8*	57680	*PIGL*	9487
MIR381	494330	*STK16*	8576	*SRSF6*	6431
PRICKLE1	144165	*SLC22A2*	6582	*FXYD2*	486
ZNF341	84905	*SERPINF1*	5176	*PPARGC1A*	10891
IDO1	3620	*TEK*	7010	*MIR125B2*	406912
CALML6	163688	*MIR28*	407020	*CELSR3*	1951
DAAM1	23002	*SELL*	6402	*RAB5A*	5868
RN7SL2	378706	*PCDHA13*	56136	*PPID*	5481
PNKD	25953	*IFT74*	80173	*UTS2R*	2837
PMPCA	23203	*RBMS3*	27303	*INPP5E*	56623
GAS5	60674	*DGCR6L*	85359	*GDF15*	9518
PAK1	5058	*CIRBP*	1153	*PON3*	5446
SIDT1	54847	*CDC42SE2*	56990	*UNC5A*	90249
CCNO	10309	*EIF2S2*	8894	*FADS1*	3992
MT-TG	4563	*DNAAF4*	161582	*CFAP53*	220136

Supplementary Table S2 (continued)

Gene Symbol	Entrez ID	Gene Symbol	Entrez ID	Gene Symbol	Entrez ID
MIR130B	406920	*FCAR*	2204	*RNF113A*	7737
MRGPRD	116512	*MTMR2*	8898	*HDAC8*	55869
SERPING1	710	*SMARCD3*	6604	*SOX15*	6665
BUD23	114049	*IQGAP1*	8826	*COX15*	1355
OBP2A	29991	*TRPM2*	7226	*MC1R*	4157
GFI1B	8328	*RBFA*	79863	*AKT1S1*	84335
WWP1	11059	*CHD7*	55636	*SURF2*	6835
POU2AF1	5450	*CPEB4*	80315	*C2CD6*	151254
CTDP1	9150	*SKI*	6497	*MYOF*	26509
DDX41	51428	*F10*	2159	*CASP4*	837
MS4A6A	64231	*ACSL1*	2180	*SERPINA3*	12
TPCN2	219931	*ABL1*	25	*NDP*	4693
MIR183	406959	*SLC6A8*	6535	*DTNA*	1837
ALG9	79796	*NCL*	4691	*LCN8*	138307
ESRRB	2103	*MT-CO1*	4512	*LINC02907*	157927
KLRC4	8302	*DAG1*	1605	*VEGFB*	7423
JAGN1	84522	*TSPAN2*	10100	*NSUN6*	221078
PROS1	5627	*MYOCD*	93649	*MYCN*	4613
EPN1	29924	*RERE*	473	*IL1RN*	3557
NPHP3	27031	*MIPEP*	4285	*ADIPOR1*	51094
MBD5	55777	*RAB24*	53917	*TFPI*	7035
USP19	10869	*PLA2G15*	23659	*TNFRSF13B*	23495
ARL2BP	23568	*LUM*	4060	*RPS27L*	51065
GLA	2717	*ALG10B*	144245	*CEP170B*	283638
TMED9	54732	*ANGPTL4*	51129	*PSME4*	23198
AIMP1	9255	*BAP1*	8314	*PARP1*	142
NR1I3	9970	*NPAT*	4863	*MYD88*	4615
OPA1	4976	*MUS81*	80198	*PML*	5371
MMP23B	8510	*HLX*	3142	*TNFSF4*	7292
TUBA4A	7277	*POLE2*	5427	*MIR375*	494324
CCL26	10344	*B3GAT3*	26229	*CXCL8*	3576
LCN1	3933	*SLC27A6*	28965	*FANCD2*	2177
FERMT2	10979	*RGMA*	56963	*HSPB6*	126393
HSPA9	3313	*ERF*	2077	*KIF7*	374654
DOK3	79930	*MYH4*	4622	*MMP13*	4322
ORC2	4999	*TREX1*	11277	*EPDR1*	54749
NRIP1	8204	*TOMM70*	9868	*SMAD3*	4088
SLC23A1	9963	*PLG*	5340	*ATXN1*	6310
PPP1R15A	23645	*MIR27B*	407019	*APH1B*	83464
MIR142	406934	*NONO*	4841	*F5*	2153
RIPK3	11035	*ITCH*	83737	*SLC35F2*	54733
LAT2	7462	*HRC*	3270	*PRKACA*	5566
ALDH6A1	4329	*FNDC3B*	64778	*TRPV3*	162514
TNF	7124	*NAIP*	4671	*ICOS*	29851
NEDD4L	23327	*CAV2*	858	*GSTA5*	221357
CAV3	859	*TOMM40*	10452	*ABCC1*	4363
BVES	11149	*COL5A1-AS1*	414316	*FGF21*	26291
PARK7	11315	*MT-TR*	4573	*KLF13*	51621
GLI1	2735	*SPI1*	6688	*NOD1*	10392
NEFL	4747	*MT-TS2*	4575	*ARCN1*	372

Supplementary Table S2 (continued)

Gene Symbol	Entrez ID	Gene Symbol	Entrez ID	Gene Symbol	Entrez ID
NPC1L1	29881	*EIF2AK2*	5610	*SGO1*	151648
CBL	867	*NUTF2*	10204	*EDN3*	1908
FADD	8772	*MMP3*	4314	*SMAD7*	4092
ELOVL6	79071	*PKD1L2*	114780	*RAB1B*	81876
TNNI3K	51086	*CRYBA2*	1412	*KCNK3*	3777
PRR7	80758	*MIR34A*	407040	*GRPEL1*	80273
RLN3	117579	*ZNF516*	9658	*GRK1*	6011
GRN	2896	*MAP2K2*	5605	*BMERB1*	89927
CYP51A1	1595	*PCDHA9*	9752	*TIAL1*	7073
CXCR5	643	*CNPPD1*	27013	*H1-4*	3008
SAPCD2	89958	*SLC30A4*	7782	*CETP*	1071
ADGRE2	30817	*UBQLN4*	56893	*EPCAM*	4072
SDHD	6392	*TNFSF10*	8743	*TYR*	7299
TRIM63	84676	*TAS1R3*	83756	*ATP6AP2*	10159
MIR27A	407018	*ATP5F1B*	506	*CTRL*	1506
MICA	100507436	*TLR6*	10333	*EEF2*	1938
LCN9	392399	*F12*	2161	*PTPN1*	5770
GCH1	2643	*LTA*	4049	*AKAP1*	8165
AREG	374	*H3C1*	8350	*PSORS1C1*	170679
MIR215	406997	*TUBB1*	81027	*LPA*	4018
IL10RB	3588	*XXYLT1*	152002	*GIMAP7*	168537
SLC23A3	151295	*GP1BB*	2812	*TBR1*	10716
MT-TN	4570	*PUS3*	83480	*SMARCA2*	6595
SLC25A1	6576	*SERPINA11*	256394	*F11R*	50848
DDAH1	23576	*MIR137*	406928	*DNAJC30*	84277
SUN1	23353	*KLF5*	688	*ACTA2*	59
MBL1P	8512	*ROBO4*	54538	*ANKRD1*	27063
DNAH1	25981	*THBS1*	7057	*FXYD3*	5349
MIR146A	406938	*TBX3*	6926	*DNAH5*	1767
SCO1	6341	*DAXX*	1616	*CSF2RB*	1439
PDLIM5	10611	*SNCG*	6623	*MIR152*	406943
ASXL3	80816	*ADAMTSL2*	9719	*PECAM1*	5175
JAML	120425	*PTX3*	5806	*CCR1*	1230
PNKP	11284	*RN7SL3*	378707	*ITGA2B*	3674
GK	2710	*LEMD3*	23592	*ELK1*	2002
ALPK3	57538	*F8*	2157	*SLC8B1*	80024
GIMAP8	155038	*TNFRSF12A*	51330	*AXIN2*	8313
CTDSPL	10217	*TXNIP*	10628	*TAF1*	6872
C11orf1	64776	*IL27*	246778	*LCN12*	286256
HFE	3077	*TLR3*	7098	*AK8*	158067
AKAP5	9495	*MT-TE*	4556	*ANGPTL7*	10218
RSAD2	91543	*ITGAM*	3684	*KRAS*	3845
SRGAP3	9901	*RPSA*	3921	*CTF1*	1489
MEST	4232	*MRE11*	4361	*FCN2*	2220
CXCL6	6372	*MT-CYB*	4519	*BNIP3*	664
ALG10	84920	*HOXA3*	3200	*MT-TQ*	4572
ITPKC	80271	*MT-TI*	4565	*TAFAZZIN*	6901
ITGB3	3690	*MT-ATP8*	4509	*APLN*	8862
TBC1D32	221322	*MT-TL2*	4568	*KLHL41*	10324
GPD1L	23171	*LCK*	3932	*APOC2*	344

Supplementary Table S2 (continued)

Gene Symbol	Entrez ID	Gene Symbol	Entrez ID	Gene Symbol	Entrez ID
POLR2A	5430	*TUBA8*	51807	*CD14*	929
CXCL10	3627	*LHX3*	8022	*GPC4*	2239
MIR34B	407041	*PIK3CA*	5290	*PPP1R13L*	10848
POMC	5443	*MIR20A*	406982	*FRMD4B*	23150
FOXF2	2295	*TOP2A*	7153	*MMP9*	4318
FBXO6	26270	*PKD1L1*	168507	*XDH*	7498
MKS1	54903	*TTF1*	7270	*IRAK1*	3654
CX3CR1	1524	*RELA*	5970	*CXCL3*	2921
KISS1	3814	*METTL27*	155368	*EP300*	2033
WFDC6	140870	*TDGF1*	6997	*ERG*	2078
MT2A	4502	*HLA-B*	3106	*DVL1P1*	8215
HEXA	3073	*SCUBE2*	57758	*AGPAT1*	10554
MIR346	442911	*ZNF462*	58499	*NOSTRIN*	115677
MIR10B	406903	*SLX4IP*	128710	*MIRLET7I*	406891
SI	6476	*PPP1R26*	9858	*GRK5*	2869
ITGAL	3683	*PIGU*	128869	*FOXP3*	50943
CHST3	9469	*STC2*	8614	*WDR45*	11152
KANK2	25959	*COL6A1*	1291	*TGM6*	343641
HYAL1	3373	*SGCE*	8910	*RETN*	56729
GBX1	2636	*AKR1C3*	8644	*LATS1*	9113
ATG9B	285973	*COA6*	388753	*DDX6*	1656
MACF1	23499	*DDX3X*	1654	*AKAP9*	10142
RAP1A	5906	*PNPO*	55163	*IGLC1*	3537
MYO6	4646	*CD40LG*	959	*SLC20A1*	6574
NGF	4803	*CYP3A4*	1576	*EFEMP2*	30008
CYSLTR2	57105	*CENPF*	1063	*VCAN*	1462
MIR188	406964	*C6*	729	*GTF3C4*	9329
CCDC22	28952	*BCRP3*	644165	*CCL28*	56477
USP10	9100	*S100A9*	6280	*DUT*	1854
BCL2L2	599	*MIR206*	406989	*CTSS*	1520
AGK	55750	*KCNJ2*	3759	*UQCRFS1*	7386
ALOX15	246	*ATP9B*	374868	*MIR1-2*	406905
CCL7	6354	*MSH3*	4437	*FHL2*	2274
TP53INP2	58476	*BAK1*	578	*CSF3*	1440
BRK1	55845	*ATP5F1D*	513	*RAD51C*	5889
EGR2	1959	*SYNGAP1*	8831	*PLEKHA7*	144100
NGB	58157	*GGT2*	728441	*SLPI*	6590
MIR30A	407029	*MIR125A*	406910	*MT-CO3*	4514
TPRN	286262	*ITM2B*	9445	*GJC1*	10052
SMAD4	4089	*GLT8D2*	83468	*SFRP4*	6424
VASH1	22846	*HSPA1L*	3305	*RALGDS*	5900
ELMOD2	255520	*ASB10*	136371	*CTSD*	1509
HAVCR1	26762	*BCL2L10*	10017	*SMAD2*	4087
CDC45	8318	*PRDM16*	63976	*FOXC2*	2303
IL6	3569	*ZIC3*	7547	*PGF*	5228
ABCG2	9429	*ATP6V1E1*	529	*HSP90AA1*	3320
TRAK2	66008	*BIRC2*	329	*VPREB1*	7441
ATP5MG	10632	*TIMP1*	7076	*ZC3HC1*	51530
POLR2K	5440	*MT-TS1*	4574	*TNFSF11*	8600
C8B	732	*CCR4*	1233	*CYP4F2*	8529

Supplementary Table S2 (continued)

Gene Symbol	Entrez ID	Gene Symbol	Entrez ID	Gene Symbol	Entrez ID
LCN10	414332	*MIR376A1*	494325	*MIR148B*	442892
FBXO11	80204	*MIR34C*	407042	*NPPC*	4880
CDK8	1024	*NCF1*	653361	*SFTA3*	253970
CHST14	113189	*KLK1*	3816	*SALL4*	57167
DNAH11	8701	*KCNJ5*	3762	*PRKAR1B*	5575
POMT1	10585	*PRAME*	23532	*TCF3*	6929
SARS1	6301	*DPAGT1*	1798	*GLB1*	2720
CGA	1081	*MYO18B*	84700	*MMP10*	4319
LRRC10	376132	*MED27*	9442	*OBP2B*	29989
GNB4	59345	*MIR296*	407022	*TCN2*	6948
GSTM3	2947	*ELMOD1*	55531	*DNAI2*	64446
WDR5	11091	*NPHP4*	261734	*ACKR1*	2532
TMEM121B	27439	*ZBTB33*	10009	*REPIN1*	29803
ACVR1B	91	*MGA*	23269	*CAPZB*	832
IFNGR1	3459	*MED1*	5469	*FGL2*	10875
PSORS1C2	170680	*CTXN2*	399697	*PCDH12*	51294
LCN6	158062	*HADH*	3033	*RXFP4*	339403
MT-TK	4566	*SCO2*	9997	*PDZD3*	79849
HLA-DRB1	3123	*MAP1LC3A*	84557	*PXMP4*	11264
FOXN1	8456	*GTF2I*	2969	*RNF167*	26001
ACSL6	23305	*SSPN*	8082	*FARSB*	10056
GIMAP5	55340	*CFLAR*	8837	*GPSM1*	26086
REG3G	130120	*MAPK8IP3*	23162	*THUMPD3*	25917
IL12A	3592	*IFIH1*	64135	*SMARCA4*	6597
PDCD10	11235	*ADGRL4*	64123	*TMEM43*	79188
UBE4A	9354	*CDKN2B*	1030	*GLIS3*	169792
SIAH1	6477	*ITGA2*	3673	*ITGB1BP2*	26548
SLC37A4	2542	*MIR10A*	406902	*GIMAP1*	170575
ENG	2022	*WDR26*	80232	*PPP1R9A*	55607
SULT1E1	6783	*GYG1*	2992	*MIR181C*	406957
HLA-DPB1	3115	*CCNA2*	890	*MIR26B*	407017
MAP2K7	5609	*DNASE1L1*	1774	*SMAD9*	4093
FCGR3B	2215	*MT-ND6*	4541	*MED12*	9968
SMPD3	55512	*RSPH4A*	345895	*OXSR1*	9943
TRPC6	7225	*SERPINA4*	5267	*ENPP1*	5167
SOCS1	8651	*NDE1*	54820	*CUL4A*	8451
STRA6	64220	*SYNPO2L*	79933	*CCR5*	1234
MT-ND5	4540	*ASB4*	51666	*PDLIM3*	27295
MAN1B1	11253	*CCL23*	6368	*SCN8A*	6334
GPR17	2840	*SH3PXD2B*	285590	*MT-TT*	4576
NDUFB11	54539	*NDUFS1*	4719	*IRF8*	3394
IL18BP	10068	*MIR423*	494335	*F2RL3*	9002
DCX	1641	*MT-ATP6*	4508	*BIK*	638
CYP3A5	1577	*CECR2*	27443	*POGLUT3*	143888
RDX	5962	*GJB2*	2706	*FASN*	2194
SLC34A3	142680	*PRKD1*	5587	*KMT2A*	4297
IFNL3	282617	*RAPGEF3*	10411	*CASP10*	843
MIR369	442914	*TGOLN2*	10618	*TCAP*	8557
UCN2	90226	*FGD1*	2245	*VIP*	7432
CFAP52	146845	*TRAF1*	7185	*MC2R*	4158

Supplementary Table S2 (continued)

Gene Symbol	Entrez ID	Gene Symbol	Entrez ID	Gene Symbol	Entrez ID
MT-TY	4579	*ANGPTL5*	253935	*MIR19A*	406979
CIITA	4261	*COL4A5*	1287	*TRPM4*	54795
MIR197	406974	*FEV*	54738	*LTB4R*	1241
ZFYVE16	9765	*CLDN16*	10686	*B3GAT2*	135152
POLE	5426	*ADH5*	128	*ITGA4*	3676
DNAH8	1769	*SETX*	23064	*CLOCK*	9575
BCL7B	9275	*NEU3*	10825	*DSG4*	147409
AGTRAP	57085	*DES*	1674	*GSTA1*	2938
MT-ND2	4536	*BZW1*	9689	*WNK2*	65268
TNFSF12	8742	*HSD3B1*	3283	*NBEAL1*	65065
TMEM25	84866	*EIF4H*	7458	*UCN*	7349
CLTCL1	8218	*CD2AP*	23607	*MECP2*	4204
SLC25A18	83733	*CCL20*	6364	*MIR30B*	407030
BMP8A	353500	*PCK1*	5105	*PLN*	5350
MAP1LC3B	81631	*VWF*	7450	*GJB6*	10804
P2RY8	286530	*SLC12A9*	56996	*MIR21*	406991
MYOZ1	58529	*TWIST1*	7291	*PON1*	5444
RANBP2	5903	*MIR331*	442903	*CXCR3*	2833
TGFBI	7045	*MEF2C*	4208	*ST6GALNAC4*	27090
HSPG2	3339	*TMUB1*	83590	*UBR4*	23352
FANCB	2187	*MIR23B*	407011	*FBXL22*	283807
GALNTL5	168391	*MYB*	4602	*AP1B1*	162
HDAC6	10013	*XRCC2*	7516	*KLHL36*	79786
HBA1	3039	*VPS37D*	155382	*MIR196B*	442920
ADIPOQ	9370	*UGCG*	7357	*SCN1B*	6324
ITGA5	3678	*IL12RB1*	3594	*CYBB*	1536
LDB3	11155	*MT-RNR1*	4549	*NDUFA1*	4694
LBP	3929	*ARHGAP20*	57569	*MIR335*	442904
AICDA	57379	*IHH*	3549	*MT-TH*	4564
CACNA1B	774	*HAS3*	3038	*FGF5*	2250
RHOA	387	*MIR193A*	406968	*SRC*	6714
VILL	50853	*GLO1*	2739	*TCF21*	6943
IL13RA2	3598	*PPIL3*	53938	*RER1*	11079
AOC3	8639	*EFNA5*	1946	*THPO*	7066
MT1F	4494	*COL11A2*	1302	*RNF213*	57674
TMEM250	90120	*BPNT1*	10380	*CD70*	970
MIR1-1HG	128826	*PPP3R1*	5534	*SLC29A3*	55315
DCHS1	8642	*EIF1AX*	1964	*CYP4F12*	66002
SCN10A	6336	*FCN1*	2219	*YPEL1*	29799
GULOP	2989	*GBGT1*	26301	*BRAP*	8315
MCFD2	90411	*ZBTB20*	26137	*RFC2*	5982
PEX26	55670	*RPUSD3*	285367	*CASP12*	100506742
PRDM6	93166	*NAMPT*	10135	*LEFTY2*	7044
PRF1	5551	*SMIM11A*	54065	*MN1*	4330
SCFD1	23256	*IL23R*	149233	*MYH15*	22989
MT-TC	4511	*PARG*	8505	*ACAD9*	28976
KLF9	687	*CASP5*	838	*ATP5PO*	539
MMP12	4321	*RAB36*	9609	*ADM2*	79924
HABP2	3026	*SGCG*	6445	*SUV39H1*	6839
DNAAF11	23639	*HYDIN*	54768	*CHD3*	1107

Supplementary Table S2 (continued)

Gene Symbol	Entrez ID	Gene Symbol	Entrez ID	Gene Symbol	Entrez ID
BCAP31	10134	*MYL4*	4635	*BMP6*	654
EXOSC10	5394	*ZDHHC7*	55625	*HKDC1*	80201
KDM5C	8242	*MMP8*	4317	*GOLGA6B*	55889
ARL13B	200894	*CCDC3*	83643	*CYP2J2*	1573
SERPINA1	5265	*KIAA0513*	9764	*FLNA*	2316
ESRRG	2104	*GIMAP4*	55303	*LRPAP1*	4043
KLF6	1316	*TNFSF8*	944	*PEPD*	5184
THBD	7056	*MIR126*	406913	*CYRIB*	51571
CYP17A1	1586	*DSG3*	1830	*PNPLA2*	57104
CCDC183	84960	*COX7A1*	1346	*TMEM260*	54916
CFB	629	*INPP5D*	3635	*CSF2*	1437
CASP9	842	*CHRD*	8646	*GDF2*	2658
BNIP3L	665	*ADH1B*	125	*XRCC4*	7518
RAB1A	5861	*CXCL5*	6374	*TAC3*	6866
POU5F1	5460	*TRAPPC4*	51399	*F2*	2147
KMT2D	8085	*FCN3*	8547	*UPK2*	7379
MUC13	56667	*NPY2R*	4887	*WNK4*	65266
TDRD7	23424	*MIR383*	494332	*NLRP13*	126204
MIR330	442902	*GSTT2*	2953	*CCL4*	6351
ALPK1	80216	*MIB2*	142678	*USP18*	11274
RAB7A	7879	*ANG*	283	*ZNF354A*	6940
APOM	55937	*S100A1*	6271	*SLC35F1*	222553
HLA-DQA1	3117	*MT1X*	4501	*ATG9A*	79065
ZMYND10	51364	*S100A8*	6279	*ZNF74*	7625
SLC22A3	6581	*MT-ND1*	4535	*ADGRL3*	23284
MIR337	442905	*HHIPL1*	84439	*BMP10*	27302
CCL11	6356	*ZCCHC14*	23174	*KCNQ1*	3784
CREG1	8804	*SLCO1B3*	28234	*CTTNBP2*	83992
FNDC5	252995	*RAB8B*	51762	*C9orf116*	138162
MIR200A	406983	*GRK6*	2870	*TRPM7*	54822
CXCL9	4283	*ATP5ME*	521	*ADA2*	51816
C20orf144	128864	*IFNA2*	3440	*PNPLA7*	375775
MIR194-1	406969	*YWHAE*	7531	*ATG5*	9474
FOXF1	2294	*C5AR1*	728	*ZMYND19*	116225
ZNF280B	140883	*STRADB*	55437	*IL17RD*	54756
KCNQ1OT1	10984	*MIR15A*	406948	*XIAP*	331
HLA-DMB	3109	*HEY2*	23493	*DKK3*	27122
HLA-A	3105	*ARHGDIA*	396	*SMAD1*	4086
SEPTIN9	10801	*BMPR1B*	658	*PDLIM4*	8572
CDK5R2	8941	*CENPC*	1060	*CBX7*	23492
IFT122	55764	*RHEB*	6009	*ADORA3*	140
HSPA1A	3303	*MAPRE1*	22919	*RAI2*	10742
MIR221	407006	*ABI2*	10152	*EIF4EBP1*	1978
MT-TM	4569	*WDR12*	55759	*TERT*	7015
RNF123	63891	*NR2C2*	7182	*CCL1*	6346
MT-TV	4577	*AURKA*	6790	*KAT6B*	23522
PHLDB2	90102	*PHACTR1*	221692	*KDM4D*	55693
GLT6D1	360203	*MYOD1*	4654	*BTNL2*	56244
TRADD	8717	*GAA*	2548	*MX1*	4599
SCN3B	55800	*IL1RL2*	8808	*RELB*	5971

Supplementary Table S2 (continued)

Gene Symbol	Entrez ID	Gene Symbol	Entrez ID	Gene Symbol	Entrez ID
NDRG4	65009	*MB*	4151	*STING1*	340061
TNFRSF6B	8771	*LIFR*	3977	*CCDC116*	164592
PTPRU	10076	*CYP4Z1*	199974	*ADAD2*	161931
ADPGK	83440	*ACAT2*	39	*C9orf163*	158055
RPS25	6230	*JARID2*	3720	*IL17RE*	132014
FUT7	2529	*CELSR1*	9620	*GUF1*	60558
MT-TD	4555	*NEXN*	91624	*TMEM176A*	55365
TLR2	7097	*GATA5*	140628	*FSIP2*	401024
ASIC3	9311	*SEC16A*	9919	*DNAAF3*	352909
MIR145	406937	*NOTCH4*	4855	*CENATAC*	338657
EPHB4	2050	*EDEM2*	55741	*TTLL10*	254173
SERPINA10	51156	*ST6GALNAC6*	30815	*CCDC89*	220388
CD27	939	*B4GALT7*	11285	*LINC01599*	196913
MT-TL1	4567	*MPZL3*	196264	*TMEM270*	135886
CCL13	6357	*THOC6*	79228	*ZFAND2B*	130617
PLOD1	5351	*EMC3*	55831	*MORN1*	79906
NPDC1	56654	*LRP2BP*	55805	*CELA2A*	63036
HES5	388585	*FAM193B*	54540	*CEP57*	9702
PKD2	5311	*ATL3*	25923	*SNORD70*	692110
PUSL1	126789	*SMCHD1*	23347	*DIPK1B*	138311
AKAP14	158798	*TUBB4B*	10383	*KIAA2013*	90231
HLA-E	3133	*LCNL1*	401562	*TMEM141*	85014
FLT3LG	2323	*C1orf185*	284546	*NHEJ1*	79840
FOXC1	2296	*EIF4E1B*	253314	*ANKZF1*	55139
C3AR1	719	*TMCO1*	54499	*PYCARD*	29108
SPACA9	11092	*NCAPH2*	29781	*SRSF8*	10929
CFH	3075	*AFF4*	27125	*SNORD11*	692058
JAG1	182	*KALRN*	8997	*ZNF526*	116115
HLA-DQB1	3119	*NTMT2*	149281	*MYSM1*	114803
OGG1	4968	*DCUN1D5*	84259	*TSR2*	90121
HDAC9	9734	*PNPLA3*	80339	*SNHG7*	84973
ARG1	383	*UIMC1*	51720	*CCDC82*	79780
CFC1	55997	*CCDC103*	388389	*EGFL7*	51162
P2RY12	64805	*CATIP*	375307	*RANGRF*	29098
CIDEC	63924	*DAW1*	164781	*PRDX6*	9588
MT-TW	4578	*IFT43*	112752	*KLHL22*	84861
TRIB1	10221	*ODAD1*	93233	*ATP1A1-AS1*	84852
S100A6	6277	*LYRM7*	90624	*MICAL3*	57553
TBL2	26608	*ZC3H12C*	85463	*DISP3*	57540
HSDL1	83693	*NUB1*	51667	*CFAP92*	57501
ANKRD31	256006	*GINS2*	51659	*INTS11*	54973
EIF2AK3	9451	*ENDOD1*	23052	*TMEM70*	54968
SCN2A	6326	*PARP2*	10038	*SSUH2*	51066
PHLDB1	23187	*HOATZ*	399949	*WDPCP*	51057
KCNK2	3776	*ENTPD8*	377841	*MIR598*	693183
TNC	3371	*MITD1*	129531	*MIR590*	693175
PHF6	84295	*ALKBH8*	91801	*MIR582*	693167
DNMT1	1786	*ZC3H12A*	80149	*MIR532*	693124
SBF2	81846	*MGAT4B*	11282	*MIR411*	693121
ETFDH	2110	*ACSL3*	2181	*TMEM86B*	255043

Supplementary Table S2 (continued)

Gene Symbol	Entrez ID	Gene Symbol	Entrez ID	Gene Symbol	Entrez ID
TLCD5	219902	*LRRC26*	389816	*CDKN2C*	1031
C11orf65	160140	*LCN15*	389812	*MT1HL1*	645745
RHOXF1	158800	*RNF207*	388591	*DDI1*	414301
ODAD3	115948	*C1QTNF12*	388581	*LHFPL4*	375323
SRI	6717	*CDKL4*	344387	*PFN3*	345456
LMOD2	442721	*CIBAR2*	339145	*LAYN*	143903
TMEM52	339456	*CYP4A22*	284541	*LRRC56*	115399
TMEM240	339453	*TICAM1*	148022	*TSLP*	85480
LINC01588	283551	*MTPN*	136319	*CFAP74*	85452
SOX5	6660	*DNAJC19*	131118	*WDR75*	84128
CYSRT1	375791	*PRXL2B*	127281	*CCDC92*	80212
FAM83H	286077	*ZPBP2*	124626	*SLC30A10*	55532
REEP3	221035	*HVCN1*	84329	*ERRFI1*	54206
SRFBP1	153443	*VWA1*	64856	*CYCS*	54205
LINC00477	144360	*VPS35L*	57020	*EXPH5*	23086
NKX2-6	137814	*CHPF2*	54480	*PQBP1*	10084
FANCD2OS	115795	*PRELID1*	27166	*HDAC5*	10014
CPLANE2	79363	*INVS*	27130	*TNFRSF18*	8784
TMEM135	65084	*PAMR1*	25891	*SNAP23*	8773
TMEM106B	54664	*NIPBL*	25836	*CDC25B*	994
UBIAD1	29914	*CCL5*	6352	*CDK11A*	728642
CLEC10A	10462	*CLEC11A*	6320	*CDK11B*	984
MIR503	574506	*REG3A*	5068	*CD40*	958
MIR502	574504	*CD82*	3732	*CD86*	942
MIR499A	574501	*LYST*	1130	*C11orf87*	399947
SLC6A17	388662	*CHKA*	1119	*MYLK4*	340156
ZNF775	285971	*COA5*	493753	*TECRL*	253017
ABRA	137735	*MIR17HG*	407975	*ATAD3C*	219293
MSANTD4	84437	*CFAP77*	389799	*NIBAN3*	199786
MMEL1	79258	*ANKRD37*	353322	*KHDC3L*	154288
CASD1	64921	*C1orf167*	284498	*B3GLCT*	145173
CREBZF	58487	*CCDC153*	283152	*C11orf52*	91894
DENND11	57189	*FAAP20*	199990	*CEP290*	80184
RALGAPB	57148	*CPNE9*	151835	*CTC1*	80169
CAMK1D	57118	*SGF29*	112869	*LRRC8E*	80131
TMEM126B	55863	*TMEM126A*	84233	*CWC15*	51503
HINFP	25988	*CD276*	80381	*HIKESHI*	51501
NELFB	25920	*PDGFD*	80310	*CEP152*	22995
RPGRIP1L	23322	*C11orf49*	79096	*ATXN2L*	11273
KNG1	3827	*VKORC1*	79001	*DLEC1*	9940
CCR2	729230	*MTARC1*	64757	*PLPP3*	8613
RNF208	727800	*IFT46*	56912	*CASP2*	835
MIR493	574450	*IARS2*	55699	*FAM163B*	642968
MIR146B	574447	*INTS8*	55656	*MIR433*	574034
MIR376B	574435	*PPA2*	27068	*MIR20B*	574032
MIR452	574412	*TRMT2A*	27037	*MIR362*	574030
MIR451A	574411	*RAB3GAP2*	25782	*NRARP*	441478
GIMAP6	474344	*GSE1*	23199	*STPG3*	441476
FAM166A	401565	*FAF2*	23197	*SLC27A1*	376497
LINC02908	401563	*FXN*	2395	*USP41*	373856

Supplementary Table S2 (continued)

Gene Symbol	Entrez ID	Gene Symbol	Entrez ID	Gene Symbol	Entrez ID
WDR86	349136	*RFFL*	117584	*TMTC4*	84899
SLC6A19	340024	*TMEM123*	114908	*ORAI1*	84876
MAMDC4	158056	*TMEM203*	94107	*AGBL4*	84871
YDJC	150223	*CFAP300*	85016	*RTL10*	79680
AIFM3	150209	*CRISPLD2*	83716	*FASTKD1*	79675
SPATA17	128153	*METTL8*	79828	*NLRX1*	79671
PIH1D2	120379	*AFAP1*	60312	*DYNC2H1*	79659
CCDC32	90416	*CARD18*	59082	*SRD5A3*	79644
EIF2A	83939	*GATAD1*	57798	*CEP126*	57562
PKD1L1-AS1	80099	*MEAK7*	57707	*AURKAIP1*	54998
DCAF17	80067	*PBRM1*	55193	*MTARC2*	54996
SEMA6D	80031	*DARS2*	55157	*C1orf159*	54991
WWC2	80014	*CD274*	29126	*EXD3*	54932
MTMR14	64419	*SSU72*	29101	*YARS2*	51067
LGR4	55366	*ACOT2*	10965	*RRP15*	51018
MNS1	55329	*FASTK*	10922	*TMEM176B*	28959
UFSP2	55325	*PNPLA6*	10908	*LAMTOR2*	28956
KHDC4	22889	*WSCD2*	9671	*MASP2*	10747
ADNP2	22850	*MARF1*	9665	*PTGES3*	10728
WASHC5	9897	*TFAM*	7019	*MED22*	6837
NUAK1	9891	*HMGB1*	3146	*PDIA3*	2923
KEAP1	9817	*HBEGF*	1839	*FASLG*	356
TTN	7273	*DRD3*	1814	*APEX1*	328
ERN1	2081	*ACOT1*	641371	*C16orf74*	404550
C1S	716	*MIR429*	554210	*TEX9*	374618
CCDC30	728621	*SLC25A35*	399512	*PAXX*	286257
CARD17	440068	*C11orf53*	341032	*CCDC110*	256309
SOHLH1	402381	*FAM126B*	285172	*MPV17L*	255027
MIA3	375056	*IAH1*	285148	*TUBB*	203068
PRRT3	285368	*DRC1*	92749	*A2ML1*	144568
SLC9B1	150159	*C1orf115*	79762	*ACTRT2*	140625
FAM76B	143684	*PPCS*	79717	*TANGO2*	128989
PIK3IP1	113791	*TMBIM1*	64114	*C22orf39*	128977
HIF3A	64344	*CCDC40*	55036	*FNIP1*	96459
CCDC81	60494	*TMEM255A*	55026	*TUBA1C*	84790
YY1AP1	55249	*SRCAP*	10847	*ABHD11*	83451
MACO1	55219	*CREB5*	9586	*SAP130*	79595
NKAPD1	55216	*HINT1*	3094	*NKAP*	79576
SETD5	55209	*DNMT3B*	1789	*NADK*	65220
UBR5	51366	*DNMT3A*	1788	*CYP20A1*	57404
SNRNP27	11017	*ATM*	472	*CASZ1*	54897
TMEM94	9772	*MIR542*	664617	*BCOR*	54880
TMOD1	7111	*FIBIN*	387758	*TOR4A*	54863
RAC1	5879	*ARMS2*	387715	*ANKRD49*	54851
MT-CO2	4513	*CFAP65*	255101	*CDHR2*	54825
HES1	3280	*CCDC83*	220047	*BACE1*	23621
CBY3	646019	*DNAAF1*	123872	*TSSK2*	23617
DRAXIN	374946	*CEP20*	123811	*IGF2BP1*	10642
CCDC39	339829	*GPRIN1*	114787	*STAMBP*	10617
RMDN2	151393	*CARD16*	114769	*GRK4*	2868

Supplementary Table S2 (continued)

Gene Symbol	Entrez ID	Gene Symbol	Entrez ID	Gene Symbol	Entrez ID
CYP19A1	1588	*MIR301B*	100126318	*COL4A2-AS2*	100129836
CYP2C8	1558	*MIR744*	100126313	*LRRD1*	401387
MIAT	440823	*MIR671*	768213	*RNF224*	643596
BRINP3	339479	*MIR758*	768212	*MIR4428*	100616141
HYLS1	219844	*RIMBP3B*	440804	*MIR4761*	100616414
C1orf127	148345	*RIMBP3C*	150221	*MIR1245B*	100616324
GCOM1	145781	*RIMBP3*	85376	*MYLK-AS1*	100506826
TMEM67	91147	*GGTLC3*	728226	*FPGT-TNNI3K*	100526835
WDR25	79446	*TET3*	200424	*ANKRD65*	441869
SMG9	56006	*TMEM210*	100505993	*MYZAP*	100820829
TET2	54790	*LRRC53*	105378803	*KCNQ1-AS1*	338653
ARHGAP45	23526	*TTC36*	143941	*FNDC10*	643988
MTREX	23517	*TTC34*	100287898	*CACNA1C-IT3*	100874370
TXNRD2	10587	*C2orf74*	339804	*COL4A2-AS1*	100874203
HYOU1	10525	*C4orf47*	441054	*CACNA1C-AS4*	100874234
PDLIM7	9260	*MIR155*	406947	*CACNA1C-AS2*	100874235
PRRX1	5396	*ZNF862*	643641	*NDP-AS1*	100873919
GLI2	2736	*NPHP3-AS1*	348808	*OPA1-AS1*	100873941
NKX2-5	1482	*DBH-AS1*	138948	*FENDRR*	400550
CS	1431	*FDXACB1*	91893	*CACNA1C-AS1*	100652846
AGMO	392636	*CDKN2B-AS1*	100048912	*SRD5A3-AS1*	100506462
AGRN	375790	*RNU6ATAC*	100151684	*SBF2-AS1*	283104
MYBPHL	343263	*RNU4ATAC*	100151683	*TTN-AS1*	100506866
GATC	283459	*FBRSL1*	57666	*RTEL1-TNFRSF6E*	100533107
HECTD4	283450	*TMPPE*	643853	*SLC26A4-AS1*	286002
FBXO44	93611	*RESP18*	389075	*TMPO-AS1*	100128191
RTN4R	65078	*SDHAF1*	644096	*ACVR2B-AS1*	100128640
OSGIN1	29948	*KDM4E*	390245	*LOXL1-AS1*	100287616
SPATS2L	26010	*MIR1245A*	100302219	*TBX5-AS1*	255480
NPTXR	23467	*COLCA2*	120376	*PRKAG2-AS1*	100505483
KCNAB1	7881	*TMEM88B*	643965	*MCIDAS*	345643
TUBA1A	7846	*HSBP1L1*	440498	*CCL15-CCL14*	348249
GBA	2629	*NPPA-AS1*	100379251	*LINC00683*	400660
NACAD	23148	*MIR548L*	100302275	*IFNL4*	101180976
MEX3A	92312	*MIR1285-1*	100302218	*FAM230A*	653203
LINC02869	440714	*LIPT2*	387787	*RPL36A-HNRNPH2*	100529097
ATRIP	84126	*C5orf60*	285679	*TEX41*	401014
SNORD11B	100113392	*MYMK*	389827	*HAND2-AS1*	79804
RHOXF2B	727940	*AJM1*	389813	*EDNRB-AS1*	100505518
RHOXF2	84528	*IGLL5*	100423062	*LINC01082*	100506542
TMEM191C	645426	*MIR761*	100313892	*P2RX5-TAX1BP3*	100533970
MOXD2P	100289017	*ARHGAP42*	143872	*NPHP3-ACAD11*	100532724
TMEM191B	728229	*MIR620*	693205	*LINC01142*	284688
CC2D2A	57545	*MIR4251*	100422968	*APELA*	100506013
CPTP	80772	*MIR3129*	100422908	*ITGA9-AS1*	101928153
DNLZ	728489	*SLC35E2B*	728661	*MIR6076*	102464828
TMEM202	338949	*MIR3606*	100500837	*MIR6804*	102465482
CFC1B	653275	*ARPC4-TTLL3*	100526693	*MIR6870*	102465525
SLC38A8	146167	*PRSS56*	646960	*MIR6803*	102466739
MIR208B	100126336	*RNF223*	401934	*MIR6500*	102466656

Supplementary Table S2 (continued)

Gene Symbol	Entrez ID	Gene Symbol	Entrez ID	Gene Symbol	Entrez ID
MIR6802	102465481	*FRA11B*	109280160	*LOC644285*	644285
MIR6795	102465476	*SEPTIN5*	5413	*LOC111811965*	111811965
TARID	100507308	*ALDOA*	226	*LOC110121264*	110121264
LYPLAL1-DT	643723	*WNT3*	7473	*LOC100287329*	100287329
LINC01426	100506385	*RUNX1*	861	*LOC114827827*	114827827
ZNF341-AS1	101929746	*HFE-AS1*	108783645	*LOC110121288*	110121288
LINC01879	400661	*SGO1-AS1*	100874028	*LOC113748410*	113748410
CYP51A1-AS1	613126	*SCN1A-AS1*	101929680	*LOC110121266*	110121266
ACTA2-AS1	100132116	*LOC112694687*	112694687	*LOC112872299*	112872299
FSIP2-AS1	107985781	*LOC110120888*	110120888	*LOC112997581*	112997581
DSCAS	101927698	*LOC110599580*	110599580	*LOC106029312*	106029312
TGFB2-OT1	103611157	*LOC107982234*	107982234	*LOC111413015*	111413015
ZFPM2-AS1	102723356	*LOC110121276*	110121276	*LOC111365225*	111365225
PTOV1-AS2	101928378	*LOC112340382*	112340382	*LOC114827850*	114827850
TGFB2-AS1	728463	*LOC107303340*	107303340	*LOC111875823*	111875823
DSG2-AS1	100652770	*DES-LCR*	106866982	*LOC114827851*	114827851
LINC01389	102724077	*LOC110121281*	110121281	*LOC110121232*	110121232
SMAD1-AS1	104326058	*LOC110121269*	110121269	*LOC106783508*	106783508
APOA1-AS	104326055	*LOC101928961*	101928961	*LOC110673971*	110673971
TET2-AS1	104384744	*LOC110121278*	110121278	*LOC110120689*	110120689
MHRT	104564225	*LOC112577579*	112577579	*LOC110121277*	110121277
WASHC5-AS1	106479020	*LOC110973015*	110973015	*LOC107988032*	107988032
SNORA99	106635531	*NRIP3-DT*	105376541	*PKD2L2-DT*	101928005
LINC01710	105372925	*LOC106501713*	106501713	*LOC112694764*	112694764
LINC02206	102723481	*LOC110121274*	110121274	*LOC102723493*	102723493
SNORD70B	109616995	*LOC101448202*	101448202	*LOC106780803*	106780803
MIR3936HG	553103	*LOC108903149*	108903149	*LOC108353820*	108353820
FLNC-AS1	110806300	*LOC110121265*	110121265	*LOC108903148*	108903148
TRDN-AS1	101927990	*LOC110120751*	110120751	*LOC112694688*	112694688
TPM1-AS	111064646	*LOC113939944*	113939944	*LOC112543469*	112543469
ELN-AS1	107986809	*LOC112694766*	112694766	*LOC113748416*	113748416
CSRP3-AS1	105376580	*LOC111365216*	111365216	*LOC110121223*	110121223
CACNA2D1-AS1	101927356	*LOC112806077*	112806077	*LOC112935930*	112935930
LDLR-AS1	115271120	*LOC111465007*	111465007	*LOC113939949*	113939949
IL6-AS1	541472	*LOC110121279*	110121279	*LOC120285841*	120285841
MADD-AS1	101928943	*LOC111413043*	111413043	*LOC120851201*	120851201
LAMA4-AS1	101927640	*LOC107303339*	107303339	*LOC120908908*	120908908
RNF213-AS1	100294362	*LOC110121486*	110121486	*LOC121056754*	121056754
PPM1F-AS1	100286925	*LOC112935929*	112935929	*LOC121175350*	121175350
CFAP298-TCP10L	110091775	*LOC110121287*	110121287	*LOC121366042*	121366042
AL162586.1	102723566	*LOC112577486*	112577486	*LOC121331339*	121331339
AC009264.1	349160	*LOC110121286*	110121286		
GJD2-DT	101928174	*FBN1-DT*	105370809		
AC009123.1	654780	*LOC112577578*	112577578		
AC107953.2	101927513	*LOC110121275*	110121275		
AC011092.2	101928008	*LOC110120917*	110120917		
AL590627.1	651337	*AGK-DT*	105375538		
AC109446.2	102723692	*LOC112679198*	112679198		
AP002008.4	728196	*LOC110121280*	110121280		
FKBP14-AS1	105375215	*LOC112577524*	112577524		

Supplementary Table S3. Clinical characteristics of the study subjects.

	**Natural conception (n = 27)**	**IVF (n = 5)**	**ICSI (n = 8)**	***P* value**
Maternal age (years) [a]	33.67 ± 4.45	37.40 ± 2.07	34.50 ± 2.39	NS[b]
Pre-pregnant BMI	22.65 ± 2.67	19.98 ± 1.51	27.65 ± 5.18	0.002[b]
Gestational age	38.69 ± 1.59	40.03 ± 1.47	38.14 ± 2.50	NS[b]
Sex of child (Males,	12 males, 15 females	3 males, 2 females	3 males, 5 females	NS[c]

[a] Mean ± Standard Deviation. [b] ANOVA, analysis of variance. [c] Fisher's exact test. BMI = body weight (kg) / body height^2 (m^2). BMI, body mass index. NS, non-significant.

Supplementary Table S4. Genes with deH3K4me3 in IVF and ICSI children

Group	Term	entrezID	logFC	*P*-Value	FDR	up_down
**boy_ICSI-ET_vs_boy_ctrl**						
boy_ICSI-ET_vs_boy_ctrl	*C20orf112*	140688	4.92599587	5.97E-45	1.21E-40	up
boy_ICSI-ET_vs_boy_ctrl	*UNC119*	9094	5.34639336	1.58E-23	1.60E-19	up
boy_ICSI-ET_vs_boy_ctrl	*RP11-410N8.4*	NA	4.33050403	7.40E-23	4.99E-19	up
boy_ICSI-ET_vs_boy_ctrl	*PRODH2*	58510	5.13961245	1.43E-19	7.24E-16	up
boy_ICSI-ET_vs_boy_ctrl	*SGK494*	124923	4.14355436	1.61E-15	6.50E-12	up
boy_ICSI-ET_vs_boy_ctrl	*AC004017.1*	NA	3.66329263	3.51E-12	1.18E-08	up
boy_ICSI-ET_vs_boy_ctrl	*MSH5-SAPCD1*	100532732	7.70851597	5.12E-11	1.29E-07	up
boy_ICSI-ET_vs_boy_ctrl	*MSH5*	4439	7.70851593	5.12E-11	1.29E-07	up
boy_ICSI-ET_vs_boy_ctrl	*SAPCD1*	401251	8.06607382	5.92E-11	1.33E-07	up
boy_ICSI-ET_vs_boy_ctrl	*DEFB125*	245938	3.12947277	3.17E-10	6.40E-07	up
boy_ICSI-ET_vs_boy_ctrl	*AGER*	177	6.34253228	9.31E-10	1.71E-06	up
boy_ICSI-ET_vs_boy_ctrl	*ZNF134*	7693	3.08911516	1.55E-09	2.61E-06	up
boy_ICSI-ET_vs_boy_ctrl	*MDC1*	9656	7.61694939	1.78E-09	2.77E-06	up
boy_ICSI-ET_vs_boy_ctrl	*PBX2*	5089	5.94833348	2.68E-09	3.86E-06	up
boy_ICSI-ET_vs_boy_ctrl	*PSENEN*	55851	3.2097434	4.25E-09	5.73E-06	up
boy_ICSI-ET_vs_boy_ctrl	*RIPK4*	54101	-3.5352735	5.56E-09	7.01E-06	down
boy_ICSI-ET_vs_boy_ctrl	*AC136604.1*	NA	3.0909514	5.90E-09	7.01E-06	up
boy_ICSI-ET_vs_boy_ctrl	*CLECL1*	160365	-1.2437235	1.70E-08	1.91E-05	down
boy_ICSI-ET_vs_boy_ctrl	*AC068533.7*	NA	1.9358157	2.61E-08	2.78E-05	up
boy_ICSI-ET_vs_boy_ctrl	*PRB1*	5542	-2.5925126	4.11E-08	4.16E-05	down
boy_ICSI-ET_vs_boy_ctrl	*U2AF1L4*	199746	2.94432129	5.37E-08	5.17E-05	up
boy_ICSI-ET_vs_boy_ctrl	*P2RX2*	22953	2.84792393	8.14E-08	6.89E-05	up
boy_ICSI-ET_vs_boy_ctrl	*ZNF611*	81856	2.78910253	8.16E-08	6.89E-05	up
boy_ICSI-ET_vs_boy_ctrl	*KIAA0100*	9703	2.72447477	8.18E-08	6.89E-05	up
boy_ICSI-ET_vs_boy_ctrl	*ZNF146*	7705	2.74796576	8.63E-08	6.98E-05	up
boy_ICSI-ET_vs_boy_ctrl	*MUC8*	100129528	2.84320103	1.14E-07	8.82E-05	up
boy_ICSI-ET_vs_boy_ctrl	*ZNF530*	348327	2.57946297	1.25E-07	9.28E-05	up
boy_ICSI-ET_vs_boy_ctrl	*MCCC2*	64087	3.8530375	1.34E-07	9.28E-05	up
boy_ICSI-ET_vs_boy_ctrl	*IGFLR1*	79713	2.76988716	1.38E-07	9.28E-05	up
boy_ICSI-ET_vs_boy_ctrl	*AHRR*	57491	1.51219472	1.38E-07	9.28E-05	up
boy_ICSI-ET_vs_boy_ctrl	*CTD-2616J11.4*	NA	2.7406032	1.44E-07	9.42E-05	up
boy_ICSI-ET_vs_boy_ctrl	*ZNF382*	84911	2.60230926	2.19E-07	0.000139	up
boy_ICSI-ET_vs_boy_ctrl	*HCST*	10870	2.81835788	2.27E-07	0.000139	up
boy_ICSI-ET_vs_boy_ctrl	*ANKRD29*	147463	1.99501274	2.56E-07	0.000148	up
boy_ICSI-ET_vs_boy_ctrl	*ZNF772*	400720	2.51415393	2.57E-07	0.000148	up
boy_ICSI-ET_vs_boy_ctrl	*AD000671.6*	NA	2.65306466	3.49E-07	0.000196	up
boy_ICSI-ET_vs_boy_ctrl	*ZNF776*	284309	2.45411441	4.72E-07	0.000258	up
boy_ICSI-ET_vs_boy_ctrl	*CLDND2*	125875	2.58284897	5.69E-07	0.000299	up
boy_ICSI-ET_vs_boy_ctrl	*OR7D2*	162998	2.68745085	5.77E-07	0.000299	up
boy_ICSI-ET_vs_boy_ctrl	*C5orf60*	285679	2.6284734	8.92E-07	0.000451	up
boy_ICSI-ET_vs_boy_ctrl	*ZNF154*	7710	2.5640841	9.38E-07	0.000462	up
boy_ICSI-ET_vs_boy_ctrl	*ZNF583*	147949	2.32671392	1.19E-06	0.000559	up
boy_ICSI-ET_vs_boy_ctrl	*ZNF575*	284346	2.27317966	2.04E-06	0.000935	up
boy_ICSI-ET_vs_boy_ctrl	*HOXA11*	3207	2.15372152	2.26E-06	0.000995	up
boy_ICSI-ET_vs_boy_ctrl	*NKG7*	4818	2.46723642	2.26E-06	0.000995	up
boy_ICSI-ET_vs_boy_ctrl	*ZNF805*	390980	2.42326909	2.64E-06	0.001118	up

Supplementary Table S4 (continued)

boy_ICSI-ET_vs_boy_ctrl	*DDC8*	100653515	1.81296493	2.71E-06	0.001118	up
boy_ICSI-ET_vs_boy_ctrl	*TIMP2*	7077	1.81296535	2.71E-06	0.001118	up
boy_ICSI-ET_vs_boy_ctrl	*ZFP82*	284406	2.299054	2.83E-06	0.001139	up
boy_ICSI-ET_vs_boy_ctrl	*RAB26*	25837	1.84232888	2.87E-06	0.001139	up
boy_ICSI-ET_vs_boy_ctrl	*ELP4*	26610	1.84555436	3.06E-06	0.001188	up
boy_ICSI-ET_vs_boy_ctrl	*ZNF461*	92283	2.40923036	3.56E-06	0.001359	up
boy_ICSI-ET_vs_boy_ctrl	*SH3GL2*	6456	1.70710221	3.63E-06	0.00136	up
boy_ICSI-ET_vs_boy_ctrl	*NANOS2*	339345	2.0769464	4.08E-06	0.001501	up
boy_ICSI-ET_vs_boy_ctrl	*AC004076.9*	NA	2.15212934	4.39E-06	0.00157	up
boy_ICSI-ET_vs_boy_ctrl	*PTCD3*	55037	1.58453589	4.43E-06	0.00157	up
boy_ICSI-ET_vs_boy_ctrl	*DNAH9*	1770	1.9723031	4.76E-06	0.001658	up
boy_ICSI-ET_vs_boy_ctrl	*PIGS*	94005	1.57273976	6.64E-06	0.002239	up
boy_ICSI-ET_vs_boy_ctrl	*RP11-192H23.4*	NA	1.57273969	6.65E-06	0.002239	up
boy_ICSI-ET_vs_boy_ctrl	*IGHMBP2*	3508	1.73140255	9.83E-06	0.003257	up
boy_ICSI-ET_vs_boy_ctrl	*FOXD4*	2298	1.59737657	1.23E-05	0.003955	up
boy_ICSI-ET_vs_boy_ctrl	*SNX4*	8723	1.63903893	1.23E-05	0.003955	up
boy_ICSI-ET_vs_boy_ctrl	*ZNF714*	148206	2.19518071	1.28E-05	0.004022	up
boy_ICSI-ET_vs_boy_ctrl	*ZNF726*	730087	2.34296717	1.30E-05	0.004022	up
boy_ICSI-ET_vs_boy_ctrl	*DEPDC1B*	55789	1.67605791	1.31E-05	0.004022	up
boy_ICSI-ET_vs_boy_ctrl	*COX7A1*	1346	2.34224232	1.39E-05	0.004178	up
boy_ICSI-ET_vs_boy_ctrl	*AP002353.1*	NA	1.32924589	1.43E-05	0.004178	up
boy_ICSI-ET_vs_boy_ctrl	*SLN*	6588	1.32924614	1.43E-05	0.004178	up
boy_ICSI-ET_vs_boy_ctrl	*AHSP*	51327	1.50125134	1.52E-05	0.004383	up
boy_ICSI-ET_vs_boy_ctrl	*TXNDC8*	255220	2.19387943	1.83E-05	0.005223	up
boy_ICSI-ET_vs_boy_ctrl	*RNF182*	221687	1.3871946	1.94E-05	0.005458	up
boy_ICSI-ET_vs_boy_ctrl	*PRPSAP1*	5635	1.35449321	2.13E-05	0.005885	up
boy_ICSI-ET_vs_boy_ctrl	*POFUT1*	23509	1.88147682	2.30E-05	0.006287	up
boy_ICSI-ET_vs_boy_ctrl	*ZNF468*	90333	2.09200854	2.51E-05	0.006762	up
boy_ICSI-ET_vs_boy_ctrl	*TMEM82*	388595	1.58781721	2.60E-05	0.00684	up
boy_ICSI-ET_vs_boy_ctrl	*DGCR6*	8214	3.10655948	2.61E-05	0.00684	up
boy_ICSI-ET_vs_boy_ctrl	*KIAA1045*	23349	1.5678183	2.84E-05	0.007366	up
boy_ICSI-ET_vs_boy_ctrl	*ZNF20*	7568	2.15464746	3.24E-05	0.008253	up
boy_ICSI-ET_vs_boy_ctrl	*KIF3B*	9371	2.16162029	3.27E-05	0.008253	up
boy_ICSI-ET_vs_boy_ctrl	*DNAJB14*	79982	-3.4638584	3.44E-05	0.0085	down
boy_ICSI-ET_vs_boy_ctrl	*IRF2BPL*	64207	2.11364975	3.45E-05	0.0085	up
boy_ICSI-ET_vs_boy_ctrl	*ZNF844*	284391	2.16415553	3.55E-05	0.008631	up
boy_ICSI-ET_vs_boy_ctrl	*ATAD1*	84896	1.93193741	3.59E-05	0.008631	up
boy_ICSI-ET_vs_boy_ctrl	*AARS2*	57505	2.03599249	3.67E-05	0.008733	up
boy_ICSI-ET_vs_boy_ctrl	*C1orf159*	54991	1.36680443	3.80E-05	0.008938	up
boy_ICSI-ET_vs_boy_ctrl	*ARID3C*	138715	1.30024664	4.10E-05	0.009517	up
boy_ICSI-ET_vs_boy_ctrl	*LIMS1*	3987	2.06577043	4.22E-05	0.009704	up
boy_ICSI-ET_vs_boy_ctrl	*ZNF273*	10793	2.0841535	4.35E-05	0.009865	up
boy_ICSI-ET_vs_boy_ctrl	*PROM2*	150696	2.39784359	4.39E-05	0.009865	up
boy_ICSI-ET_vs_boy_ctrl	*ZNF491*	126069	2.18753935	4.56E-05	0.01005	up
boy_ICSI-ET_vs_boy_ctrl	*CAPNS1*	826	2.0262382	4.57E-05	0.01005	up
boy_ICSI-ET_vs_boy_ctrl	*ZNF304*	57343	2.19067889	5.31E-05	0.01131	up
boy_ICSI-ET_vs_boy_ctrl	*LYPD3*	27076	2.25128179	5.31E-05	0.01131	up
boy_ICSI-ET_vs_boy_ctrl	*RABL5*	64792	1.25961895	5.32E-05	0.01131	up
boy_ICSI-ET_vs_boy_ctrl	*PKP4*	8502	-1.1417757	5.53E-05	0.011635	down

Supplementary Table S4 (continued)

boy_ICSI-ET_vs_boy_ctrl	*F11R*	50848	1.41085589	6.17E-05	0.012865	up
boy_ICSI-ET_vs_boy_ctrl	*IL7*	3574	1.48072855	6.34E-05	0.012946	up
boy_ICSI-ET_vs_boy_ctrl	*ZC2HC1A*	51101	1.48072875	6.34E-05	0.012946	up
boy_ICSI-ET_vs_boy_ctrl	*TMEM132D*	121256	1.60016233	6.41E-05	0.012946	up
boy_ICSI-ET_vs_boy_ctrl	*USO1*	8615	1.34360642	6.53E-05	0.01307	up
boy_ICSI-ET_vs_boy_ctrl	*NES*	10763	1.5022062	6.62E-05	0.013127	up
boy_ICSI-ET_vs_boy_ctrl	*ZFP90*	146198	2.06137336	6.78E-05	0.01327	up
boy_ICSI-ET_vs_boy_ctrl	*SLC13A2*	9058	2.21658236	6.83E-05	0.01327	up
boy_ICSI-ET_vs_boy_ctrl	*ZNF626*	199777	1.99380762	7.02E-05	0.013517	up
boy_ICSI-ET_vs_boy_ctrl	*ZNF175*	7728	2.05052308	7.45E-05	0.014213	up
boy_ICSI-ET_vs_boy_ctrl	*ZNF625-ZNF20*	100529855	2.02461543	7.71E-05	0.01457	up
boy_ICSI-ET_vs_boy_ctrl	*ZNF416*	55659	2.05849861	7.92E-05	0.014819	up
boy_ICSI-ET_vs_boy_ctrl	*B4GALNT4*	338707	2.01165118	8.05E-05	0.014929	up
boy_ICSI-ET_vs_boy_ctrl	*TMPRSS13*	84000	1.45368427	8.62E-05	0.015843	up
boy_ICSI-ET_vs_boy_ctrl	*PCLO*	27445	1.43351736	9.36E-05	0.01704	up
boy_ICSI-ET_vs_boy_ctrl	*ZNF652*	22834	1.48504003	9.72E-05	0.017545	up
boy_ICSI-ET_vs_boy_ctrl	*ZNF576*	79177	2.13051459	0.00010154	0.018163	up
boy_ICSI-ET_vs_boy_ctrl	*GALNT7*	51809	1.47123718	0.00010471	0.018558	up
boy_ICSI-ET_vs_boy_ctrl	*TUB*	7275	1.51300778	0.00010742	0.018558	up
boy_ICSI-ET_vs_boy_ctrl	*AL354993.1*	NA	1.33909458	0.00011684	0.019867	up
boy_ICSI-ET_vs_boy_ctrl	*TRAPPC2P1*	10597	2.01362971	0.00011697	0.019867	up
boy_ICSI-ET_vs_boy_ctrl	*ALKBH6*	84964	2.02949416	0.00011859	0.019975	up
boy_ICSI-ET_vs_boy_ctrl	*RCBTB2*	1102	-1.0624622	0.00012095	0.020087	down
boy_ICSI-ET_vs_boy_ctrl	*FGFR3*	2261	1.31655848	0.00012124	0.020087	up
boy_ICSI-ET_vs_boy_ctrl	*TDRD10*	126668	1.0872039	0.00013582	0.022321	up
boy_ICSI-ET_vs_boy_ctrl	*TRAPPC10*	7109	-1.2776805	0.00014616	0.023825	down
boy_ICSI-ET_vs_boy_ctrl	*SNRPN*	6638	1.84488884	0.00014832	0.023984	up
boy_ICSI-ET_vs_boy_ctrl	*PPM1H*	57460	1.44681433	0.00015025	0.024104	up
boy_ICSI-ET_vs_boy_ctrl	*KCNK4*	50801	1.08730489	0.00015149	0.02411	up
boy_ICSI-ET_vs_boy_ctrl	*SLC25A16*	8034	1.24666965	0.00016525	0.026028	up
boy_ICSI-ET_vs_boy_ctrl	*SLC25A37*	51312	1.35136796	0.00016611	0.026028	up
boy_ICSI-ET_vs_boy_ctrl	*TPPP3*	51673	1.62947348	0.00017611	0.027382	up
boy_ICSI-ET_vs_boy_ctrl	*COL9A2*	1298	1.36321917	0.00017897	0.027615	up
boy_ICSI-ET_vs_boy_ctrl	*SLC25A21*	89874	1.33557364	0.00018266	0.027672	up
boy_ICSI-ET_vs_boy_ctrl	*ZAP70*	7535	1.6624319	0.00018934	0.027672	up
boy_ICSI-ET_vs_boy_ctrl	*VGLL3*	389136	1.40765968	0.00019009	0.027672	up
boy_ICSI-ET_vs_boy_ctrl	*RP4-583P15.14*	NA	1.15666074	0.00019089	0.027672	up
boy_ICSI-ET_vs_boy_ctrl	*LIME1*	54923	1.15666131	0.00019107	0.027672	up
boy_ICSI-ET_vs_boy_ctrl	*RP4-583P15.15*	NA	1.15666173	0.0001912	0.027672	up
boy_ICSI-ET_vs_boy_ctrl	*ZGPAT*	84619	1.15666215	0.00019133	0.027672	up
boy_ICSI-ET_vs_boy_ctrl	*ELAVL2*	1993	1.58802576	0.00019167	0.027672	up
boy_ICSI-ET_vs_boy_ctrl	*ZNF701*	55762	1.73975797	0.00019842	0.028444	up
boy_ICSI-ET_vs_boy_ctrl	*CCDC71*	64925	1.99512986	0.00021233	0.030224	up
boy_ICSI-ET_vs_boy_ctrl	*ZYX*	7791	3.51397556	0.00021443	0.030309	up
boy_ICSI-ET_vs_boy_ctrl	*DKK3*	27122	1.28582473	0.00021942	0.030799	up
boy_ICSI-ET_vs_boy_ctrl	*SCUBE2*	57758	1.47839621	0.00022131	0.03085	up
boy_ICSI-ET_vs_boy_ctrl	*CREB1*	1385	1.68425417	0.00022646	0.031271	up
boy_ICSI-ET_vs_boy_ctrl	*CDRT4*	284040	1.82786341	0.00022897	0.031271	up
boy_ICSI-ET_vs_boy_ctrl	*ZNF160*	90338	1.80221097	0.0002326	0.031554	up

Supplementary Table S4 (continued)

boy_ICSI-ET_vs_boy_ctrl	*ZNF880*	400713	1.90377897	0.00023431	0.031574	up
boy_ICSI-ET_vs_boy_ctrl	*C5orf38*	153571	1.81682799	0.00024104	0.032146	up
boy_ICSI-ET_vs_boy_ctrl	*DNAJC5G*	285126	1.24125916	0.00024174	0.032146	up
boy_ICSI-ET_vs_boy_ctrl	*RPF1*	80135	-1.0596504	0.00024348	0.032166	down
boy_ICSI-ET_vs_boy_ctrl	*SDHAF1*	644096	2.06398483	0.00024656	0.032276	up
boy_ICSI-ET_vs_boy_ctrl	*TMEM132E*	124842	1.75053448	0.00024751	0.032276	up
boy_ICSI-ET_vs_boy_ctrl	*TVP23C*	201158	1.88906338	0.00025146	0.032424	up
boy_ICSI-ET_vs_boy_ctrl	*IRX2*	153572	1.87497088	0.00025185	0.032424	up
boy_ICSI-ET_vs_boy_ctrl	*ZNF613*	79898	1.8552276	0.0002553	0.03248	up
boy_ICSI-ET_vs_boy_ctrl	*BCS1L*	617	1.84221624	0.00025549	0.03248	up
boy_ICSI-ET_vs_boy_ctrl	*XPO7*	23039	1.20891338	0.00026009	0.032857	up
boy_ICSI-ET_vs_boy_ctrl	*WASH4P*	374677	1.86163323	0.00026503	0.033068	up
boy_ICSI-ET_vs_boy_ctrl	*C1orf110*	339512	1.75009376	0.00027059	0.033555	up
boy_ICSI-ET_vs_boy_ctrl	*APLP1*	333	1.90789728	0.00027395	0.033754	up
boy_ICSI-ET_vs_boy_ctrl	*KSR1*	8844	1.21178422	0.00027978	0.034067	up
boy_ICSI-ET_vs_boy_ctrl	*PODNL1*	79883	1.47707719	0.00028249	0.034139	up
boy_ICSI-ET_vs_boy_ctrl	*TWIST1*	7291	1.18946323	0.00028527	0.034139	up
boy_ICSI-ET_vs_boy_ctrl	*KBTBD4*	55709	1.64706654	0.00028544	0.034139	up
boy_ICSI-ET_vs_boy_ctrl	*DCLK3*	85443	1.40539793	0.00028857	0.034311	up
boy_ICSI-ET_vs_boy_ctrl	*IRX1*	79192	1.52871115	0.00029981	0.035246	up
boy_ICSI-ET_vs_boy_ctrl	*ADAM33*	80332	1.6817225	0.00030185	0.035268	up
boy_ICSI-ET_vs_boy_ctrl	*UBAC2*	337867	1.05710843	0.0003066	0.035617	up
boy_ICSI-ET_vs_boy_ctrl	*KLHL17*	339451	1.90417955	0.00031699	0.036405	up
boy_ICSI-ET_vs_boy_ctrl	*MCMBP*	79892	1.35167221	0.00032214	0.036551	up
boy_ICSI-ET_vs_boy_ctrl	*SLC7A2*	6542	1.58275449	0.00032528	0.036551	up
boy_ICSI-ET_vs_boy_ctrl	*PCDH7*	5099	1.30105316	0.00032733	0.036551	up
boy_ICSI-ET_vs_boy_ctrl	*TVP23C-CDRT4*	100533496	1.79825192	0.00032889	0.036551	up
boy_ICSI-ET_vs_boy_ctrl	*PDGFRL*	5157	1.42347623	0.00032911	0.036551	up
boy_ICSI-ET_vs_boy_ctrl	*ACTN4*	81	1.70389456	0.00033414	0.036907	up
boy_ICSI-ET_vs_boy_ctrl	*LGI2*	55203	1.59477146	0.00033795	0.037125	up
boy_ICSI-ET_vs_boy_ctrl	*CIZ1*	25792	1.21675163	0.00036295	0.03961	up
boy_ICSI-ET_vs_boy_ctrl	*SHMT2*	6472	1.02023749	0.00036813	0.03961	up
boy_ICSI-ET_vs_boy_ctrl	*UTS2*	10911	-1.5957309	0.00036841	0.03961	down
boy_ICSI-ET_vs_boy_ctrl	*UBE3D*	90025	1.41093922	0.00038078	0.040531	up
boy_ICSI-ET_vs_boy_ctrl	*LRRC9*	341883	1.18122166	0.00038099	0.040531	up
boy_ICSI-ET_vs_boy_ctrl	*CRABP2*	1382	1.7785691	0.00038709	0.040964	up
boy_ICSI-ET_vs_boy_ctrl	*UBL5*	59286	1.40058559	0.00041081	0.043047	up
boy_ICSI-ET_vs_boy_ctrl	*FBXL12*	54850	1.40058595	0.00041103	0.043047	up
boy_ICSI-ET_vs_boy_ctrl	*PRKD3*	23683	-1.1936396	0.00041337	0.04307	down
boy_ICSI-ET_vs_boy_ctrl	*ZNF829*	374899	1.77587532	0.00042467	0.04402	up
boy_ICSI-ET_vs_boy_ctrl	*SYT2*	127833	1.27458525	0.00042982	0.044326	up
boy_ICSI-ET_vs_boy_ctrl	*SIGLEC8*	27181	1.88186082	0.00043621	0.044757	up
boy_ICSI-ET_vs_boy_ctrl	*C9orf53*	51198	1.16302755	0.00045408	0.045478	up
boy_ICSI-ET_vs_boy_ctrl	*FAM150B*	285016	1.18981365	0.00045587	0.045478	up
boy_ICSI-ET_vs_boy_ctrl	*HIPK3*	10114	1.58933105	0.00045706	0.045478	up
boy_ICSI-ET_vs_boy_ctrl	*MOGAT2*	80168	1.26228182	0.00045889	0.045478	up
boy_ICSI-ET_vs_boy_ctrl	*PTPMT1*	114971	1.44010214	0.0004608	0.045478	up
boy_ICSI-ET_vs_boy_ctrl	*NDUFS3*	4722	1.44010264	0.00046094	0.045478	up
boy_ICSI-ET_vs_boy_ctrl	*SIPA1L1*	26037	1.32379762	0.00046124	0.045478	up

Supplementary Table S4 (continued)

boy_ICSI-ET_vs_boy_ctrl	*CIB4*	130106	1.18322791	0.00046999	0.046116	up
boy_ICSI-ET_vs_boy_ctrl	*ZNF214*	7761	1.63137814	0.00048516	0.047375	up
boy_ICSI-ET_vs_boy_ctrl	*FRMPD2*	143162	1.24894577	0.00049466	0.04807	up
boy_ICSI-ET_vs_boy_ctrl	*KIRREL2*	84063	1.90167936	0.0005046	0.048802	up
boy_ICSI-ET_vs_boy_ctrl	*EHD1*	10938	1.43435451	0.00050774	0.048871	up
**boy_IVF-ET_vs_boy_ctrl**						
boy_IVF-ET_vs_boy_ctrl	*DNAJB14*	79982	-2.8848109	1.31E-07	0.002642	down
boy_IVF-ET_vs_boy_ctrl	*TRIM16L*	147166	-1.1160228	5.21E-07	0.005264	down
**girl_ICSI-ET_vs_girl_ctrl**						
girl_ICSI-ET_vs_girl_ctrl	*DAXX*	1616	8.27227009	3.17E-08	0.000406	up
girl_ICSI-ET_vs_girl_ctrl	*AC008132.1*	NA	3.99784422	4.60E-08	0.000406	up
girl_ICSI-ET_vs_girl_ctrl	*SYNGAP1*	8831	5.21917778	6.02E-08	0.000406	up
girl_ICSI-ET_vs_girl_ctrl	*C20orf112*	140688	4.44985493	1.55E-07	0.000785	up
girl_ICSI-ET_vs_girl_ctrl	*EGFR*	1956	1.37831816	4.04E-07	0.001631	up
girl_ICSI-ET_vs_girl_ctrl	*KSR1*	8844	3.91267179	8.82E-07	0.002971	up
girl_ICSI-ET_vs_girl_ctrl	*PRB1*	5542	-1.7047488	1.72E-06	0.004967	down
girl_ICSI-ET_vs_girl_ctrl	*ZBTB22*	9278	7.03523507	2.19E-06	0.005542	up
girl_ICSI-ET_vs_girl_ctrl	*RND3*	390	-1.9885559	3.60E-06	0.008082	down
girl_ICSI-ET_vs_girl_ctrl	*FLYWCH2*	114984	-2.2629414	5.90E-06	0.011923	down
girl_ICSI-ET_vs_girl_ctrl	*RP11-410N8.4*	NA	3.7040231	8.43E-06	0.013978	up
girl_ICSI-ET_vs_girl_ctrl	*SLC12A1*	6557	-1.8672576	1.21E-05	0.013978	down
girl_ICSI-ET_vs_girl_ctrl	*LAMC2*	3918	1.35187385	1.28E-05	0.013978	up
girl_ICSI-ET_vs_girl_ctrl	*POU2F2*	5452	-2.0118085	1.39E-05	0.013978	down
girl_ICSI-ET_vs_girl_ctrl	*MDC1*	9656	6.45373712	1.53E-05	0.013978	up
girl_ICSI-ET_vs_girl_ctrl	*ART4*	420	-1.5767518	1.80E-05	0.014387	down
girl_ICSI-ET_vs_girl_ctrl	*NEDD8-MDP1*	100528064	1.66971277	2.70E-05	0.014387	up
girl_ICSI-ET_vs_girl_ctrl	*NEDD8*	4738	1.6697121	2.70E-05	0.014387	up
girl_ICSI-ET_vs_girl_ctrl	*CROCC*	9696	-1.0767357	2.89E-05	0.014387	down
girl_ICSI-ET_vs_girl_ctrl	*PCED1A*	64773	1.33527261	2.93E-05	0.014387	up
girl_ICSI-ET_vs_girl_ctrl	*IGSF6*	10261	-1.0037654	3.36E-05	0.0153	down
girl_ICSI-ET_vs_girl_ctrl	*REPS2*	9185	-1.6544346	3.41E-05	0.0153	down
girl_ICSI-ET_vs_girl_ctrl	*AJAP1*	55966	-2.1244098	3.45E-05	0.0153	down
girl_ICSI-ET_vs_girl_ctrl	*HLA-DRB5*	3127	-4.6328422	3.67E-05	0.0153	down
girl_ICSI-ET_vs_girl_ctrl	*ACBD6*	84320	-1.5641958	4.15E-05	0.0153	down
girl_ICSI-ET_vs_girl_ctrl	*UNC119*	9094	3.33344709	4.53E-05	0.0153	up
girl_ICSI-ET_vs_girl_ctrl	*NOVA2*	4858	1.88028236	4.58E-05	0.0153	up
girl_ICSI-ET_vs_girl_ctrl	*SMN2*	6607	4.78688491	5.68E-05	0.015319	up
girl_ICSI-ET_vs_girl_ctrl	*SIGLEC9*	27180	-1.0740075	6.53E-05	0.015573	down
girl_ICSI-ET_vs_girl_ctrl	*PRODH2*	58510	3.72580246	9.03E-05	0.01636	up
girl_ICSI-ET_vs_girl_ctrl	*PRX*	57716	2.05299622	9.48E-05	0.016435	up
girl_ICSI-ET_vs_girl_ctrl	*LAMA1*	284217	1.21577144	0.00010963	0.017609	up
girl_ICSI-ET_vs_girl_ctrl	*CEP70*	80321	-1.3295815	0.00011092	0.017609	down
girl_ICSI-ET_vs_girl_ctrl	*HOXA11*	3207	1.59487769	0.00011251	0.017629	up
girl_ICSI-ET_vs_girl_ctrl	*RIPK4*	54101	-1.6649551	0.00012021	0.018472	down
girl_ICSI-ET_vs_girl_ctrl	*TBCEL*	219899	1.30608148	0.00013446	0.019746	up
girl_ICSI-ET_vs_girl_ctrl	*CACNA1E*	777	-1.2301598	0.00014201	0.020504	down
girl_ICSI-ET_vs_girl_ctrl	*GCDH*	2639	1.17372632	0.00014779	0.0206	up
girl_ICSI-ET_vs_girl_ctrl	*SYCE2*	256126	1.17372625	0.00014786	0.0206	up
girl_ICSI-ET_vs_girl_ctrl	*CBR4*	84869	1.38687763	0.00015112	0.020779	up

Supplementary Table S4 (continued)

girl_ICSI-ET_vs_girl_ctrl	*ZNF701*	55762	1.54502183	0.0001557	0.020985	up
girl_ICSI-ET_vs_girl_ctrl	*CHIT1*	1118	-1.6408333	0.00016609	0.020985	down
girl_ICSI-ET_vs_girl_ctrl	*RNMTL1*	55178	-1.0730152	0.00018559	0.021974	down
girl_ICSI-ET_vs_girl_ctrl	*THYN1*	29087	1.16448061	0.00018908	0.021974	up
girl_ICSI-ET_vs_girl_ctrl	*ACAD8*	27034	1.16448036	0.00018916	0.021974	up
girl_ICSI-ET_vs_girl_ctrl	*TSC22D2*	9819	1.37983422	0.00020802	0.022851	up
girl_ICSI-ET_vs_girl_ctrl	*SLC25A37*	51312	1.11517186	0.00022221	0.023641	up
girl_ICSI-ET_vs_girl_ctrl	*AKAP7*	9465	-1.6197997	0.00023463	0.023999	down
girl_ICSI-ET_vs_girl_ctrl	*AXL*	558	-1.6500437	0.00024938	0.024469	down
girl_ICSI-ET_vs_girl_ctrl	*CEP97*	79598	1.31080438	0.00025441	0.024543	up
girl_ICSI-ET_vs_girl_ctrl	*HNF1A*	6927	-1.8939073	0.00025719	0.024543	down
girl_ICSI-ET_vs_girl_ctrl	*CLU*	1191	1.35304112	0.00026063	0.024543	up
girl_ICSI-ET_vs_girl_ctrl	*GPRIN1*	114787	1.68253748	0.00026477	0.024549	up
girl_ICSI-ET_vs_girl_ctrl	*HNRNPUL1*	11100	-1.5414583	0.00028461	0.024901	down
girl_ICSI-ET_vs_girl_ctrl	*PIP4K2B*	8396	-3.7044092	0.00032456	0.026453	down
girl_ICSI-ET_vs_girl_ctrl	*KIDINS220*	57498	-1.4032351	0.00033254	0.026598	down
girl_ICSI-ET_vs_girl_ctrl	*CNBD1*	168975	1.10527788	0.00033914	0.026598	up
girl_ICSI-ET_vs_girl_ctrl	*LRG1*	116844	-1.3350837	0.00039109	0.028334	down
girl_ICSI-ET_vs_girl_ctrl	*MUC8*	100129528	2.19089508	0.00043133	0.030262	up
girl_ICSI-ET_vs_girl_ctrl	*GPN3*	51184	1.21757861	0.00046419	0.03138	up
girl_ICSI-ET_vs_girl_ctrl	*TLR10*	81793	-1.018656	0.0004797	0.03179	down
girl_ICSI-ET_vs_girl_ctrl	*CDC25A*	993	-1.1575292	0.00051343	0.033585	down
girl_ICSI-ET_vs_girl_ctrl	*ZSCAN2*	54993	1.17176828	0.00064619	0.03808	up
girl_ICSI-ET_vs_girl_ctrl	*VCPIP1*	80124	1.38457825	0.00066524	0.038751	up
girl_ICSI-ET_vs_girl_ctrl	*ATG5*	9474	-1.1792187	0.00067734	0.038898	down
girl_ICSI-ET_vs_girl_ctrl	*NOS2*	4843	2.98132414	0.00069066	0.039214	up
girl_ICSI-ET_vs_girl_ctrl	*TMEM87B*	84910	-1.0210324	0.00071485	0.040026	down
girl_ICSI-ET_vs_girl_ctrl	*MRPS7*	51081	-2.0357772	0.00072652	0.040033	down
girl_ICSI-ET_vs_girl_ctrl	*SGK494*	124923	2.71894543	0.00072884	0.040033	up
girl_ICSI-ET_vs_girl_ctrl	*NKX2-6*	137814	-1.1874509	0.00073715	0.040301	down
girl_ICSI-ET_vs_girl_ctrl	*U2AF1L4*	199746	2.25505949	0.00081144	0.041196	up
girl_ICSI-ET_vs_girl_ctrl	*CPA1*	1357	-1.2314296	0.00081188	0.041196	down
girl_ICSI-ET_vs_girl_ctrl	*PSENEN*	55851	2.26368094	0.00082079	0.041373	up
girl_ICSI-ET_vs_girl_ctrl	*ABCC3*	8714	1.03346488	0.00088199	0.043271	up
girl_ICSI-ET_vs_girl_ctrl	*USP10*	9100	-1.4768489	0.00089335	0.04359	down
girl_ICSI-ET_vs_girl_ctrl	*ACMSD*	130013	1.00648588	0.00094244	0.045249	up
girl_ICSI-ET_vs_girl_ctrl	*MIF4GD*	57409	-1.6830075	0.00101865	0.047237	down
girl_ICSI-ET_vs_girl_ctrl	*CNIH3*	149111	1.18382995	0.00108852	0.049188	up
girl_ICSI-ET_vs_girl_ctrl	*CD300C*	10871	1.41865142	0.00111536	0.049376	up
**girl_IVF-ET_vs_girl_ctrl**						
girl_IVF-ET_vs_girl_ctrl	*HLA-DOA*	3111	-7.9621569	6.05E-07	0.01222	down
girl_IVF-ET_vs_girl_ctrl	*SAPCD1*	401251	-8.168067	1.91E-06	0.019277	down
girl_IVF-ET_vs_girl_ctrl	*TRIM40*	135644	-4.3952872	4.04E-06	0.027197	down
girl_IVF-ET_vs_girl_ctrl	*MSH5*	4439	-6.9173524	9.40E-06	0.038	down
girl_IVF-ET_vs_girl_ctrl	*MSH5-SAPCD1*	100532732	-6.9173524	9.40E-06	0.038	down

This table showed the comparison results of each gene promoter H3K4me3 read counts between natural conception-boys (n=6) vs ICSI-boys (n=2), natural conception-boys (n=6) vs IVF-boys (n=4), natural conception-girls (n=6) vs ICSI-girls (n=4), and natural conception-girls (n=6) vs IVF-girls (n=4). The comparison was performed via R package ‘edgeR’. The genes with |log FC| >1 and FDR < 0.05 were selected as “genes with deH3K4me3”, compared with the same-gender natural conception groups. FC, fold change; FDR, false discovery rate; IVF, in vitro fertilization.

Supplementary Table S5. Cardiovascular-disease-associated genes with deH3K4me3 in ICSI children

Group	Term	entrezID	logFC	*P*-Value	FDR	up_down
boy_ICSI-ET_vs_boy_ctrl	*AGER*	177	6.342532279	9.31E-10	1.71E-06	up
boy_ICSI-ET_vs_boy_ctrl	*C5orf60*	285679	2.628473399	8.92E-07	0.000450877	up
boy_ICSI-ET_vs_boy_ctrl	*TIMP2*	7077	1.812965347	2.71E-06	0.00111772	up
boy_ICSI-ET_vs_boy_ctrl	*DNAH9*	1770	1.9723031	4.76E-06	0.001658116	up
boy_ICSI-ET_vs_boy_ctrl	*COX7A1*	1346	2.342242319	1.39E-05	0.004178111	up
boy_ICSI-ET_vs_boy_ctrl	*SLN*	6588	1.32924614	1.43E-05	0.004178111	up
boy_ICSI-ET_vs_boy_ctrl	*DGCR6*	8214	3.106559485	2.61E-05	0.006839907	up
boy_ICSI-ET_vs_boy_ctrl	*IRF2BPL*	64207	2.11364975	3.45E-05	0.008499563	up
boy_ICSI-ET_vs_boy_ctrl	*C1orf159*	54991	1.36680443	3.80E-05	0.008938189	up
boy_ICSI-ET_vs_boy_ctrl	*F11R*	50848	1.410855893	6.17E-05	0.012864849	up
boy_ICSI-ET_vs_boy_ctrl	*NES*	10763	1.502206201	6.62E-05	0.013127228	up
boy_ICSI-ET_vs_boy_ctrl	*FGFR3*	2261	1.316558477	0.00012124	0.020086868	up
boy_ICSI-ET_vs_boy_ctrl	*TRAPPC10*	7109	-1.27768052	0.00014616	0.023825293	down
boy_ICSI-ET_vs_boy_ctrl	*DKK3*	27122	1.285824729	0.00021942	0.030799247	up
boy_ICSI-ET_vs_boy_ctrl	*SCUBE2*	57758	1.478396211	0.00022131	0.030850308	up
boy_ICSI-ET_vs_boy_ctrl	*CREB1*	1385	1.684254169	0.00022646	0.031270979	up
boy_ICSI-ET_vs_boy_ctrl	*SDHAF1*	644096	2.063984826	0.00024656	0.03227641	up
boy_ICSI-ET_vs_boy_ctrl	*BCS1L*	617	1.842216243	0.00025549	0.03247991	up
boy_ICSI-ET_vs_boy_ctrl	*XPO7*	23039	1.208913378	0.00026009	0.032857326	up
boy_ICSI-ET_vs_boy_ctrl	*TWIST1*	7291	1.189463226	0.00028527	0.034139243	up
boy_ICSI-ET_vs_boy_ctrl	*SHMT2*	6472	1.02023749	0.00036813	0.039609656	up
boy_ICSI-ET_vs_boy_ctrl	*UTS2*	10911	-1.59573088	0.00036841	0.039609656	down
boy_ICSI-ET_vs_boy_ctrl	*NDUFS3*	4722	1.440102645	0.00046094	0.045478354	up
boy_ICSI-ET_vs_boy_ctrl	*SIPA1L1*	26037	1.323797625	0.00046124	0.045478354	up
girl_ICSI-ET_vs_girl_ctrl	*DAXX*	1616	8.272270087	3.17E-08	0.000405905	up
girl_ICSI-ET_vs_girl_ctrl	*SYNGAP1*	8831	5.219177778	6.02E-08	0.000405905	up
girl_ICSI-ET_vs_girl_ctrl	*EGFR*	1956	1.378318157	4.04E-07	0.001631279	up
girl_ICSI-ET_vs_girl_ctrl	*SLC12A1*	6557	-1.86725762	1.21E-05	0.013977922	down
girl_ICSI-ET_vs_girl_ctrl	*CACNA1E*	777	-1.23015984	0.00014201	0.020503788	down
girl_ICSI-ET_vs_girl_ctrl	*HNF1A*	6927	-1.89390727	0.00025719	0.024542722	down
girl_ICSI-ET_vs_girl_ctrl	*CLU*	1191	1.353041117	0.00026063	0.024542722	up
girl_ICSI-ET_vs_girl_ctrl	*GPRIN1*	114787	1.682537484	0.00026477	0.024549092	up
girl_ICSI-ET_vs_girl_ctrl	*ATG5*	9474	-1.17921866	0.00067734	0.038898426	down
girl_ICSI-ET_vs_girl_ctrl	*NOS2*	4843	2.981324137	0.00069066	0.039214454	up
girl_ICSI-ET_vs_girl_ctrl	*NKX2-6*	137814	-1.18745086	0.00073715	0.040301159	down
girl_ICSI-ET_vs_girl_ctrl	*USP10*	9100	-1.47684886	0.00089335	0.043590481	down

This table showed the overlapping results of cardiovascular-disease-associated genes and the genes with deH3K4me3 in ICSI children.

Supplementary Table S6. Transcription factor analysis for genes with H3K4me3 enrichment in 4-cell, 8-cell, and ICM stages

motif	NES	AUC	TF_highConf	nEnrGenes	rankAtMax
		**4 cell**			
tfdimers__MD00344	6.83	0.157	HOXA13	5	303
dbcorrdb__ZNF274__ENCSR000EVR_1__m	5.96	0.139	ZNF274 (directAnnotation).	9	2689
dbcorrdb__ZNF274__ENCSR000EVG_1__m	5.75	0.134	ZNF274 (directAnnotation).	8	1794
transfac_pro__M05282	5.69	0.133		4	399
transfac_pro__M05883	5.26	0.124	ZNF709 (directAnnotation).	3	57
predrem__nrMotif431	5.21	0.123		3	66
transfac_pro__M06894	5.14	0.122	ZFP90 (inferredBy_Orthology).	4	338
dbcorrdb__CHD1__ENCSR000AQK_1__m1	5.09	0.121	CHD1 (directAnnotation).	3	87
predrem__nrMotif621	4.87	0.116		5	598
dbcorrdb__eGFP-NR4A1__ENCSR000DJW_1__m1	4.74	0.114		4	465
taipale_cyt_meth__ZNF524_NYTCGNACCCKN_FL_repr	4.72	0.113	ZNF524 (directAnnotation).	3	152
cisbp__M4839	4.67	0.112	KLF17; KLF18 (inferredBy_Orthology).	5	577
dbcorrdb__ZNF274__ENCSR000EVR_1__m	4.66	0.112	ZNF274 (directAnnotation).	3	188
elemento__CTCGCCC	4.61	0.111		3	161
tiffin__TIFDMEM0000047	4.53	0.109		4	614
dbcorrdb__STAT5A__ENCSR000BQZ_1__m	4.43	0.107	STAT5A (directAnnotation).	4	577
flyfactorsurvey__CG3065_F1-5_SOLEXA_2.5_FBgn0034946	4.33	0.105	KLF17; KLF18 (inferredBy_Orthology).	5	580
transfac_pro__M05881	4.31	0.105	ZNF676 (directAnnotation).	3	338
neph__UW.Motif.0020	4.3	0.105		3	315
transfac_pro__M06415	4.3	0.105	ZNF429 (directAnnotation).	3	295
taipale_cyt_meth__PAX3_NSGTCACGSNNATTAN_eDBD_meth	4.28	0.104	PAX3 (directAnnotation).	3	283
transfac_pro__M07628	4.27	0.104	TFAP2A; TFAP2B; TFAP2C; TFAP2D; TFAP2E	5	652
taipale_tf_pairs__GCM1_PITX1_GGATTANNNNNNNNNTGCGGG_CAP_repr	4.24	0.103	GCM1; PITX1 (directAnnotation).	3	307
elemento__AATCCCAGC	4.23	0.103		7	1794
swissregulon__hs__EN1_2.p2	4.2	0.102	EN1; EN2 (directAnnotation).	3	188
cisbp__M5784	4.19	0.102	RHOXF1	3	286
taipale_tf_pairs__CEBPG_ATF4_NNATGAYGCAAT_CAP	4.18	0.102	ATF4; CEBPG (directAnnotation).	3	240
neph__UW.Motif.0327	4.17	0.102		3	223
transfac_pro__M01785	4.14	0.101		8	3777
tfdimers__MD00209	4.13	0.101	MZF1; TBP (directAnnotation).	3	228
dbcorrdb__ZNF274__ENCSR000EWY_1__m6	4.12	0.101	ZNF274 (directAnnotation).	3	338
tfdimers__MD00160	4.12	0.101	CRX; RAX (directAnnotation).	3	181
taipale_cyt_meth__PAX7_NSGTCACGSNNRTTAN_FL_meth	4.1	0.1	PAX7 (directAnnotation).	3	292
cisbp__M0032	4.09	0.1		3	252
transfac_pro__M06968	4.06	0.0997	ZNF467 (inferredBy_Orthology).	9	4533

Supplementary Table S6 (continued)

taipale_cyt_meth__KLF15_NCCMCGCCCMYN_FL_meth	4.06	0.0996	KLF15 (directAnnotation).	5	690
cisbp__M5522	4.05	0.0994		3	252
flyfactorsurvey__CG3065_F1-3_SANGER_2.5_FBgn0034946	4.04	0.0993	KLF17; KLF18 (inferredBy_Orthology).	6	1794
elemento__CCTCCTCC	4.03	0.0991		5	1441
jaspar__MA0971.1	4.03	0.099		3	262
cisbp__M6400	4.02	0.0988	OTX1 (directAnnotation).	4	434
taipale_cyt_meth__KLF13_NRCCACGCCCMYN_FL_meth	4.02	0.0988	KLF13 (directAnnotation).	5	783
taipale__HNF1B_full_GTTAATNATTAAY	4.02	0.0987	HNF1B (directAnnotation).	3	243
transfac_pro__M00684	4.01	0.0987		3	299
predrem__nrMotif814	4.01	0.0987		4	931
taipale__RHOXF1_full_GGMTNAKCC_repr	4	0.0985	RHOXF1 [illegible]	9	4993
transfac_pro__M05224	4	0.0984		2	38
cisbp__M4761	4	0.0984		3	240
taipale_tf_pairs__ATF4_CEBPD_NGATGATGCAATNN_CAP	3.99	0.0982	ATF4; CEBPD (directAnnotation).	5	928
taipale_tf_pairs__ATF4_CEBPB_NNATGAYGCAAYN_CAP	3.99	0.0981	ATF4; CEBPB (directAnnotation).	3	228
taipale__KLF16_DBD_GCCMCGCCCMC_repr	3.98	0.0981	KLF16 (directAnnotation).	3	310
predrem__nrMotif433	3.96	0.0975		3	211
predrem__nrMotif1028	3.95	0.0974		3	280
predrem__nrMotif366	3.93	0.097		3	356
transfac_pro__M09272	3.92	0.0967		3	243
homer__CCTGTCAATCAN_Pbx3	3.91	0.0966	PBX3 (directAnnotation).	5	849
swissregulon__sacCer__TBF1	3.91	0.0965		7	2428
transfac_pro__M05192	3.91	0.0964		5	1331
transfac_pro__M05136	3.9	0.0963		3	399
taipale__MGA_DBD_AGGTGTKANNTMACACCT_repr	3.89	0.0961	MGA (directAnnotation).	3	247
hocomoco__OTX1_HUMAN.H11MO.0.D	3.88	0.0959	OTX1 (directAnnotation).	4	601
cisbp__M4457	3.84	0.0952	CTCF (directAnnotation).	3	399
predrem__nrMotif369	3.84	0.0952		3	338
taipale_cyt_meth__TFAP2B_NGCCNNNGGCN_eDBD	3.84	0.0951	TFAP2B (directAnnotation).	6	774
cisbp__M5741	3.84	0.0951	POU4F2 (directAnnotation).	3	277
cisbp__M4837	3.84	0.095	KLF17; KLF18 (inferredBy_Orthology).	3	399
transfac_pro__M04937	3.82	0.0946	TCF12 (directAnnotation).	3	338
predrem__nrMotif2659	3.82	0.0946		7	1264
jaspar__MA0558.1	3.81	0.0945		3	440
transfac_pro__M06067	3.81	0.0944	ZNF195 (directAnnotation).	2	7
taipale_cyt_meth__KLF13_NRCCACGCCCMYN_FL_repr	3.79	0.0942	KLF13 (directAnnotation).	5	789
cisbp__M4586	3.78	0.0939	CTCF (directAnnotation).	3	360
transfac_pro__M07038	3.77	0.0936	DBP (directAnnotation).	4	577
taipale_cyt_meth__MAFA_NWWWNTGCTGACN_eDBD	3.76	0.0935	MAFA (directAnnotation).	2	35
taipale__TBX15_DBD_AGGTGTGA	3.75	0.0933	TBX15 (directAnnotation).	4	577
cisbp__M4584	3.75	0.0933	CTCF (directAnnotation).	3	305
cisbp__M5209	3.74	0.093	SP6; SP7; SP8; SP9 (inferredBy_Orthology).	3	296

Supplementary Table S6 (continued)

taipale__POU4F2_DBD_NTGMATAATTAAT KAG	3.72	0.0927	POU4F2 (directAnnotation).	3	304
taipale_cyt_meth__PAX7_NSGTCACGSNNR TTAN_FL	3.72	0.0927	PAX7 (directAnnotation).	3	444
taipale_cyt_meth__KLF13_NRCCACGCCCM YN_eDBD	3.72	0.0927	KLF13 (directAnnotation).	5	928
fantom__motif109_TCNMTMGC	3.71	0.0924		3	399
dbcorrdb__JUN__ENCSR000EGH_1__m2	3.7	0.0923	JUN (directAnnotation).	5	1264
elemento__CCTCGCC	3.7	0.0923		3	399
transfac_pro__M04880	3.69	0.092	TBP (directAnnotation).	3	434
taipale_cyt_meth__GLIS1_NACCCCCCACG WMGN_eDBD	3.67	0.0916	GLIS1 (directAnnotation).	3	434
taipale_cyt_meth__SP3_NRCCMCGCCCMY N_eDBD	3.66	0.0914	SP3 (directAnnotation).	4	614
taipale__HNF1B_full_NRTTAATNATTAACN	3.65	0.0911	HNF1B (directAnnotation).	3	371
taipale_cyt_meth__ZBTB12_NGCTGNNCCG CGYN_eDBD_meth	3.64	0.0911	ZBTB12 (directAnnotation).	3	399
cisbp__M2351	3.63	0.0907		3	474
transfac_pro__M06356	3.62	0.0907	ZNF425 (directAnnotation).	4	577
cisbp__M0540	3.62	0.0906		3	356
swissregulon__sacCer__LEU3	3.62	0.0905		3	253
transfac_pro__M00770	3.62	0.0905	CEBPA; CEBPB; CEBPD; CEBPE; CEBPG (directAnnotation).	3	341
predrem__nrMotif55	3.61	0.0904		6	2428
cisbp__M5593	3.61	0.0903	KLF16 (directAnnotation).	3	434
flyfactorsurvey__Sp1_SANGER_5_FBgn002 0378	3.61	0.0903	SP6; SP7; SP8; SP9 (inferredBy_Orthology).	3	274
tfdimers__MD00060	3.6	0.0903	CRX; ZNF333 (directAnnotation).	5	796
cisbp__M5523	3.58	0.0898		3	399
cisbp__M1257	3.58	0.0898		3	465
predrem__nrMotif1247	3.58	0.0898		4	791
taipale_tf_pairs__PITX1_HES7_NCRCGTGN NNGGATTA_CAP_repr	3.58	0.0897	HES7; PITX1 (directAnnotation).	3	399
hocomoco__HNF1B_HUMAN.H11MO.1.A	3.57	0.0896	HNF1B (directAnnotation).	3	465
cisbp__M4879	3.57	0.0895		3	299
taipale_cyt_meth__KLF13_NRCCACGCCCM YN_eDBD_meth	3.56	0.0894	KLF13 (directAnnotation).	5	617
taipale_cyt_meth__PAX3_NSGTCACGSNNA TTAN_eDBD	3.55	0.0892	PAX3 (directAnnotation).	3	434
cisbp__M5920	3.55	0.0892	TFAP2C (directAnnotation).	6	1612
elemento__CCTCCGC	3.53	0.0888		4	672
taipale__HNF1A_full_NRTTAATNATTAACN_ repr	3.53	0.0888	HNF1A (directAnnotation).	5	1441
transfac_pro__M08186	3.52	0.0886		3	364
neph__UW.Motif.0138	3.52	0.0885		3	356
elemento__TCGCGCA	3.51	0.0884		4	774
cisbp__M4521	3.51	0.0884	CTCF (directAnnotation).	3	399
transfac_pro__M06827	3.51	0.0883	ZBTB11 (directAnnotation).	8	2527
swissregulon__hs__EP300.p2	3.5	0.0882	EP300 (directAnnotation).	2	32
transfac_pro__M06711	3.49	0.0879	ZNF160 (directAnnotation).	3	305
predrem__nrMotif1506	3.48	0.0877		2	76
dbcorrdb__ZNF384__ENCSR000DYP_1__m [illegible]	3.48	0.0877	ZNF384 (directAnnotation).	3	341
jaspar__MA0467.1	3.47	0.0875	CRX (inferredBy_Orthology).	3	475

Supplementary Table S6 (continued)

transfac_pro__M09548	3.47	0.0875		3	360
taipale_cyt_meth__KLF15_RCCACGCCCMYN_eDBD_meth	3.47	0.0875	KLF15 (directAnnotation).	5	985
cisbp__M2176	3.46	0.0872		3	272
predrem__nrMotif1654	3.46	0.0872		3	283
neph__UW.Motif.0435	3.44	0.087		3	465
cisbp__M2269	3.44	0.087	CRX (inferredBy_Orthology).	3	500
stark__ATCWATG	3.44	0.087		2	77
transfac_pro__M06666	3.43	0.0868	ZNF442 (directAnnotation).	3	295
cisbp__M5521	3.43	0.0867	HNF1A (directAnnotation).	5	1264
taipale_cyt_meth__TFAP2B_NGCCNNNGGCN_eDBD_meth	3.4	0.0861	TFAP2B (directAnnotation).	3	361
predrem__nrMotif1157	3.4	0.086		2	15
cisbp__M5881	3.38	0.0856	TBX15 (directAnnotation).	4	614
stark__RTAAMA	3.38	0.0856	FOXC1; FOXC2; FOXS1 (inferredBy_Orthology).	3	577
taipale_cyt_meth__NRL_NWWWNTGCTGACN_eDBD_repr	3.37	0.0855	NRL (directAnnotation).	2	18
predrem__nrMotif2548	3.37	0.0855		4	621
predrem__nrMotif1403	3.37	0.0854		4	672
taipale_cyt_meth__ZNF821_NRGACRGACRGACRN_FL_meth_repr	3.37	0.0854	ZNF821 (directAnnotation).	3	356
jaspar__MA0942.1	3.36	0.0853		2	17
hocomoco__HNF1B_MOUSE.H11MO.1.A	3.36	0.0853	HNF1B (inferredBy_Orthology).	3	299
hocomoco__RARA_HUMAN.H11MO.2.A	3.36	0.0853	RARA (directAnnotation).	2	22
transfac_pro__M05431	3.36	0.0853	ZNF296 (directAnnotation).	3	577
transfac_pro__M06870	3.36	0.0853	ZNF131 (inferredBy_Orthology).	2	37
cisbp__M6444	3.36	0.0852	RARA (directAnnotation).	2	24
dbcorrdb__RELA__ENCSR000EBA_1__m3	3.36	0.0852	RELA (directAnnotation).	2	15
taipale_cyt_meth__NRL_NWWWNTGCTGACN_eDBD_meth	3.36	0.0852	NRL (directAnnotation).	2	24
taipale_cyt_meth__MAFA_NWWWNTGCTGACN_eDBD_meth	3.35	0.0851	MAFA (directAnnotation).	2	23
transfac_pro__M06910	3.35	0.0851	ZNF224 (directAnnotation).	3	402
cisbp__M5119	3.35	0.0851	OTX2 (inferredBy_Orthology).	3	364
taipale_cyt_meth__ZNF821_NRGACRGACRGACRN_FL	3.35	0.085	ZNF821 (directAnnotation).	4	672
predrem__nrMotif273	3.33	0.0847		2	32
cisbp__M6045	3.33	0.0846	MAFB (inferredBy_Orthology).	2	29
cisbp__M5163	3.33	0.0846	PITX1; PITX2; PITX3 (inferredBy_Orthology).	4	577
dbcorrdb__RAD21__ENCSR000EHX_1__m1	3.32	0.0845	RAD21 (directAnnotation).	4	475
elemento__CCCGGAG	3.32	0.0845		6	1977
cisbp__M4699	3.31	0.0843	HNF4A (directAnnotation).	2	27
taipale_tf_pairs__HOXB2_ETV7_TAATKNNNNGNNNNNNNCTTCCNN_CAP_repr	3.31	0.0842	ETV7; HOXB2 (directAnnotation).	2	28
taipale_cyt_meth__NRL_NWWWNTGCTGACN_FL_meth	3.3	0.0841	NRL (directAnnotation).	2	38
taipale__Mafb_DBD_NNNNTGCTGACN_repr	3.3	0.084	MAFB (inferredBy_Orthology).	2	37
transfac_pro__M06510	3.29	0.0838	ZFP62 (directAnnotation).	2	31
transfac_pro__M06808	3.29	0.0838	ZNF131 (directAnnotation).	2	48

Supplementary Table S6 (continued)

predrem__nrMotif2235	3.28	0.0837		2	35
stark__CMGGAAR	3.28	0.0837	ELF1; ELF2; ELF4 (inferredBy_Orthology).	3	307
transfac_pro__M05193	3.28	0.0836		2	50
taipale_cyt_meth__NRL_NWWWNTGCTGA CN_FL	3.28	0.0836	NRL (directAnnotation).	3	577
transfac_pro__M05700	3.28	0.0835	ZNF19 (directAnnotation).	4	695
predrem__nrMotif1508	3.28	0.0835		2	42
jaspar__MA1012.1	3.27	0.0834		3	465
hdpi__CNOT6	3.27	0.0834	CNOT6 (directAnnotation).	3	399
transfac_pro__M09245	3.27	0.0834		2	36
elemento__CCCTCCTC	3.27	0.0833		2	39
transfac_pro__M06560	3.26	0.0833	ZNF502 (directAnnotation).	2	48
predrem__nrMotif1264	3.26	0.0832		2	31
predrem__nrMotif1325	3.26	0.0831		4	682
elemento__CAGGCCC	3.25	0.0831		4	577
neph__UW.Motif.0272	3.25	0.0831		3	434
transfac_pro__M05359	3.25	0.083		2	56
dbcorrdb__KDM5A__ENCSR000AQL_1__m [illegible]	3.25	0.083	KDM5A (directAnnotation).	2	40
neph__UW.Motif.0293	3.25	0.083		4	434
transfac_pro__M02773	3.25	0.0829	KLF7 (inferredBy_Orthology).	5	1794
flyfactorsurvey__Bcd_Cell_FBgn0000166	3.25	0.0829		4	774
cisbp__M1185	3.23	0.0827		3	356
transfac_pro__M04784	3.23	0.0826	MYC (directAnnotation).	2	45
hocomoco__DDIT3_HUMAN.H11MO.0.D	3.23	0.0825	DDIT3 (directAnnotation).	2	35
dbcorrdb__POLR2A__ENCSR000DLJ_1__m [illegible]	3.22	0.0825	POLR2A [illegible]	2	67
transfac_pro__M07760	3.22	0.0824	TGIF1; TGIF2; TGIF2LX; TGIF2LY (inferredBy_Orthology).	5	1331
tfdimers__MD00281	3.22	0.0823	CDX2; HNF4A; HNF4G; NR2F1; NR2F2 (directAnnotation).	2	56
dbcorrdb__JUN__ENCSR000EEK_1__m1	3.22	0.0823	JUN (directAnnotation).	2	120
transfac_pro__M02802	3.22	0.0823	SOX1 (inferredBy_Orthology).	3	399
hocomoco__CRX_HUMAN.H11MO.0.B	3.21	0.0821	CRX (directAnnotation).	2	56
cisbp__M5915	3.21	0.0821	TFAP2A (directAnnotation).	7	2903
jaspar__MA0159.1	3.2	0.082	RXRA (directAnnotation).	2	73
homer__AAYTAGGTCA_RORgt	3.2	0.082		2	120
hocomoco__ZN524_HUMAN.H11MO.0.D	3.2	0.082	ZNF524 (directAnnotation).	2	69
transfac_pro__M08894	3.2	0.082	OVOL1; OVOL2 (directAnnotation).	2	66
neph__UW.Motif.0040	3.2	0.0819		3	338
predrem__nrMotif1021	3.2	0.0819		2	75
transfac_pro__M09243	3.2	0.0819		2	59
transfac_pro__M01429	3.19	0.0818	HOMEZ (directAnnotation).	4	954
taipale_tf_pairs__GCM2_PITX1_RTRCGGG NNGATTA_CAP_repr	3.19	0.0818	GCM2; PITX1 (directAnnotation).	2	79
tfdimers__MD00397	3.19	0.0818		2	72
tfdimers__MD00153	3.19	0.0818	CRX; DBP (directAnnotation).	2	54
taipale_cyt_meth__MAFG_NWWWNTGCTG ACN_eDBD_meth	3.19	0.0817	MAFG (directAnnotation).	2	48

Supplementary Table S6 (continued)

transfac_pro__M07690	3.18	0.0816		2	77
taipale_cyt_meth__MAFG_NWWWNTGCTGACN_eDBD	3.18	0.0816	MAFG (directAnnotation).	3	371
transfac_pro__M05561	3.18	0.0816	HIC2 (inferredBy_Orthology).	3	577
flyfactorsurvey__CG7745_SANGER_5_FBgn0033616	3.18	0.0815		3	338
homer__GSCTGTCACTCA_PBX1	3.16	0.0812	PBX1 (directAnnotation).	5	1052
swissregulon__hs__MAFB.p2	3.16	0.0812	MAFB (directAnnotation).	2	120
taipale_cyt_meth__ONECUT2_NTATTGATTWN_FL_meth	3.16	0.0812	ONECUT2 (directAnnotation).	2	83
dbcorrdb__SETDB1__ENCSR000EWD_1__m8	3.16	0.0812	SETDB1 (directAnnotation).	2	49
swissregulon__sacCer__USV1	3.16	0.0812		4	601
flyfactorsurvey__HLH106_SANGER_5_3_FBgn0015234	3.16	0.0812	SREBF1; SREBF2 (inferredBy_Orthology).	2	65
neph__UW.Motif.0561	3.16	0.0812		3	399
tiffin__TIFDMEM0000055	3.15	0.081		7	3043
taipale_cyt_meth__ETV7_NMGGAARNNYTTCCKN_FL_meth	3.15	0.081	ETV7 (directAnnotation).	2	82
taipale_tf_pairs__CUX1_PITX1_GGATTANNNNATCRATN_CAP_repr	3.15	0.081	CUX1; PITX1 (directAnnotation).	2	80
cisbp__M1443	3.15	0.081	HNF4A (inferredBy_Orthology).	2	122
tfdimers__MD00071	3.15	0.081	CEBPA; CEBPB; CEBPD; CEBPE; CEBPG; SOX10 (directAnnotation).	2	90
taipale_tf_pairs__HOXB2_ESRRB_TAATKRNNNNNNAAGGTCA_CAP_repr	3.15	0.081	ESRRB; HOXB2 (directAnnotation).	2	80
cisbp__M5609	3.15	0.0809	MAFK (directAnnotation).	2	74
transfac_pro__M09173	3.15	0.0808		2	82
tiffin__TIFDMEM0000077	3.14	0.0807		2	83
taipale_cyt_meth__ONECUT2_NTATTGATYN_eDBD_meth	3.14	0.0807	ONECUT2 (directAnnotation).	2	77
transfac_pro__M02776	3.14	0.0807	MAFK (inferredBy_Orthology).	2	83
homer__NATGTTGCAA_CEBP_AP1	3.14	0.0807	CEBPB; JUN (inferredBy_Orthology).	2	71
hocomoco__HNF6_HUMAN.H11MO.0.B	3.14	0.0807	ONECUT1 (directAnnotation).	2	84
taipale_tf_pairs__MEIS1_SOX2_TGACAKNNNAACAATGN_CAP_repr	3.14	0.0807	MEIS1; SOX2 (directAnnotation).	3	356
transfac_pro__M04909	3.13	0.0805	ZNF263 (directAnnotation).	2	65
transfac_pro__M06445	3.12	0.0804	ZNF283 (directAnnotation).	2	83
transfac_pro__M03181	3.12	0.0804		2	87
taipale_cyt_meth__ONECUT2_NTATCGATTTN_FL	3.12	0.0804	ONECUT2 (directAnnotation).	2	78
transfac_pro__M06476	3.12	0.0803	ZNF34 (directAnnotation).	4	928
cisbp__M5611	3.11	0.0802	MAFK (directAnnotation).	2	57
taipale__MAFK_DBD_NNNNNTGCTGAN	3.11	0.0801	MAFK (directAnnotation).	2	87
taipale_tf_pairs__ETV2_RFX5_NNNTTCCGSNNNNGCAACNN_CAP_repr	3.11	0.0801	ETV2; RFX5 (directAnnotation).	3	469
taipale__ONECUT1_full_NNAAAATCRATAWN	3.11	0.0801	ONECUT1 (directAnnotation).	2	99
factorbook__ZNF263	3.11	0.0801	ZNF263 (directAnnotation).	4	860
transfac_pro__M06722	3.11	0.0801	PRDM5 (directAnnotation).	2	120
taipale_cyt_meth__ETV7_NMGGAARNNYTTCCKN_FL	3.1	0.08	ETV7 (directAnnotation).	4	928

Supplementary Table S6 (continued)

transfac_pro__M06602	3.1	0.0799	ZNF669 (directAnnotation).	2	55
flyfactorsurvey__Oc_Cell_FBgn0004102	3.1	0.0799	OTX2 (inferredBy_Orthology).	5	1794
transfac_pro__M02878	3.09	0.0797	LEF1 (inferredBy_Orthology).	3	371
cisbp__M3695	3.09	0.0796	EP300 (directAnnotation).	2	79
cisbp__M6356	3.09	0.0796	MZF1 (directAnnotation).	2	94
factorbook__CREB	3.08	0.0795	ATF3 (directAnnotation).	2	80
transfac_pro__M09257	3.08	0.0795		2	97
transfac_pro__M06501	3.08	0.0795	ZNF76 (inferredBy_Orthology).	2	95
transfac_public__M00465	3.08	0.0794	POU6F1 (directAnnotation).	2	59
homer__ATTGCATCAT_Chop	3.07	0.0794	DDIT3 (inferredBy_Orthology).	2	82
stark__AATKACA	3.07	0.0793		2	73
cisbp__M0127	3.07	0.0793		2	94
predrem__nrMotif511	3.07	0.0792		2	73
taipale_cyt_meth__SP9_NCCACGCCCMYN_eDBD_meth	3.07	0.0792	SP9 (directAnnotation).	5	652
dbcorrdb__POLR2A__ENCSR000BGD_1__m1	3.07	0.0792	POLR2A (directAnnotation).	2	65
taipale__RARA_full_RGGTCANNNARRGGTCA	3.06	0.0791	RARA (directAnnotation).	2	121
cisbp__M5695	3.06	0.0791	ONECUT1 (directAnnotation).	2	120
taipale_tf_pairs__ETV2_EOMES_RCCGGANNNNNNNNNNNACACCTN_CAP_repr	3.06	0.079	EOMES; ETV2 (directAnnotation).	4	499
dbcorrdb__POLR2A__ENCSR000BIK_1__m[illegible]	3.06	0.079	POLR2A [illegible]	2	88
predrem__nrMotif1701	3.05	0.079		5	619
cisbp__M5769	3.05	0.0789	RARG (directAnnotation).	2	101
transfac_pro__M01332	3.05	0.0789	BARHL1 (directAnnotation).	3	364
transfac_pro__M08964	3.05	0.0789	RXRA (directAnnotation).	3	577
dbcorrdb__RAD21__ENCSR000EFJ_1__m1	3.05	0.0789	RAD21 (directAnnotation).	4	682
tiffin__TIFDMEM0000075	3.05	0.0788		2	120
transfac_pro__M03170	3.05	0.0788		2	63
predrem__nrMotif1571	3.04	0.0788		2	121
predrem__nrMotif503	3.04	0.0787		2	120
taipale_cyt_meth__ONECUT1_NTATTGATYN_eDBD_meth_repr	3.04	0.0786	ONECUT1 (directAnnotation).	2	94
homer__MTGATGCAAT_Atf4	3.04	0.0786	ATF4 (inferredBy_Orthology).	2	87
cisbp__M3315	3.03	0.0786	GATA1 (directAnnotation).	2	120
predrem__nrMotif2476	3.03	0.0786		2	120
predrem__nrMotif2148	3.03	0.0785		2	72
cisbp__M5763	3.03	0.0784	RARA (directAnnotation).	2	131
dbcorrdb__TRIM28__ENCSR000EVY_1__m[illegible]	3.03	0.0784	TRIM28 (directAnnotation).	2	88
flyfactorsurvey__acj6_SOLEXA_5_FBgn0000028	3.03	0.0784	POU4F1 (inferredBy_Orthology).	2	70
taipale__RARG_full_RRGGTCANNNARAGGTCA	3.03	0.0784	RARG (directAnnotation).	2	120
transfac_pro__M03177	3.03	0.0784		2	120
transfac_pro__M07315	3.02	0.0782	CEBPB (directAnnotation).	4	954
swissregulon__sacCer__UME6	3.02	0.0782		6	2276
elemento__CTCCTCCC	3.02	0.0782		2	82
transfac_pro__M01517	3.02	0.0782		4	954

Supplementary Table S6 (continued)

predrem__nrMotif1217	3.01	0.0781		2	133
taipale_cyt_meth__ONECUT3_NTATTGATYN_eDBD_meth	3.01	0.0781	ONECUT3 (directAnnotation).	2	97
cisbp__M5743	3.01	0.0781	POU4F3 (directAnnotation).	2	124
elemento__AGGAGGA	3.01	0.0781		2	97
transfac_pro__M09264	3.01	0.078		2	90
cisbp__M2042	3.01	0.078	OTX2 (inferredBy_Orthology).	2	74
hocomoco__PO4F1_HUMAN.H11MO.0.D	3.01	0.078	POU4F1 (directAnnotation).	4	849

8 cell

hocomoco__ZFX_HUMAN.H11MO.1.A	6.01	0.048	ZFX (directAnnotation).	158	3456
dbcorrdb__POLR2AphosphoS2__ENCSR000DYF_1__m12	5.62	0.046	POLR2A (directAnnotation).	99	1668
elemento__ATCCCAGCA	5.61	0.046		148	3343
hocomoco__ZN770_HUMAN.H11MO.0.C	5.14	0.0437	ZNF770 (directAnnotation).	121	2311
dbcorrdb__SREBF2__ENCSR000DYT_1__m[illegible]	5.04	0.0432	SREBF2 (directAnnotation).	179	4182
elemento__CCAGCCTGG	4.62	0.0411		169	3550
taipale_tf_pairs__E2F1_EOMES_RGGTGTNNNGGCGSNNTNNCRSNN_CAP	4.6	0.041	E2F1; EOMES (directAnnotation).	29	308
neph__UW.Motif.0022	4.59	0.041		176	3619
transfac_pro__M01588	4.57	0.0409	KLF4 (directAnnotation).	180	4532
dbcorrdb__ZNF384__ENCSR000EFP_1__m[illegible]	4.56	0.0408	ZNF384 (directAnnotation).	172	3761
transfac_pro__M00482	4.53	0.0407	PITX2 (directAnnotation).	145	3138
neph__UW.Motif.0600	4.53	0.0406		39	526
hocomoco__ZN770_HUMAN.H11MO.1.C	4.5	0.0405	ZNF770 (directAnnotation).	130	2606
hdpi__ZNF503	4.47	0.0404	ZNF503 (directAnnotation).	141	3227
hdpi__MTHFD1	4.45	0.0403	MTHFD1 [illegible]	143	2862
c2h2_zfs__M0448	4.41	0.0401		110	1755
transfac_pro__M03878	4.4	0.04	HIVEP2 (directAnnotation).	181	4438
hdpi__NXPH3	4.36	0.0398	NXPH3 (directAnnotation).	105	2182
dbcorrdb__ZNF384__ENCSR000EFP_1__m[illegible]	4.36	0.0398	ZNF384 (directAnnotation).	209	4905
hdpi__C9orf156	4.33	0.0396	TRMO (directAnnotation).	70	1075
hocomoco__TBX3_HUMAN.H11MO.0.C	4.32	0.0396	TBX3 (directAnnotation).	60	950
elemento__AATCCCAGC	4.3	0.0395		142	2744
hocomoco__SMAD3_HUMAN.H11MO.0.B	4.25	0.0393	SMAD3 (directAnnotation).	100	2112
transfac_pro__M03576	4.2	0.039	UBP1 (inferredBy_Orthology).	191	4571
transfac_public__M00008	4.14	0.0387	SP1 (directAnnotation).	167	4048
cisbp__M3919	4.14	0.0387	SP1 (directAnnotation).	26	275
transfac_pro__M01169	4.14	0.0387	IKZF1 (directAnnotation).	170	3712
hocomoco__KLF5_MOUSE.H11MO.0.A	4.12	0.0386	KLF5 (inferredBy_Orthology).	34	445
swissregulon__sacCer__ORC1	4.11	0.0386		128	2638
predrem__nrMotif992	4.09	0.0385		39	548
transfac_pro__M05845	4.05	0.0383	ZSCAN29 (directAnnotation).	110	2367
cisbp__M0365	4	0.038		204	4967
predrem__nrMotif1084	4	0.038		86	1643
predrem__nrMotif1872	3.98	0.038		158	3716
neph__UW.Motif.0090	3.98	0.0379		160	3728
cisbp__M6401	3.93	0.0377	OTX2 (directAnnotation).	157	3846
hocomoco__PAX5_HUMAN.H11MO.0.A	3.93	0.0377	PAX5 (directAnnotation).	207	4944

Supplementary Table S6 (continued)

neph__UW.Motif.0021	3.93	0.0377		188	4180
transfac_pro__M07266	3.92	0.0376	APEX1; EP300 (directAnnotation).	84	1788
neph__UW.Motif.0390	3.9	0.0375		106	2200
hdpi__SMAP1L	3.9	0.0375	SMAP2 (directAnnotation).	183	4364
swissregulon__hs__PITX1..3.p2	3.9	0.0375	PITX1; PITX2; PITX3 (directAnnotation).	177	4059
neph__UW.Motif.0015	3.89	0.0375		192	4533
transfac_pro__M07460	3.82	0.0372	KLF3 (directAnnotation).	43	623
elemento__CTGGGATTA	3.79	0.037		173	3690
neph__UW.Motif.0349	3.78	0.037		84	1490
dbcorrdb__IRF3__ENCSR000DZX_1__m4	3.78	0.0369	IRF3 (directAnnotation).	131	2958
scertf__pachkov.ORC1	3.76	0.0369		121	2542
yetfasco__YML065W_1549	3.75	0.0368		159	3671
transfac_pro__M06210	3.72	0.0366	ZNF33A (directAnnotation).	36	586
dbcorrdb__POLR3A__ENCSR000DNU_1__m1	3.72	0.0366	POLR3A (directAnnotation).	217	4977
predrem__nrMotif233	3.71	0.0366		77	1473
hdpi__TRIM21	3.71	0.0366	TRIM21 (directAnnotation).	152	3451
transfac_pro__M04904	3.71	0.0366	POLR3A [illegible]	92	1766
neph__UW.Motif.0066	3.7	0.0366		34	495
taipale_cyt_meth__KLF2_NRCCACRCCCN_eDBD	3.68	0.0364	KLF2 (directAnnotation).	31	434
dbcorrdb__RAD21__ENCSR000EHX_1__m6	3.67	0.0364	RAD21 (directAnnotation).	101	2107
dbcorrdb__ZNF384__ENCSR000DYP_1__m	3.67	0.0364	ZNF384 (directAnnotation).	87	1385
dbcorrdb__SMARCA4__ENCSR000EZC_1__m9	3.66	0.0364	SMARCA4 (directAnnotation).	21	196
transfac_pro__M04632	3.65	0.0363	SREBF1 (directAnnotation). NR0B1 (inferredBy_Orthology).	54	992
elemento__AGGAGCG	3.65	0.0363		32	412
dbcorrdb__BDP1__ENCSR000DOK_1__m2	3.65	0.0363	BDP1 (directAnnotation).	178	4331
hocomoco__KLF4_HUMAN.H11MO.0.A	3.64	0.0363	KLF4 (directAnnotation).	150	3441
predrem__nrMotif29	3.63	0.0362		47	823
neph__UW.Motif.0176	3.6	0.036		140	3345
elemento__TCCCAGCAC	3.59	0.036		174	4101
yetfasco__YML113W_1416	3.57	0.0359		60	925
neph__UW.Motif.0460	3.57	0.0359		191	4999
hdpi__AKR1A1	3.57	0.0359	AKR1A1 (directAnnotation).	99	2012
cisbp__M1377	3.56	0.0359		30	434
yetfasco__YPL128C_2178	3.56	0.0359		29	427
hocomoco__KLF4_MOUSE.H11MO.0.A	3.56	0.0358	KLF4 (inferredBy_Orthology).	51	845
neph__UW.Motif.0173	3.54	0.0358		29	403
transfac_pro__M01721	3.52	0.0357	PURA (directAnnotation).	95	2016
transfac_pro__M03553	3.5	0.0356	KLF3 (directAnnotation).	47	770
tfdimers__MD00390	3.5	0.0356	CRX; NFIA; NFIC; RAX (directAnnotation).	80	1658
cisbp__M6421	3.48	0.0355	PLAG1 (directAnnotation).	24	271
fantom__motif148_NTCSYSTYT	3.48	0.0355		58	1073
dbcorrdb__POLR3A__ENCSR000DNU_1__m2	3.48	0.0355	POLR3A (directAnnotation).	126	2376
elemento__CCCAGCC	3.47	0.0354		172	4062
neph__UW.Motif.0568	3.47	0.0354		49	780

Supplementary Table S6 (continued)

transfac_pro__M00646	3.46	0.0354	HNF1B (directAnnotation).	151	3785
transfac_pro__M03814	3.46	0.0354	KLF5 (directAnnotation).	41	645
dbcorrdb__BRF1__ENCSR000DNW_1__m1	3.42	0.0352	BRF1 (directAnnotation).	195	4829
hdpi__H2AFY	3.4	0.0351	H2AFY (directAnnotation).	144	3076
predrem__nrMotif1918	3.38	0.035		30	443
cisbp__M1871	3.38	0.035	KLF4 (inferredBy_Orthology).	90	1815
transfac_pro__M02114	3.38	0.035	PITX2 (directAnnotation).	198	4941
dbcorrdb__NR3C1__ENCSR000BHE_1__m5	3.38	0.035	NR3C1 (directAnnotation).	43	737
cisbp__M5211	3.37	0.035	ZNF362; ZNF384 (inferredBy_Orthology).	170	4163
swissregulon__hs__KLF4.p3	3.37	0.0349	KLF4 (directAnnotation).	87	1707
hocomoco__PLAG1_HUMAN.H11MO.0.D	3.36	0.0349	PLAG1 (directAnnotation).	152	3856
dbcorrdb__MAFK__ENCSR000DYV_1__m3	3.36	0.0349	MAFK (directAnnotation).	171	4205
transfac_pro__M00706	3.35	0.0349	GTF2I (directAnnotation).	29	390
cisbp__M0442	3.35	0.0348	ZBTB3 (inferredBy_Orthology).	150	3215
tfdimers__MD00520	3.35	0.0348	MYCN; MZF1 (directAnnotation).	39	562
transfac_pro__M07602	3.35	0.0348	TFCP2 (directAnnotation).	176	4174
tfdimers__MD00537	3.34	0.0348	EP300; PURA (directAnnotation).	47	787
yetfasco__YDR146C_569	3.33	0.0347	GTF3A; ZXDA; ZXDB; ZXDC (inferredBy_Orthology).	137	3377
transfac_pro__M01646	3.32	0.0347	ZNF112; ZNF155; ZNF214; ZNF221; ZNF222; ZNF223; ZNF224; ZNF226; ZNF227; ZNF230; ZNF233; ZNF234; ZNF235; ZNF239; ZNF284; ZNF285; ZNF664 (inferredBy_Orthology).	142	3096
hdpi__FHL2	3.32	0.0347	FHL2 (directAnnotation).	29	435
cisbp__M6127	3.32	0.0347	KLF4 (inferredBy_Orthology).	96	2079
neph__UW.Motif.0012	3.3	0.0346		117	2415
jaspar__MA0402.1	3.29	0.0345	GTF3A; ZXDA; ZXDB; ZXDC (inferredBy_Orthology).	121	2907
neph__UW.Motif.0669	3.29	0.0345		115	2118
jaspar__MA0285.1	3.28	0.0345	ZNF112; ZNF155; ZNF214; ZNF221; ZNF222; ZNF223; ZNF224; ZNF226; ZNF227; ZNF230; ZNF233; ZNF234; ZNF235; ZNF239; ZNF284; ZNF285; ZNF664 (inferredBy_Orthology).	185	4440
transfac_pro__M00323	3.28	0.0345		23	250
swissregulon__sacCer__YML081W	3.28	0.0345		37	636
fantom__motif63_GTNCCA	3.27	0.0344		33	484
hocomoco__RARG_HUMAN.H11MO.2.D	3.27	0.0344	RARG (directAnnotation).	70	1399
neph__UW.Motif.0142	3.27	0.0344		21	221
hdpi__NAP1L1	3.26	0.0344	NAP1L1 (directAnnotation).	183	4402
hdpi__SOCS4	3.26	0.0344	SOCS4 (directAnnotation).	35	562
transfac_pro__M04595	3.26	0.0344	SALL2 (directAnnotation).	93	2115
transfac_pro__M04859	3.26	0.0344	SMC3 (directAnnotation).	168	3985

Supplementary Table S6 (continued)

predrem__nrMotif12	3.25	0.0343		49	821
dbcorrdb__POLR3A__ENCSR000DNU_1__m3	3.25	0.0343	POLR3A (directAnnotation).	194	4498
elemento__CCCACCC	3.25	0.0343		19	206
hdpi__SEMA4A	3.24	0.0343	SEMA4A [illegible]	149	3162
dbcorrdb__EZH2__ENCSR000ARH_1__m3	3.24	0.0343	EZH2 (directAnnotation).	38	606
neph__UW.Motif.0379	3.23	0.0343		141	2866
transfac_pro__M01168	3.22	0.0342	SREBF1; SREBF2 (directAnnotation).	164	4260
cisbp__M0543	3.22	0.0342		22	293
taipale_tf_pairs__GCM2_HES7_RTRNKGGTNNNGCACGYGNN_CAP_repr	3.22	0.0342	GCM2; HES7 (directAnnotation).	34	502
flyfactorsurvey__sqz_SANGER_5_FBgn0010768	3.22	0.0342	ZNF362; ZNF384 (inferredBy_Orthology).	136	3324
cisbp__M2090	3.21	0.0341	ZNF112; ZNF155; ZNF214; ZNF221; ZNF222; ZNF223; ZNF224; ZNF226; ZNF227; ZNF230; ZNF233; ZNF234; ZNF235; ZNF239; ZNF284; ZNF285; ZNF664 (inferredBy_Orthology).	186	4472
transfac_pro__M06352	3.21	0.0341	ZNF425 (directAnnotation).	194	4928
transfac_pro__M08829	3.21	0.0341	SMAD5 (directAnnotation).	204	4924
transfac_pro__M03852	3.2	0.0341	SREBF2 (directAnnotation).	99	2109
cisbp__M6324	3.2	0.0341	KLF4 (directAnnotation).	119	2672
transfac_public__M00506	3.19	0.0341		31	461
transfac_pro__M09021	3.19	0.034		64	1131
neph__UW.Motif.0496	3.19	0.034		116	2627
elemento__GCCTCCC	3.19	0.034		139	2934
hocomoco__SMAD4_MOUSE.H11MO.0.A	3.19	0.034	SMAD4 (inferredBy_Orthology).	98	2199
cisbp__M5012	3.19	0.034	TCF12; TCF3; TCF4 (inferredBy_Orthology).	46	796
tfdimers__MD00162	3.18	0.034	BACH1; BACH2; MAF; MAFB; MAFF; MAFG; MAFK; NFE2; NFE2L1; NFE2L2; NFE2L3; PURA (directAnnotation).	28	416
elemento__CCTGCCTC	3.18	0.034		185	4460
transfac_pro__M07056	3.17	0.0339	PITX2 (directAnnotation).	95	1599
neph__UW.Motif.0487	3.15	0.0338		38	612
dbcorrdb__CEBPZ__ENCSR000EDO_1__m[illegible]	3.14	0.0338	CEBPZ (directAnnotation).	106	2260
transfac_pro__M07261	3.14	0.0338	KLF2 (directAnnotation).	38	616
hocomoco__ZN335_HUMAN.H11MO.1.A	3.14	0.0338	ZNF335 (directAnnotation).	41	696
dbcorrdb__RELA__ENCSR000EBM_1__m2	3.13	0.0338	RELA (directAnnotation).	44	784
tfdimers__MD00443	3.13	0.0338	SREBF2; WT1 (directAnnotation).	45	791
transfac_pro__M01973	3.13	0.0337	PLAG1 (directAnnotation).	22	285
transfac_pro__M03868	3.12	0.0337	TFCP2 (directAnnotation).	115	2735
neph__UW.Motif.0067	3.11	0.0336		41	665
cisbp__M2376	3.1	0.0336		114	2504
cisbp__M0484	3.07	0.0335		172	4185
tfdimers__MD00366	3.07	0.0335	MTF1; OVOL2 (directAnnotation).	41	690
taipale_cyt_meth__KLF15_NCCACGCCCMYN_eDBD	3.07	0.0334	KLF15 (directAnnotation).	25	327

Supplementary Table S6 (continued)

hdpi__CBX7	3.07	0.0334	CBX7 (directAnnotation).	33	540
hdpi__ZNF304	3.06	0.0334	ZNF304 (directAnnotation).	171	3968
hdpi__AFF4	3.06	0.0334	AFF4 (directAnnotation).	83	1742
taipale_tf_pairs__GCM1_NHLH1_NCAGCTG NNNNNNNNTRCGGG_CAP_repr	3.05	0.0334	GCM1; NHLH1 (directAnnotation).	23	284
taipale_cyt_meth__PITX2_NTAATCCN_eDB_	3.05	0.0334	PITX2 (directAnnotation).	77	1588
transfac_pro__M01859	3.05	0.0333	TFAP2C (directAnnotation).	188	4772
hocomoco__OLIG2_HUMAN.H11MO.0.B	3.04	0.0333	OLIG2 (directAnnotation).	24	307
cisbp__M1396	3.04	0.0333		23	306
hdpi__RAB7A	3.04	0.0333	RAB7A (directAnnotation).	171	4028
elemento__CCCCAGC	3.03	0.0333		49	852
dbcorrdb__CHD1__ENCSR000AQK_1__m1	3.03	0.0332	CHD1 (directAnnotation).	48	862
dbcorrdb__NFYB__ENCSR000DNR_1__m2	3.03	0.0332	NFYB (directAnnotation).	191	4658
yetfasco__YNL027W_516	3.02	0.0332	ZNF112; ZNF155; ZNF214; ZNF221; ZNF222; ZNF223; ZNF224; ZNF226; ZNF227; ZNF230; ZNF233; ZNF234; ZNF235; ZNF239; ZNF284; ZNF285; ZNF664 (inferredBy_Orthology).	146	3174
scertf__spivak.CRZ1	3	0.0331	ZNF112; ZNF155; ZNF214; ZNF221; ZNF222; ZNF223; ZNF224; ZNF226; ZNF227; ZNF230; ZNF233; ZNF234; ZNF235; ZNF239; ZNF284; ZNF285; ZNF664 (inferredBy_Orthology).	211	4758
transfac_pro__M01865	3	0.0331	KLF13 (directAnnotation).	29	446

ICM

hocomoco__ZN770_HUMAN.H11MO.0.C	5	0.0328	ZNF770 (directAnnotation).	807	4996
hdpi__TAGLN2	4.98	0.0328	TAGLN2 (directAnnotation).	792	4979
dbcorrdb__POLR2AphosphoS2__ENCSR000 DYF_1__m12	4.64	0.0317	POLR2A (directAnnotation).	750	4829
elemento__CTGGGATTA	4.61	0.0316		457	2656
c2h2_zfs__M0448	4.55	0.0314		677	3895
transfac_public__M00255	4.54	0.0314		220	1148
transfac_pro__M00482	4.49	0.0312	PITX2 (directAnnotation).	470	2697
swissregulon__hs__PITX1..3.p2	4.43	0.031	PITX1; PITX2; PITX3 (directAnnotation).	618	3780
hdpi__MTHFD1	4.4	0.0309	MTHFD1 [illegible]	830	4913
swissregulon__sacCer__ORC1	4.34	0.0307		703	4624
hocomoco__ZFX_HUMAN.H11MO.1.A	4.32	0.0306	ZFX (directAnnotation).	705	4198
elemento__CCCAGCC	4.29	0.0305		562	3535
neph__UW.Motif.0022	4.28	0.0305		688	4086
hdpi__SMPX	4.24	0.0304	SMPX (directAnnotation).	513	3285
hocomoco__ZN121_HUMAN.H11MO.0.C	4.22	0.0303	ZNF121 (directAnnotation).	756	4991
scertf__pachkov.ORC1	4.21	0.0303		537	3328
cisbp__M0885	4.21	0.0303		646	3984
yetfasco__YML065W_1549	4.21	0.0303		546	3400
cisbp__M5785	4.19	0.0302	RHOXF1 [illegible]	489	2994
hdpi__NAP1L1	4.19	0.0302	NAP1L1 (directAnnotation).	734	4430
neph__UW.Motif.0669	4.15	0.0301		745	4820
hocomoco__PAX5_HUMAN.H11MO.0.A	4.15	0.0301	PAX5 (directAnnotation).	776	4981

Supplementary Table S6 (continued)

neph__UW.Motif.0390	4.13	0.03		425	2658
dbcorrdb__ZNF384__ENCSR000EFP_1__m	4.11	0.03	ZNF384 (directAnnotation).	759	4914
elemento__TGAGCCAC	4.09	0.0299		731	4998
dbcorrdb__POLR3A__ENCSR000DNU_1__m1	4.06	0.0298	POLR3A (directAnnotation).	801	4994
hdpi__NXPH3	4.05	0.0297	NXPH3 (directAnnotation).	722	4354
hdpi__C9orf156	4.01	0.0296	TRMO (directAnnotation).	792	4995
elemento__ATCCCAGCA	4.01	0.0296		397	2250
neph__UW.Motif.0092	4.01	0.0296		754	4859
transfac_pro__M08829	4	0.0296	SMAD5 (directAnnotation).	777	4748
cisbp__M0484	3.99	0.0296		740	4715
jaspar__MA0118.1	3.97	0.0295		263	1567
elemento__AATCCCAGC	3.97	0.0295		588	3581
yetfasco__YDR043C_2148	3.96	0.0295		219	1187
taipale__RHOXF1_full_NTRAKCCN	3.95	0.0294	RHOXF1	509	3124
jaspar__MA0583.1	3.95	0.0294		785	4990
cisbp__M3926	3.94	0.0294	SP1 (directAnnotation).	628	4111
dbcorrdb__ZNF384__ENCSR000EFP_1__m	3.93	0.0294	ZNF384 (directAnnotation).	676	3976
cisbp__M2376	3.93	0.0294		769	4915
transfac_pro__M04983	3.93	0.0293		559	3631
transfac_pro__M01798	3.93	0.0293	POLR3A	761	4787
transfac_pro__M02114	3.92	0.0293	PITX2 (directAnnotation).	734	4947
hdpi__TRIM21	3.91	0.0293	TRIM21 (directAnnotation).	769	4755
cisbp__M0505	3.91	0.0293		530	3528
hdpi__PIR	3.9	0.0293	PIR (directAnnotation).	787	4999
transfac_public__M00196	3.9	0.0292	SP1 (directAnnotation).	630	4138
hdpi__SCC-112	3.89	0.0292	PDS5A (directAnnotation).	588	3817
jaspar__MA0103.2	3.89	0.0292	ZEB1 (directAnnotation).	86	373
transfac_pro__M03814	3.88	0.0292	KLF5 (directAnnotation).	133	651
cisbp__M1434	3.88	0.0292	NR2F6 (directAnnotation).	182	979
cisbp__M1939	3.88	0.0292		193	1083
transfac_pro__M00646	3.86	0.0291	HNF1B (directAnnotation).	598	3766
hdpi__RAB7A	3.85	0.0291	RAB7A (directAnnotation).	776	4977
elemento__CCCCAGC	3.85	0.0291		663	4552
hdpi__SMAP1L	3.85	0.0291	SMAP2 (directAnnotation).	800	4969
dbcorrdb__POLR3A__ENCSR000DNU_1__m3	3.84	0.0291	POLR3A (directAnnotation).	732	4767
taipale_cyt_meth__ZNF787_TGCCTCMGTTTMCCY_FL	3.83	0.029	ZNF787 (directAnnotation).	707	4935
dbcorrdb__SREBF2__ENCSR000DYT_1__m	3.82	0.029	SREBF2 (directAnnotation).	741	4926
transfac_pro__M00932	3.82	0.029	SP1; SP2; SP3; SP4 (directAnnotation).	327	1874
cisbp__M1926	3.8	0.0289	ZEB1 (directAnnotation).	128	629
neph__UW.Motif.0238	3.8	0.0289		635	4060
cisbp__M5783	3.8	0.0289	RHOXF1	440	2568
tfdimers__MD00467	3.79	0.0289	ARID3A; PBX1 (directAnnotation).	380	2222
flyfactorsurvey__CG5669_SANGER_10_FBgn0039169	3.79	0.0289	SP1; SP2; SP3; SP4 (inferredBy_Orthology).	558	3771
transfac_pro__M07261	3.78	0.0289	KLF2 (directAnnotation).	749	4949
factorbook__SP1	3.77	0.0288	SP1; SP2 (directAnnotation).	480	2844
neph__UW.Motif.0116	3.75	0.0288		665	4515

Supplementary Table S6 (continued)

transfac_pro__M01100	3.75	0.0288	ZBTB7A (directAnnotation).	409	2575
factorbook__UA12	3.74	0.0287		70	287
homer__GGCCCCGCCCCC_Sp1	3.73	0.0287		623	4061
elemento__CCAGCCTGG	3.73	0.0287		654	3869
transfac_pro__M03878	3.72	0.0287	HIVEP2 (directAnnotation).	725	4751
cisbp__M4870	3.71	0.0287	SP1; SP2; SP3; SP4 (inferredBy_Orthology).	441	2878
neph__UW.Motif.0015	3.68	0.0285		724	4706
dbcorrdb__ZNF384__ENCSR000DYP_1__m	3.66	0.0285	ZNF384 (directAnnotation).	556	3394
hocomoco__ZN770_HUMAN.H11MO.1.C	3.66	0.0285	ZNF770 (directAnnotation).	768	4940
taipale__RHOXF1_DBD_NTRAKCCN	3.64	0.0284	RHOXF1	464	2740
transfac_pro__M07615	3.63	0.0284	SP3 (directAnnotation).	650	4485
hocomoco__SP1_HUMAN.H11MO.1.A	3.62	0.0284	SP1 (directAnnotation).	711	4747
cisbp__M0365	3.62	0.0283		771	4798
cisbp__M1495	3.62	0.0283		210	1179
hdpi__AKR1A1	3.61	0.0283	AKR1A1 (directAnnotation).	770	4993
cisbp__M6219	3.61	0.0283	ESR2 (directAnnotation).	105	485
elemento__CCCCAGG	3.61	0.0283		240	1287
neph__UW.Motif.0327	3.59	0.0283		712	4752
dbcorrdb__ZEB1__ENCSR000BND_1__m1	3.59	0.0283	ZEB1 (directAnnotation).	257	1543
elemento__CTGCCCC	3.59	0.0283		149	792
transfac_pro__M07921	3.57	0.0282	ZNF358; ZNF768 (inferredBy_Orthology).	455	2738
transfac_pro__M06191	3.57	0.0282	ZXDB (directAnnotation).	109	494
cisbp__M5210	3.57	0.0282	SP6; SP7; SP8; SP9 (inferredBy_Orthology).	434	2643
dbcorrdb__POLR3A__ENCSR000DNU_1__m2	3.56	0.0282	POLR3A (directAnnotation).	747	4702
dbcorrdb__GTF3C2__ENCSR000DOD_1__m1	3.56	0.0281	GTF3C2 (directAnnotation).	744	4584
transfac_pro__M06190	3.55	0.0281	ZXDA (directAnnotation).	115	534
transfac_pro__M09005	3.54	0.0281	SP4	609	4148
hdpi__ZNF503	3.54	0.0281	ZNF503 (directAnnotation).	649	3783
transfac_pro__M03790	3.52	0.028	NR1H4 (directAnnotation).	149	754
cisbp__M0470	3.52	0.028		174	943
transfac_pro__M00720	3.51	0.028		777	4988
dbcorrdb__IRF3__ENCSR000DZX_1__m4	3.51	0.028	IRF3 (directAnnotation).	683	4482
dbcorrdb__ZNF384__ENCSR000EFP_1__m	3.51	0.028	ZNF384 (directAnnotation).	599	3740
cisbp__M4483	3.5	0.028	ZEB1 (directAnnotation).	129	625
flyfactorsurvey__Sp1_SOLEXA_2.5_FBgn0020378	3.5	0.028	SP6; SP7; SP8; SP9 (inferredBy_Orthology).	401	2408
hdpi__CBX7	3.49	0.0279	CBX7 (directAnnotation).	339	2020
transfac_pro__M01161	3.49	0.0279		322	1866
transfac_pro__M07280	3.49	0.0279	NR1H2; NR1H3; NR1I2; NR1I3; NR2F1; NR2F2; RARA; RARB; RARG; RXRA; RXRB (directAnnotation).	132	685
elemento__CCCTGCC	3.48	0.0279		552	3767
transfac_pro__M04854	3.47	0.0279	SMC3 (directAnnotation).	658	4468
c2h2_zfs__M3835	3.47	0.0279	ZBTB7A (directAnnotation).	325	1916
predrem__nrMotif120	3.47	0.0278		488	3283
c2h2_zfs__M3425	3.46	0.0278		227	1302

Supplementary Table S6 (continued)

neph__UW.Motif.0658	3.45	0.0278		497	3308
factorbook__UA13	3.45	0.0278		589	3939
scertf__spivak.CRZ1	3.45	0.0278	ZNF112; ZNF155; ZNF214; ZNF221; ZNF222; ZNF223; ZNF224; ZNF226; ZNF227; ZNF230; ZNF233; ZNF234; ZNF235; ZNF239; ZNF284; ZNF285; ZNF664 (inferredBy_Orthology).	727	4603
hdpi__MXD4	3.44	0.0278	MXD4 (directAnnotation).	166	872
transfac_pro__M07486	3.44	0.0277		189	976
factorbook__UA3	3.43	0.0277	ZBTB7A (directAnnotation).	305	1739
transfac_pro__M04859	3.43	0.0277	SMC3 (directAnnotation).	733	4848
transfac_pro__M01177	3.43	0.0277	SREBF2 (directAnnotation).	748	4981
swissregulon__hs__SP1.p2	3.42	0.0277	SP1 (directAnnotation).	568	3709
neph__UW.Motif.0021	3.42	0.0277		796	4986
neph__UW.Motif.0023	3.42	0.0277		543	3494
elemento__CCAGGCC	3.41	0.0277		477	2983
transfac_pro__M01169	3.41	0.0277	IKZF1 (directAnnotation).	753	4836
yetfasco__YMR182C_531	3.41	0.0277		167	918
cisbp__M6325	3.41	0.0277	KLF6 (directAnnotation).	238	1380
fantom__motif169_GCCTGGCC	3.4	0.0276		717	4803
hdpi__HLCS	3.4	0.0276	HLCS (directAnnotation).	730	4999
taipale_cyt_meth__ZNF787_TGCCTCMGTTTMCCY_FL_meth_repr	3.39	0.0276	ZNF787 (directAnnotation).	133	683
swissregulon__sacCer__RGM1	3.38	0.0276		171	942
hdpi__AFF4	3.38	0.0276	AFF4 (directAnnotation).	757	4898
transfac_pro__M04904	3.38	0.0275	POLR3A [illegible]	714	4874
dbcorrdb__IRF1__ENCSR000EGT_1__m1	3.37	0.0275	IRF1 (directAnnotation).	632	4029
transfac_pro__M01033	3.37	0.0275	HNF4A (directAnnotation).	456	2964
yetfasco__YLR131C_1332	3.37	0.0275	GTF3A; ZXDA; ZXDB; ZXDC (inferredBy_Orthology).	706	4788
tfdimers__MD00116	3.37	0.0275	CRX; SPZ1 (directAnnotation).	657	4354
c2h2_zfs__M0442	3.35	0.0275		738	4919
neph__UW.Motif.0090	3.35	0.0275		780	4993
neph__UW.Motif.0443	3.35	0.0275		573	3637
predrem__nrMotif55	3.34	0.0274		221	1182
hocomoco__ZFX_MOUSE.H11MO.1.B	3.34	0.0274	ZFY [illegible]	401	2372
dbcorrdb__SP1__ENCSR000BHK_1__m2	3.34	0.0274	SP1 (directAnnotation).	489	3098
transfac_public__M00008	3.34	0.0274	SP1 (directAnnotation).	651	4231
yetfasco__YML113W_1416	3.34	0.0274		641	4321
cisbp__M0394	3.34	0.0274	PLAGL1 (inferredBy_Orthology).	717	4986
hocomoco__SALL1_MOUSE.H11MO.0.D	3.33	0.0274		730	4536
hdpi__PIK3C3	3.33	0.0274	PIK3C3 (directAnnotation).	741	4991
transfac_pro__M01721	3.33	0.0274	PURA (directAnnotation).	723	4954
hocomoco__COT1_HUMAN.H11MO.1.C	3.33	0.0274	NR2F1 (directAnnotation).	209	1078
hocomoco__SMAD3_HUMAN.H11MO.0.B	3.33	0.0274	SMAD3 (directAnnotation).	631	4241
taipale_cyt_meth__PITX2_NTAATCCN_eDB_	3.32	0.0274	PITX2 (directAnnotation).	544	3655
dbcorrdb__CUX1__ENCSR000EFO_1__m5	3.32	0.0274	CUX1 (directAnnotation).	221	1264
hocomoco__PITX2_HUMAN.H11MO.0.D	3.31	0.0273	PITX2 (directAnnotation).	341	2053

Supplementary Table S6 (continued)

transfac_public__M00048	3.31	0.0273		345	2058
yetfasco__YOL067C_1494	3.31	0.0273		88	434
transfac_public__M00506	3.3	0.0273		438	2821
elemento__GGGACCC	3.29	0.0273		351	2245
hocomoco__RARA_MOUSE.H11MO.3.A	3.28	0.0272	RARA (inferredBy_Orthology).	233	1358
taipale_tf_pairs__E2F1_EOMES_RGGTGTN NNGGCGSNNTNNCRSNN_CAP	3.28	0.0272	E2F1; EOMES (directAnnotation).	709	4993
neph__UW.Motif.0012	3.28	0.0272		770	4835
transfac_pro__M03576	3.28	0.0272	UBP1 (inferredBy_Orthology).	661	4096
hdpi__DUS3L	3.28	0.0272	DUS3L (directAnnotation).	655	4467
elemento__CAGGCCC	3.27	0.0272		458	2989
predrem__nrMotif1063	3.27	0.0272		261	1473
cisbp__M6479	3.27	0.0272	SP1 (directAnnotation).	410	2725
cisbp__M1452	3.26	0.0272	RORA (inferredBy_Orthology).	179	1030
cisbp__M0956	3.26	0.0272	PITX3 (inferredBy_Orthology).	241	1329
hdpi__H2AFY	3.26	0.0272	H2AFY (directAnnotation).	483	3156
cisbp__M6127	3.24	0.0271	KLF4 (inferredBy_Orthology).	299	1857
hdpi__UGP2	3.24	0.0271	UGP2 (directAnnotation).	680	4459
transfac_pro__M07056	3.24	0.0271	PITX2 (directAnnotation).	644	4245
hocomoco__HTF4_HUMAN.H11MO.0.A	3.23	0.0271	TCF12 (directAnnotation).	123	648
cisbp__M6480	3.23	0.0271	SP1 (directAnnotation).	618	4274
dbcorrdb__JUND__ENCSR000DYS_1__m1	3.23	0.0271	JUND (directAnnotation).	122	648
transfac_pro__M01616	3.23	0.0271	GTF3A; ZXDA; ZXDB; ZXDC (inferredBy_Orthology).	725	4995
hocomoco__SP2_HUMAN.H11MO.1.B	3.23	0.0271	SP2 (directAnnotation).	561	3705
transfac_pro__M08955	3.23	0.0271	NR1H4 (directAnnotation).	561	3794
hdpi__FGF19	3.21	0.027	FGF19 (directAnnotation).	654	4178
dbcorrdb__CEBPZ__ENCSR000EDO_1__m [illegible]	3.21	0.027	CEBPZ (directAnnotation).	729	4912
elemento__CCTGCCC	3.21	0.027		531	3484
predrem__nrMotif1180	3.21	0.027		100	486
predrem__nrMotif2511	3.21	0.027		215	1194
transfac_pro__M00931	3.21	0.027	SP1; SP3; SP4 (directAnnotation).	672	4679
cisbp__M4871	3.21	0.027	SP1; SP2; SP3; SP4 (inferredBy_Orthology).	650	4320
transfac_pro__M07348	3.2	0.027	TFAP2A (directAnnotation).	110	548
jaspar__MA0267.1	3.19	0.0269	GTF3A; ZXDA; ZXDB; ZXDC (inferredBy_Orthology).	702	4826
elemento__GCCTCCC	3.19	0.0269		629	4177
transfac_pro__M07371	3.19	0.0269	ZEB1 (directAnnotation).	638	4547
cisbp__M2072	3.18	0.0269	GTF3A; ZXDA; ZXDB; ZXDC (inferredBy_Orthology).	704	4855
hocomoco__SP1_MOUSE.H11MO.1.A	3.18	0.0269	SP1 [illegible]	80	354
flyfactorsurvey__lola-PL_SANGER_2.5_FBgn0005630	3.18	0.0269		705	4860
hdpi__JARID1A	3.18	0.0269	KDM5A (directAnnotation).	627	4064
transfac_pro__M05775	3.18	0.0269	ZXDC (directAnnotation).	282	1696

Supplementary Table S6 (continued)

flyfactorsurvey__CG5669_SOLEXA_5_FBgn0039169	3.17	0.0269	SP1; SP2; SP3; SP4 (inferredBy_Orthology).	658	4422
taipale_cyt_meth__SP9_NCCACGCCCMYN_eDBD_meth	3.17	0.0269	SP9 (directAnnotation).	401	2639
hocomoco__SP3_HUMAN.H11MO.0.B	3.17	0.0269	SP3 (directAnnotation).	677	4856
transfac_pro__M01168	3.17	0.0269	SREBF1; SREBF2 (directAnnotation).	648	4345
transfac_pro__M01859	3.16	0.0268	TFAP2C (directAnnotation).	745	4772
dbcorrdb__RAD21__ENCSR000EHX_1__m6	3.16	0.0268	RAD21 (directAnnotation).	667	4390
transfac_pro__M07063	3.15	0.0268	SP1 (directAnnotation).	503	3303
transfac_pro__M07266	3.15	0.0268	APEX1; EP300 (directAnnotation).	701	4799
yetfasco__YBL103C_1446	3.14	0.0268		71	323
swissregulon__sacCer__SWI5	3.14	0.0268	GTF3A; ZXDA; ZXDB; ZXDC (inferredBy_Orthology).	744	4992
cisbp__M6482	3.14	0.0268	SP3 (directAnnotation).	560	3880
hdpi__UBB	3.14	0.0268	UBB (directAnnotation).	605	3924
transfac_pro__M07395	3.13	0.0268	SP1 (directAnnotation).	613	4175
cisbp__M2399	3.13	0.0267		331	1981
hdpi__GTF3C5	3.13	0.0267	GTF3C5 (directAnnotation).	104	541
transfac_pro__M07317	3.13	0.0267	CTBP1 (directAnnotation).	740	4816
hdpi__TOB2	3.13	0.0267	TOB2 (directAnnotation).	500	3134
neph__UW.Motif.0523	3.12	0.0267		144	752
transfac_pro__M04924	3.11	0.0267	EBF1 (directAnnotation).	667	4383
dbcorrdb__PBX3__ENCSR000BGR_1__m3	3.1	0.0267	PBX3 (directAnnotation).	667	4492
dbcorrdb__POLR3G__ENCSR000EHQ_1__m4	3.1	0.0267	POLR3G (directAnnotation).	661	4340
neph__UW.Motif.0169	3.1	0.0267		664	4403
cisbp__M2090	3.1	0.0266	ZNF112; ZNF155; ZNF214; ZNF221; ZNF222; ZNF223; ZNF224; ZNF226; ZNF227; ZNF230; ZNF233; ZNF234; ZNF235; ZNF239; ZNF284; ZNF285; ZNF664 (inferredBy_Orthology).	460	2826
cisbp__M4839	3.09	0.0266	KLF17; KLF18 (inferredBy_Orthology).	677	4653
cisbp__M0207	3.08	0.0266	TCF12 (inferredBy_Orthology).	99	501
flyfactorsurvey__CG3065_F1-5_SOLEXA_2.5_FBgn0034946	3.08	0.0266	KLF17; KLF18 (inferredBy_Orthology).	610	4089
taipale_cyt_meth__SP8_NCCACGCCCMYN_eDBD_meth	3.07	0.0266	SP8 (directAnnotation).	448	2986
transfac_pro__M07628	3.07	0.0266	TFAP2A; TFAP2B; TFAP2C; TFAP2D; TFAP2E	97	500
cisbp__M5923	3.07	0.0266	TFAP2C (directAnnotation).	95	485
jaspar__MA0285.1	3.07	0.0266	ZNF112; ZNF155; ZNF214; ZNF221; ZNF222; ZNF223; ZNF224; ZNF226; ZNF227; ZNF230; ZNF233; ZNF234; ZNF235; ZNF239; ZNF284; ZNF285; ZNF664 (inferredBy_Orthology).	427	2581
cisbp__M6488	3.07	0.0265	SREBF2 (directAnnotation).	398	2544
predrem__nrMotif1427	3.07	0.0265		119	640

Supplementary Table S6 (continued)

taipale_cyt_meth__RHOXF1_NGGATCAN_FL_repr	3.06	0.0265	RHOXF1 (directAnnotation).	666	4383
neph__UW.Motif.0176	3.06	0.0265		196	1138
cisbp__M0082	3.06	0.0265	TFAP2A (inferredBy_Orthology).	109	564
jaspar__MA0337.1	3.05	0.0265	EGR1; EGR2; EGR3; EGR4; WT1 (inferredBy_Orthology).	73	340
taipale_cyt_meth__KLF15_RCCACGCCCMYN_eDBD_meth	3.05	0.0265	KLF15 (directAnnotation).	227	1349
elemento__TGGGGGC	3.05	0.0265		97	486
transfac_pro__M08872	3.05	0.0265	BHLHE40; BHLHE41 (directAnnotation).	390	2453
cisbp__M0406	3.04	0.0265	ZBTB7B (inferredBy_Orthology).	278	1725
dbcorrdb__SMARCA4__ENCSR000EZC_1__m9	3.04	0.0265	SMARCA4 (directAnnotation).	364	2192
dbcorrdb__SP1__ENCSR000BKO_1__m2	3.04	0.0265	SP1 (directAnnotation).	372	2445
transfac_pro__M01961	3.04	0.0264	EGR1; EGR2; EGR3; EGR4; WT1 (inferredBy_Orthology).	73	340
transfac_pro__M04931	3.03	0.0264	TCF12 (directAnnotation).	647	4067
hocomoco__HTF4_MOUSE.H11MO.0.A	3.03	0.0264	TCF12 (inferredBy_Orthology).	587	4091
transfac_pro__M00915	3.03	0.0264	TFAP2A; TFAP2B; TFAP2C (directAnnotation).	77	375
cisbp__M2786	3.03	0.0264		500	3386
transfac_pro__M01048	3.02	0.0264		353	2287
cisbp__M6409	3.02	0.0264	PAX5 (directAnnotation).	424	2771
hocomoco__COT1_HUMAN.H11MO.0.C	3.02	0.0264	NR2F1 (directAnnotation).	157	787
elemento__CCTGCCTC	3.02	0.0264		671	4457
transfac_pro__M01646	3.02	0.0264	ZNF112; ZNF155; ZNF214; ZNF221; ZNF222; ZNF223; ZNF224; ZNF226; ZNF227; ZNF230; ZNF233; ZNF234; ZNF235; ZNF239; ZNF284; ZNF285; ZNF664 (inferredBy_Orthology).	544	3516
transfac_pro__M03868	3.01	0.0264	TFCP2 (directAnnotation).	692	4766
transfac_pro__M02111	3.01	0.0264	RARB (directAnnotation).	107	576
hocomoco__SRBP2_HUMAN.H11MO.0.B	3.01	0.0264	SREBF2 (directAnnotation).	427	2756
hdpi__ZNF313	3.01	0.0263	RNF114 (directAnnotation).	206	1166
yetfasco__YBR066C_1383	3.01	0.0263		135	702
transfac_public__M00009	3	0.0263		166	965
dbcorrdb__NFYB__ENCSR000DNR_1__m2	3	0.0263	NFYB (directAnnotation).	696	4754
transfac_pro__M03852	3	0.0263	SREBF2 (directAnnotation).	629	4334

Transcription factor analysis was performed via R-package 'RcisTarget'. Motifs with a NES over 3.0 were retained. ICM, inner cell mass; NES, normalized enrichment score; TF, transcription factor; AUC, area under the curve.

Supplementary Table S7. Literature retrieval results for enzymes/proteins that were potentially involved in regulating H3K4me3

Gene	Synonyms	Protein	Source (PMID)
ADNP	*ADNP1, KIAA0784*	Activity dependent neuroprotector homeobox	32714933
ARNTL	*bHLHe5, BMAL1, JAP3, MOP3, PASD3*	Aryl hydrocarbon receptor nuclear translocator like	27055591
ASH1L	*ASH1, ASH1L1, huASH1, KMT2H*	ASH1 like histone lysine methyltransferase	29109511; 20422712
ASH2L	*ASH2, ASH2L1, ASH2L2, Bre2*	ASH2 like histone lysine methyltransferase complex subunit	32160530; 18495928; 32279431; 18026121; 27239938; 24715476; 29498679; 31165508; 29871872
ASXL1	*KIAA0978*	Additional sex combs like 1, transcriptional regulator	30266822; 24255920
ATXN1	*ATX1, D6S504E, SCA1*	Ataxin 1	20967218; 30150325
BCOR	*FLJ20285, KIAA1575*	BCL6 corepressor	31334109
BMI1	*PCGF4, RNF51*	BMI1 proto-oncogene, polycomb ring finger	26942853
CBL	*c-Cbl, CBL2, RNF55*	Cbl proto-oncogene	31002461
CBX8	*HPC3, PC3, RC1*	Chromobox 8	27346354
CCL1	*I-309, P500, SCYA1, SISe, TCA3*	C-C motif chemokine ligand 1	28119673
CCNY	*C10orf9, CBCP1, CFP1*	Cyclin Y	27590438; 30941832
CDC73	*C1orf28, FIHP, HRPT1, HRPT2, parafibromin*	Cell division cycle 73	29871872
CTR9	*KIAA0155, p150TSP, SH2BP1, TSBP*	CTR9 homolog, Paf1/RNA polymerase II complex component	27520958
CUL4A	-	Cullin 4A	24305877; 24360965
CUL4B	-	Cullin 4B	21816345
CXXC1	*CFP1, CGBP, hCGBP, HsT2645, PCCX1, PHF18, SPP1, ZCGPC1*	CXXC finger protein 1	27210293; 31633019; 26352678
DET1	*FLJ10103*	DET1, COP1 ubiquitin ligase partner	32414897
DNMT1	*CXXC9, DNMT, MCMT*	DNA methyltransferase 1	32358021
DNMT3A	-	DNA methyltransferase 3 alpha	29323282
DNMT3B	-	DNA methyltransferase 3 beta	29323282
DNMT3L	*MGC1090*	DNA methyltransferase 3 like	26795243
DPY30	*Cps25, HDPY-30, Saf19*	Dpy-30, histone methyltransferase complex regulatory subunit	23872946; 29498679; 21335234
EPB41L4A	*NBL4*	Erythrocyte membrane protein band 4.1 like 4A	31671345
ESR2	*ER-beta, Erb, NR3A2*	Estrogen receptor 2	28577282
FBXO25	*FBX25*	F-box protein 25	31827076
HBB	*beta-globin, CD113t-C*	Hemoglobin subunit beta	26809286
HIRA	*DGCR1, TUP1, TUPLE1*	Histone cell cycle regulator	28515277
IL13	*ALRH, BHR1, IL-13, MGC116786, MGC116788, MGC116789, P600*	Interleukin 13	29386911
IL6	*BSF2, HGF, HSF, IFNB2, IL-6*	Interleukin 6	31633019
ISL1	*Isl-1, ISLET1*	ISL LIM homeobox 1	30674889; 31186351

Supplementary Table S7 (continued)

KAT8	*FLJ14040, hMOF, MOF, MYST1, ZC2HC8*	Lysine acetyltransferase 8	26091365
KDM1A	*AOF2, BHC110, KDM1, KIAA0601, LSD1*	Lysine demethylase 1A	24495580; 30105631; 22067449; 16987819; 29156705
KDM2A	*CXXC8, DKFZP434M1735, FBL11, FBL7, FBXL11, FLJ00115, JHDM1A, KIAA1004, LILINA*	Lysine demethylase 2A	31172793
KDM2B	*CXXC2, Fbl10, FBXL10, JHDM1B, PCCX2*	Lysine demethylase 2B	31197256; 30060056; 30210666; 28706445; 30233643; 31041569; 22825849
KDM3A	*JHMD2A, JMJD1, JMJD1A, KIAA0742, TSGA*	Lysine demethylase 3A	28440295; 28734980
KDM3B	*C5orf7, JMJD1B, KIAA1082, NET22*	Lysine demethylase 3B	28440295
KDM5A	*JARID1A, RBBP2*	Lysine demethylase 5A	20406991; 24442343; 23884959; 27253695; 25673502; 27512956; 25686748; 31985200; 32208897; 33010254; 31061100; 28572115; 33087165; 27899593; 29764755; 31289306; 24619213; 18483221; 29059406
KDM5B	*CT31, JARID1B, PLU-1, PPP1R98, RBBP2H1A*	Lysine demethylase 5B	23884959; 25909289; 31152465; 21821892; 25596733; 23408432; 26739753; 31914649; 30448242; 27626382; 33160990; 28827149; 32868382; 30940185; 24100015; 28402433; 24495580; 21448134; 24412361; 22371483; 31289306; 22420752; 23637629; 22020125; 21369698; 17320160
KDM5C	*DXS1272E, JARID1C, MRX13, SMCX, XE169*	Lysine demethylase 5C	18697827; 21725364; 27498878; 17320160; 27058665; 28630052; 33042830; 26804915; 31334109; 24561620; 24183790; 31691806; 31289306; 32732223; 23545502; 23872847
KDM5D	*HY, HYA, JARID1D, KIAA0234, SMCY*	Lysine demethylase 5D	17351630; 31289306; 30826357; 29863497; 24561620; 32732223; 17320160
KDM6A	*UTX*	Lysine demethylase 6A	30872525; 32879445; 32732223
KLF1	*EKLF*	Kruppel like factor 1	31601799; 21610079
KMT2A	*ALL-1, CXXC7, HRX, HTRX1, MLL, MLL1A, TRX1*	Lysine methyltransferase 2A	21335234; 24550525; 26149390; 20422712; 29386911; 25457206; 30753586; 24183790; 22279536; 26352678; 31551408
KMT2B	*CXXC10, HRX2, KIAA0304, MLL1B, MLL2, MLL4, TRX2, WBP7*	Lysine methyltransferase 2B	32879445; 24619213; 30753586; 22279536; 20433758; 29036642; 17178841; 32393859; 18495928; 17166833; 30753586
KMT2C	*HALR, KIAA1506, MLL3*	Lysine methyltransferase 2C	26489893; 30753586; 22279536; 27926873
KMT2D	*ALR, CAGL114, MLL2, MLL4, TNRC21*	Lysine methyltransferase 2D	31334109; 29861161
KMT2E	*HDCMC04P, MLL5*	Lysine methyltransferase 2E	23629655; 33824267; 24130829; 32509400
LMNA	*CMD1A, HGPS, LGMD1B, LMN1, LMNL1, MADA, PRO1*	Lamin A/C	31912614
LOXL2	*LOR, WS9-14*	Lysyl oxidase like 2	29339785
MEF2C	-	Myocyte enhancer factor 2C	27239938
MEN1	-	Menin 1	25537453
MKL1	*BSAC, KIAA1438, MAL, MKL, MRTF-A*	Megakaryoblastic leukemia (translocation) 1	28298643
MORF4L1	*Eaf3, HsT17725, MEAF3, MORFRG15, MRG15*	Mortality factor 4 like 1	21448134
MYC	*bHLHe39, c-Myc, MYCC*	MYC proto-oncogene, bHLH transcription factor	22371483; 19915707
NFE2L2	*NRF2*	Nuclear factor, erythroid 2 like 2	32715783; 27784786

Supplementary Table S7 (continued)

ORC2	*ORC2L*	Origin recognition complex subunit 2	27052177
PAF1	*F23149_1, FLJ11123, PD2*	PAF1 homolog, Paf1/RNA polymerase II complex component	24360965
PAQR3	-	Progestin and adipoQ receptor family member 3	25706881
PARP1	*ADPRT, PARP, PPOL*	Poly(ADP-ribose) polymerase 1	22053002; 20832725
PAX6	*AN, AN2, D11S812E, WAGR*	Pax6	27617035
PCGF6	*MBLR, RNF134*	Polycomb group ring finger 6	27498878
PHF20	*C20orf104, dJ1121G12.1, TDRD20A*	PHD finger protein 20	28808306; 29452418
PHF8	*JHDM1F, KDM7B, KIAA1111, ZNF422*	PHD finger protein 8	20208542; 20023638; 28734980
POLR2A	*POLR2, POLRA, RPB1*	RNA polymerase II subunit A	18042645
PRDM9	*KMT8B, MSBP3, PFM6, ZNF899*	PR/SET domain 9	31562180; 22028627
PRMT5	*HRMT1L5, SKB1, SKB1Hs*	Protein arginine methyltransferase 5	22231400
PRMT6	*FLJ10559, HRMT1L6*	Protein arginine methyltransferase 6	26848759; 18077460; 22777353; 32047419
PRMT7	*FLJ10640, KIAA1933*	Protein arginine methyltransferase 7	22231400
RBBP5	*RBQ3, SWD1*	RB binding protein 5, histone lysine methyltransferase complex subunit	24550525; 24715476; 29498679; 29871872
RBP2	*CRABP-II, CRBP2, CRBPII, RBPC2*	Retinol binding protein 2	17320160; 24726409; 21604327; 26811384; 27578789; 22012258; 32160530
SALL4	*dJ1112F19.1, ZNF797*	Spalt like transcription factor 4	28974232
SDC1	*CD138, SDC, SYND1, syndecan*	Syndecan 1	29871872
SETD1A	*KIAA0339, KMT2F, Set1*	SET domain containing 1A	21335234; 28298643; 28028175; 32663628; 23353889; 22843687; 22067449; 28515277; 24247718; 30753586; 26352678; 25373480
SETD1B	*KIAA1076, KMT2G, Set1B*	SET domain containing 1B	24081332; 30753586; 26352678; 32094334
SETD7	*KIAA1717, KMT7, SET7, SET7/9, Set9*	SET domain containing lysine methyltransferase 7	30674889
SIN3A	*DKFZP434K2235, KIAA0700*	SIN3 transcription regulator family member A	28028175
SIRT1	*SIR2L1*	Sirtuin 1	24672028; 25485577
SKP2	*FBL1, FBXL1, p45*	S-phase kinase associated protein 2	25596733
SMYD3	*KMT3E, ZMYND1, ZNFN3A1*	SET and MYND domain containing 3	30646949; 29746925; 32779886; 22194464; 26350217; 26350214
SPP1	*BNSP, BSPI, ETA-1, OPN*	Secreted phosphoprotein 1	23511748; 29444070; 29871872
SSBP3	*CSDP, FLJ10355, SSDP, SSDP1*	Single stranded DNA binding protein 3	31186351
STAT1	*ISGF-3, STAT91*	Signal transducer and activator of transcription 1	30231913
STAT2	*STAT113*	Signal transducer and activator of transcription 2	30231913
SUB1	*p14, p15, PC4*	SUB1 homolog, transcriptional regulator	26350217
SUPT6H	*KIAA0162, SPT6H, SPT6*	SPT6 homolog, histone chaperone	22843687
TET1	*bA119F7.1, CXXC6, KIAA1676, LCX*	Tet methylcytosine dioxygenase 1	31551408

Supplementary Table S7 (continued)

TET2	*FLJ20032, KIAA1546*	Tet methylcytosine dioxygenase 2	23353889
TRIM28	*KAP1, PPP1R157, RNF96, TF1B, TIF1B*	Tripartite motif containing 28	23169648
UHRF1	*FLJ21925, ICBP90, Np95, RNF106, TDRD22*	Ubiquitin like with PHD and ring finger domains 1	32358021
USF1	*bHLHb11, MLTFI, UEF*	Upstream transcription factor 1	23754954
UTY	*KDM6AL, KDM6C*	Ubiquitously transcribed tetratricopeptide repeat containing, Y-linked	32732223
WDR5	*CFAP89, SWD3*	WD repeat domain 5	29649410; 31844669; 28487115; 32440219; 24715476; 27346354; 29498679; 29871872
YY1	*DELTA, INO80S, NF-E1, UCRBP, YIN-YANG-1*	YY1 transcription factor	28580685
ZFP36L2	*BRF2, ERF2, RNF162C, TIS11D*	ZFP36 ring finger protein like 2	29408237
ZNF479	*KR19*	Zinc finger protein 479	31138789

Supplementary Table S8. Transcription factor lists in the Venn diagrams.

Transcription factor lists in Supplementary Information Fig. S3 Venn diagram (a)	
90 elements included exclusively in "4-cell":	ATF3, ATF4, BARHL1, CDX2, CEBPA, CEBPB, CEBPD, CEBPE, CEBPG, CNOT6, CTCF, DBP, DDIT3, ELF1, ELF2, ELF4, EN1, EN2, ESRRB, ETV2, ETV7, FOXC1, FOXC2, FOXS1, GATA1, GLIS1, HIC2, HNF1A, HNF4G, HOMEZ, HOXA13, HOXB2, JUN, KLF16, KLF7, LEF1, MAFA, MEIS1, MGA, MYC, NRL, ONECUT1, ONECUT2, ONECUT3, OTX1, OVOL1, PAX3, PAX7, POU4F1, POU4F2, POU4F3, POU6F1, PRDM5, RFX5, SETDB1, SOX1, SOX10, SOX2, STAT5A, TBP, TBX15, TGIF1, TGIF2, TGIF2LX, TGIF2LY, TRIM28, ZBTB11, ZBTB12, ZFP62, ZFP90, ZNF131, ZNF160, ZNF19, ZNF195, ZNF263, ZNF274, ZNF283, ZNF296, ZNF333, ZNF34, ZNF429, ZNF442, ZNF467, ZNF502, ZNF524, ZNF669, ZNF676, ZNF709, ZNF76, ZNF821
40 elements included exclusively in "8-cell":	BACH1, BACH2, BDP1, BRF1, EZH2, FHL2, GTF2I, KLF3, MAF, MAFF, MTF1, MYCN, NFE2, NFE2L1, NFE2L2, NFE2L3, NFIA, NFIC, NHLH1, NR3C1, OLIG2, PLAG1, SALL2, SEMA4A, SMAD4, SOCS4, TBX3, TCF3, TCF4, ZBTB3, ZNF235, ZNF239, ZNF284, ZNF285, ZNF304, ZNF335, ZNF33A, ZNF362, ZNF664, ZSCAN29
50 elements included exclusively in "ICM":	ARID3A, BHLHE40, BHLHE41, CTBP1, DUS3L, EBF1, EGR1, EGR2, EGR3, EGR4, ESR2, FGF19, GTF3C2, GTF3C5, HLCS, IRF1, JUND, KLF6, MXD4, NR1H2, NR1H3, NR1H4, NR1I2, NR1I3, NR2F6, PDS5A, PIK3C3, PIR, PLAGL1, POLR3G, RARB, RNF114, RORA, RXRB, SMPX, SP2, SP4, SPZ1, TAGLN2, TOB2, UBB, UGP2, ZBTB7A, ZBTB7B, ZEB1, ZFY, ZNF121, ZNF358, ZNF768, ZNF787
14 common elements in "4-cell" and "8-cell":	CHD1, GCM1, GCM2, HES7, KLF13, MAFB, MAFG, MAFK, MZF1, OTX2, OVOL2, RAX, RELA, ZNF425
21 common elements in "4-cell" and "ICM":	CUX1, HNF4A, KDM5A, KLF17, KLF18, NR2F1, NR2F2, PBX1, PBX3, RARA, RHOXF1, RXRA, SP3, SP6, SP7, SP8, SP9, TFAP2A, TFAP2B, TFAP2D, TFAP2E
50 common elements in "8-cell" and "ICM":	AFF4, AKR1A1, APEX1, CBX7, CEBPZ, E2F1, GTF3A, H2AFY, HIVEP2, IKZF1, IRF3, KLF2, KLF4, KLF5, MTHFD1, NAP1L1, NFYB, NXPH3, PAX5, POLR3A, PURA, RAB7A, SMAD3, SMAD5, SMAP2, SMARCA4, SMC3, SP1, TFCP2, TRIM21, TRMO, UBP1, WT1, ZFX, ZNF112, ZNF155, ZNF214, ZNF221, ZNF222, ZNF223, ZNF226, ZNF227, ZNF230, ZNF233, ZNF234, ZNF503, ZNF770, ZXDA, ZXDB, ZXDC
17 common elements in "4-cell", "8-cell" and "ICM":	CRX, EOMES, EP300, HNF1B, KLF15, PITX1, PITX2, PITX3, POLR2A, RAD21, RARG, SREBF1, SREBF2, TCF12, TFAP2C, ZNF224, ZNF384
Transcription factor lists in Supplementary Information Fig. S3 Venn diagram (b)	
88 elements included exclusively in "4-cell":	ATF3, ATF4, BARHL1, CDX2, CEBPA, CEBPB, CEBPD, CEBPE, CEBPG, CNOT6, CTCF, DBP, DDIT3, ELF1, ELF2, ELF4, EN1, EN2, ESRRB, ETV2, ETV7, FOXC1, FOXC2, FOXS1, GATA1, GLIS1, HIC2, HNF1A, HNF4G, HOMEZ, HOXA13, HOXB2, JUN, KLF16, KLF7, LEF1, MAFA, MEIS1, MGA, NRL, ONECUT1, ONECUT2, ONECUT3, OTX1, OVOL1, PAX3, PAX7, POU4F1, POU4F2, POU4F3, POU6F1, PRDM5, RFX5, SETDB1, SOX1, SOX10, SOX2, STAT5A, TBP, TBX15, TGIF1, TGIF2, TGIF2LX, TGIF2LY, ZBTB11, ZBTB12, ZFP62, ZFP90, ZNF131, ZNF160, ZNF19, ZNF195, ZNF263, ZNF274, ZNF283, ZNF296, ZNF333, ZNF34, ZNF429, ZNF442, ZNF467, ZNF502, ZNF524, ZNF669, ZNF676, ZNF709, ZNF76, ZNF821
20 common elements in "4-cell" and "ICM":	CUX1, HNF4A, KLF17, KLF18, NR2F1, NR2F2, PBX1, PBX3, RARA, RHOXF1, RXRA, SP3, SP6, SP7, SP8, SP9, TFAP2A, TFAP2B, TFAP2D, TFAP2E
1 common elements in "4-cell", "ICM" and "Literature Retrieval":	KDM5A
2 common elements in "4-cell" and "Literature Retrieval":	MYC, TRIM28
14 common elements in "4-cell" and "8-cell":	CHD1, GCM1, GCM2, HES7, KLF13, MAFB, MAFG, MAFK, MZF1, OTX2, OVOL2, RAX, RELA, ZNF425

Supplementary Table S8 (continued)

16 common elements in "4-cell", "8-cell" and "ICM":	CRX, EOMES, EP300, HNF1B, KLF15, PITX1, PITX2, PITX3, RAD21, RARG, SREBF1, SREBF2, TCF12, TFAP2C, ZNF224, ZNF384
1 common elements in "4-cell", "8-cell", "ICM" and "Literature Retrieval":	POLR2A
0 common element in "4-cell", "8-cell" and "Literature Retrieval":	-
39 elements included exclusively in "8-cell":	BACH1, BACH2, BDP1, BRF1, EZH2, FHL2, GTF2I, KLF3, MAF, MAFF, MTF1, MYCN, NFE2, NFE2L1, NFE2L3, NFIA, NFIC, NHLH1, NR3C1, OLIG2, PLAG1, SALL2, SEMA4A, SMAD4, SOCS4, TBX3, TCF3, TCF4, ZBTB3, ZNF235, ZNF239, ZNF284, ZNF285, ZNF304, ZNF335, ZNF33A, ZNF362, ZNF664, ZSCAN29
50 common elements in "8-cell" and "ICM":	AFF4, AKR1A1, APEX1, CBX7, CEBPZ, E2F1, GTF3A, H2AFY, HIVEP2, IKZF1, IRF3, KLF2, KLF4, KLF5, MTHFD1, NAP1L1, NFYB, NXPH3, PAX5, POLR3A, PURA, RAB7A, SMAD3, SMAD5, SMAP2, SMARCA4, SMC3, SP1, TFCP2, TRIM21, TRMO, UBP1, WT1, ZFX, ZNF112, ZNF155, ZNF214, ZNF221, ZNF222, ZNF223, ZNF226, ZNF227, ZNF230, ZNF233, ZNF234, ZNF503, ZNF770, ZXDA, ZXDB, ZXDC
0 common element in "8-cell", "ICM" and "Literature Retrieval":	-
1 common element in "8-cell" and "Literature Retrieval":	NFE2L2
49 elements included exclusively in "ICM":	ARID3A, BHLHE40, BHLHE41, CTBP1, DUS3L, EBF1, EGR1, EGR2, EGR3, EGR4, FGF19, GTF3C2, GTF3C5, HLCS, IRF1, JUND, KLF6, MXD4, NR1H2, NR1H3, NR1H4, NR1I2, NR1I3, NR2F6, PDS5A, PIK3C3, PIR, PLAGL1, POLR3G, RARB, RNF114, RORA, RXRB, SMPX, SP2, SP4, SPZ1, TAGLN2, TOB2, UBB, UGP2, ZBTB7A, ZBTB7B, ZEB1, ZFY, ZNF121, ZNF358, ZNF768, ZNF787
1 common element in "ICM" and "Literature Retrieval":	ESR2
90 elements included exclusively in "Literature Retrieval":	ADNP, ARNTL, ASH1L, ASH2L, ASXL1, ATXN1, BCOR, BMI1, CBL, CBX8, CCL1, CCNY, CDC73, CTR9, CUL4A, CUL4B, CXXC1, DET1, DNMT1, DNMT3A, DNMT3B, DNMT3L, DPY30, EPB41L4A, FBXO25, HBB, HIRA, IL13, IL6, ISL1, KAT8, KDM1A, KDM2A, KDM2B, KDM3A, KDM3B, KDM5B, KDM5C, KDM5D, KDM6A, KLF1, KMT2A, KMT2B, KMT2C, KMT2D, KMT2E, LMNA, LOXL2, MEF2C, MEN1, MKL1, MORF4L1, ORC2, PAF1, PAQR3, PARP1, PAX6, PCGF6, PHF20, PHF8, PRDM9, PRMT5, PRMT6, PRMT7, RBBP5, RBP2, SALL4, SDC1, SETD1A, SETD1B, SETD7, SIN3A, SIRT1, SKP2, SMYD3, SPP1, SSBP3, STAT1, STAT2, SUB1, SUPT6H, TET1, TET2, UHRF1, USF1, UTY, WDR5, YY1, ZFP36L2, ZNF479

ICM, inner cell mass.
